# Onset of embryonic and placental defects coincide in 19 of 22 novel mid-gestation lethal murine knockout lines

**DOI:** 10.1242/dev.205276

**Published:** 2026-06-02

**Authors:** Taylor M. Guertin, Chip Sisson, Rossella M. Gargiulo, Olivia Macrorie, Emily L. Pease, Hannah E. Garth, Joris Vriens, Katrien De Clercq, Jesse Mager, Kimberly D. Tremblay

**Affiliations:** ^1^ Department of Veterinary and Animal Sciences, University of Massachusetts, Amherst, MA 01003, USA; ^2^ Department of Development and Regeneration, KU Leuven, Leuven 3000, Belgium

**Keywords:** Placenta, Organogenesis, Mid-gestation, Disease Association, Embryonic lethality, Mouse

## Abstract

The focus on assessing embryo-specific defects in lethal knockout (KO) mouse lines has resulted in an underrepresentation of documented placental abnormalities. Presented herein is a uniform analysis of 22 distinct KO mouse lines that exhibit homozygous lethality in a narrow mid-gestation window. All genes altered by each KO have human orthologs, most are implicated in human disease, yet almost all are understudied. To unravel the role each plays in mammalian development, null embryonic and placental phenotype analysis was performed, and wild type (WT) expression of each KO gene was assessed. While the null phenotype of each KO line falls into two broad embryonic categories, the placental phenotypes are diverse and coincide with the onset of gross embryonic defects. The co-occurrence of null embryonic and placental defect onset coupled with WT gene expression highlights that many could be essential in either the embryo or placenta. This analysis serves to guide mid-gestation placental analysis, underscores the importance of routine analysis of the entire conceptus during mid-gestation lethality, and provides functional annotation for each understudied human ortholog.

## Teaser

**Highlighted Article:** A streamlined placental phenotyping pipeline involving histological and molecular analysis of early murine placental morphogenesis uncovers an underappreciated diversity of placental phenotypes in 19 of the 22 novel mid-gestation lethal gene knockouts.

## INTRODUCTION

Despite the success of the human genome sequencing project, the function of most of the protein coding sequences in the mammalian genome remains unknown. To address this gap, the Knockout Mouse Project (KOMP), in collaboration with the International Knockout Mouse Consortium and the International Mouse Phenotyping Consortium (IMPC), aim to generate and phenotype knockout mice for every protein-coding gene in the genome (Groza et al., 2023; Peterson and Murray, 2022). Given the high degree of genetic and biological similarity between mice and humans, this project provides functionally relevant information that can be applied to human biology. The goal of this effort is to elucidate the function of previously understudied genes to better understand the genetic and molecular bases of human disease and to uncover new therapeutic targets to expand the drug discovery pipeline. Herein, the embryonic and extra-embryonic phenotypes of 22 novel knockout (KO) lines that carry loss-of-function mutations and produce homozygous lethality between embryonic day (E)9.5 and E12.5 are examined.

Murine E9.5-E12.5 roughly corresponds to the end of the first trimester in humans and includes the organogenesis stages of development. Accordingly, we hypothesized that each of the genes studied plays a crucial role in essential processes during organogenesis. While anomalies in the developing organ systems might have been expected, surprisingly few malformations, with the exception of those in the cardiovascular system, are causative of intrauterine lethality (Copp, 1995). Intrauterine viability is dependent on receiving sufficient nutrition to support growth and development, which in turn requires proper vascular development, hematopoiesis and the chorioallantoic placenta (Copp, 1995). Despite its crucial role in facilitating embryonic growth and development, placental development has largely been overlooked, leading to a significant underrepresentation of placental defects in gene KO models (Perez-Garcia et al., 2018). A large-scale analysis of KO lines producing lethality during a similar window of murine development suggests that placentation is a bottleneck for development past mid-gestation (Perez-Garcia et al., 2018).

The placenta is an essential transient organ of fetal and maternal origin that supports the growing embryo while also facilitating maternal adaptations necessary for successful pregnancy (Ballasy et al., 2025; Hemberger et al., 2020). In the mouse, placentation initiates at E8.25 when the extra-embryonic mesoderm-derived allantois, extending from the posterior primitive-streak, attaches to the trophectoderm-derived chorion, a process termed chorioallantoic attachment. By E9.5, the three main structural layers of the placenta have formed including the outer decidual layer, derived from the maternal uterine endometrium, and two trophoblast-derived layers, the junctional zone (JZ) and labyrinth zone (LZ). The LZ, proximal to the developing embryo, contains opposing maternal and fetal blood spaces that facilitate nutrient and gas exchange. The JZ, between the maternal decidua and LZ, plays key roles in hormone production. Between E10.0 and E10.5, the placenta becomes responsible for nourishing embryonic growth, and by E12.5 all placental cell types are present.

To capture the progression and key features of placental development, a detailed histological and molecular analysis of normal E8.5-E10.5 placentation was established using a pipeline for parallel analysis of serial sections. This pipeline was used to identify placentation defects in mutant versus controls. Nineteen of 22 KO lines examined exhibited placenta abnormalities that coincide with the onset of gross embryonic phenotypes. While nearly all KO placentas exhibit specific LZ defects, distinct phenotypes are observed in a subset of KOs including spongiotrophoblast (SpT) layer abnormalities within the JZ as well as gross chorioallantoic interface defects and altered trophoblast density within the LZ. In addition to highlighting the essential role of the placenta during mid-gestation, this study advances the functional understanding of 22 previously uncharacterized genes, providing a foundational resource for researchers and clinicians working with omics-data or variants of unknown significance implicating one of these genes.

## RESULTS

### Analysis of E9.5 present/E12.5 absent KO lines reveals two broad categories of embryonic defects

For each of the 22 homozygous lethal lines reported herein, the KOMP production facility demonstrated that KO embryos were present at E9.5 but absent at E12.5 (Table S1). Each line harbors a predicted loss of function allele of a murine gene with a human ortholog. Furthermore, lethality in the stated window is fully penetrant, heterozygotes are viable and fertile, and at the time of acquisition, each allele was considered novel, meaning no previous loss of function allele had been reported in the mouse. For each line, heterozygous intercrosses were performed to obtain E9.5 embryos. If null embryos were grossly abnormal at E9.5, E8.5 mutants were similarly obtained. If E9.5 KO embryos were normal, mutants were obtained at E10.5. A panel of stage-specific gross defects was assessed for at least five litters from each stage. Immunofluorescence of transverse sections from at least three representative mutant and control littermates was performed to assess germ layer formation and neural tube patterning. Mutants from each line, except *Tedc2*, formed all three germ layers and had an appropriately patterned ventral floorplate (data available at https://websites.umass.edu/kdtkomp/) demonstrating that gastrulation initiated properly and that early patterning was intact.

**Fig. 1.**
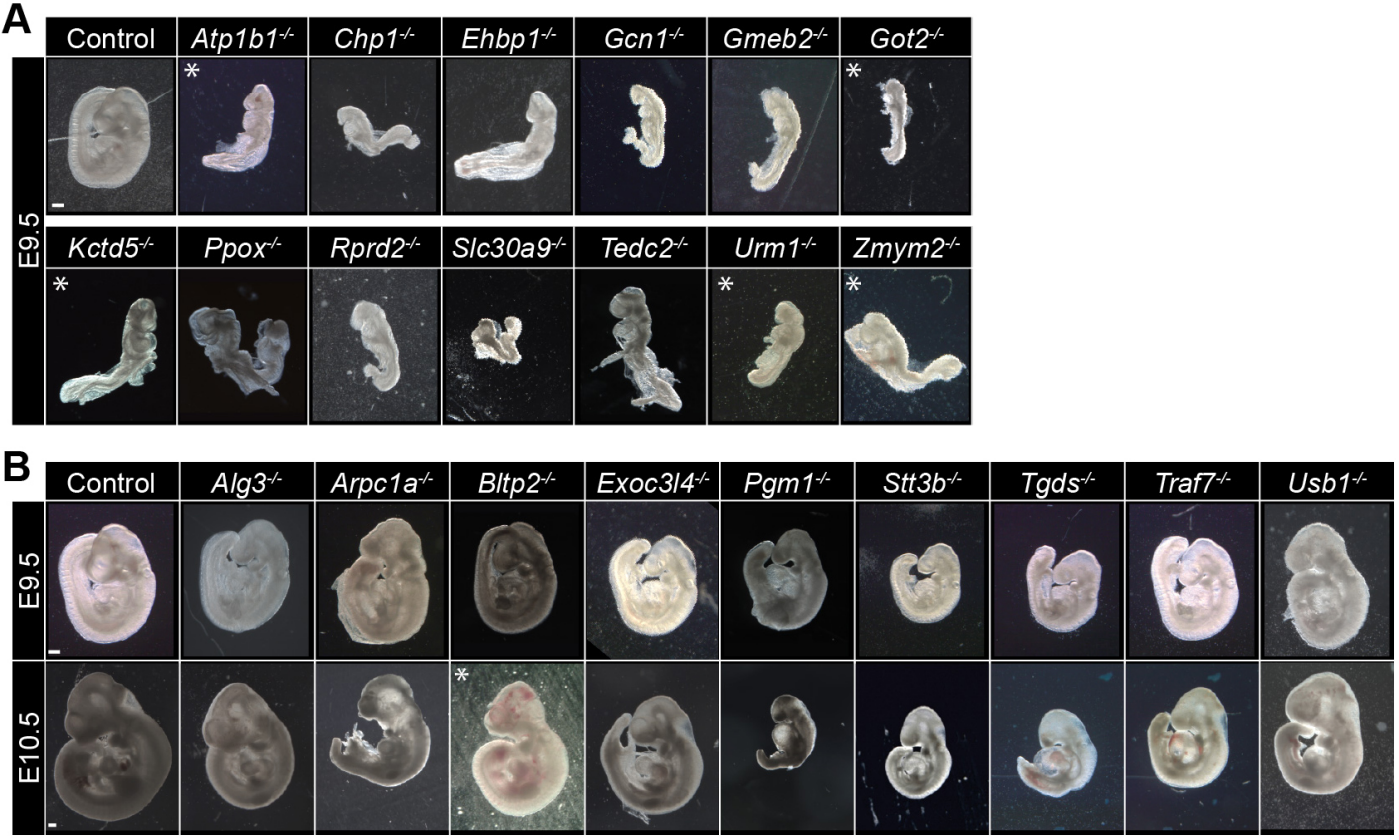
**Representative knockout embryos.** (A,B) Representative control and knockout (KO) embryos from ‘E9.5 onset’ lines at E9.5 (A) and ‘E10.5 onset’ at E9.5 and E10.5 (B). Asterisk indicates that some KOs have a less severe phenotype. Scale bars: 250 μm.

The gross embryonic phenotyping results for mutant from all lines are summarized in Fig. S2 and can be found in more detail at https://websites.umass.edu/kdtkomp/. Broadly, the embryonic phenotype produced by each KO line was placed in two overarching categories, determined by gross phenotype onset. The first includes lines that were delayed or small at E8.5. By E9.5, mutants from each of these lines failed to appropriately turn and all mutants from each line displayed gross phenotypes that distinguished them from their littermates (termed ‘E9.5 onset’; *n*=13/22 lines; Fig. 1A). This category included five lines that were uniformly smaller or delayed compared to E8.5 littermates (*Chp1*, *Gcn1*, *Slc30a9*, *Rprd2*, *Urm1*; Fig. 1A; Figs S1A, S2). In the second category, mutants from all lines initiated turning by E9.5. Although the majority of mutants from many of these lines could not be distinguished from their E9.5 littermates, by E10.5 all lines displayed overall gross defects and several experienced a rapid morphological decline (‘E10.5 onset’; *n*=9/22 lines; Fig. 1B; Fig. S2). Together, this analysis classifies the null embryonic phenotypes produced by each of the 22 KO lines analyzed herein into two broad descriptors, henceforth termed ‘E9.5 onset’ or ‘E10.5 onset’.

### Characterization of normal placental development during early murine mid-gestation

#### Analysis pipeline

**Fig. 2.**
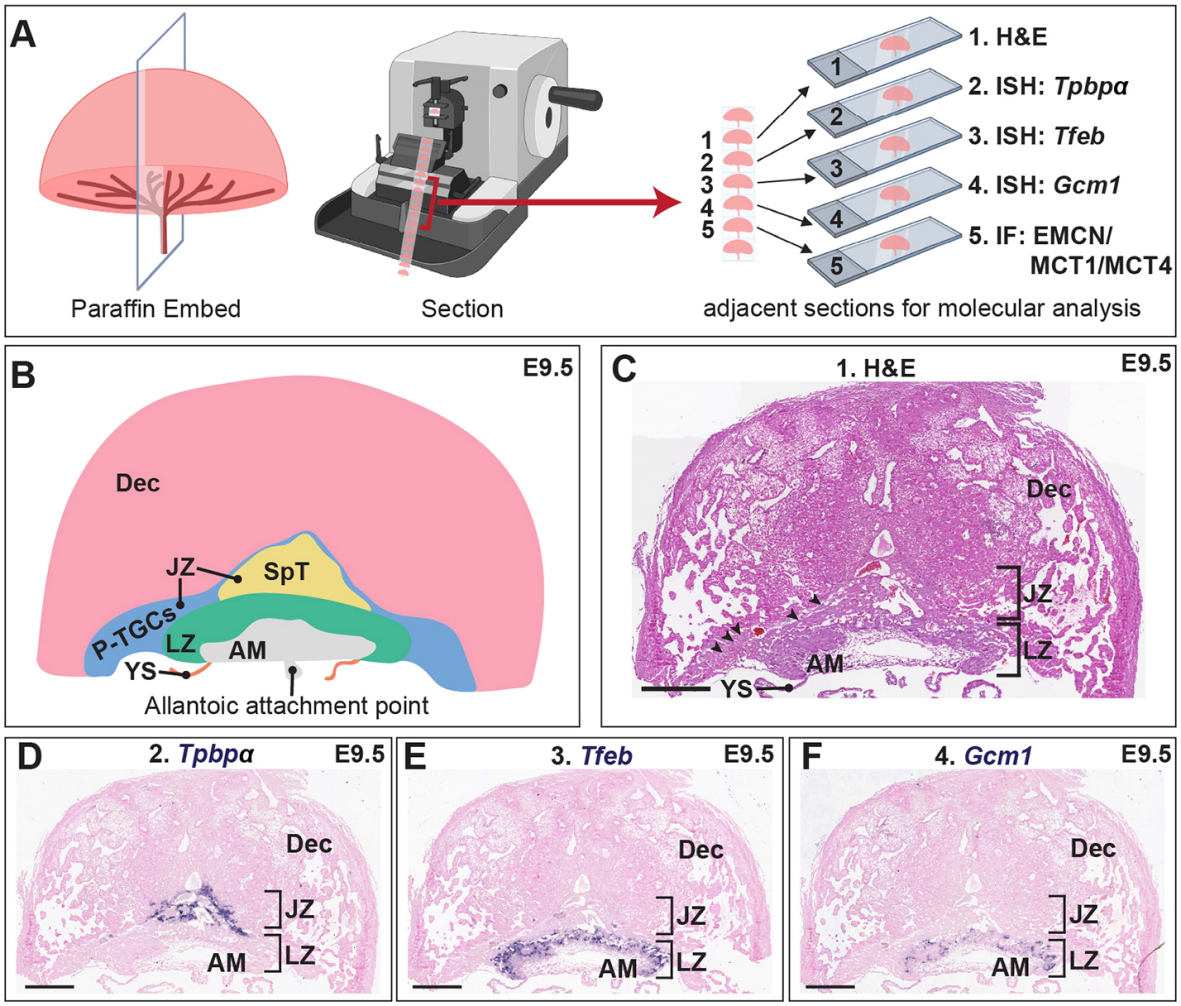
**A streamlined placental analysis pipeline.** (A) Adjacent sections at the midline of knockout (KO) and control placentas are collected for separate analysis (C-F). The cartoon in A was created in BioRender by Guertin, T., 2026. https://biorender.com/9z1k9nr. This figure was sublicensed under CC BY 4.0 terms. (B) Cross sections display the major layers of the E9.5 placenta: AM, allantoic mesenchyme; Dec, maternal decidua; JZ, junctional zone; LZ, labyrinth zone. The allantoic attachment point indicates the placental middle. Yolk sac (YS) remnants are attached to the edge of the chorionic plate. (C) Hematoxylin and Eosin (H&E)-stained sections highlight general morphology, allantoic invasion into the LZ, parietal trophoblast giant cells (P-TGC, arrowhead), maternal blood spaces containing smaller enucleated red blood cells (RBCs) and fetal blood spaces containing larger nucleated RBCs. (D-F) *In situ* hybridization of adjacent E9.5 sections targeting *Tpbpa*, *Tfeb* and *Gcm1*. (D) *Tpbpa* defines the spongiotrophoblast layer (SpT). (E) *Tfeb* identifies a subset of LZ trophoblasts, SynTI cells and their precursors. (F) *Gcm1* marks a subset of SynTII cells and SynTII progenitors. Scale bars: 500 μm.

To examine the effect of early mid-gestation KO mutations on placentation in a reproducible and feasible manner, a placental analysis pipeline was designed with the goal of efficiently evaluating as many key developmental events of placental morphogenesis as possible given the limited number of midline sections available per embryo and the scope of the project (Fig. 2). Sagittal sections through the middle of the placenta, indicated by the allantoic attachment site, were collected and consecutive sections subjected to Hematoxylin and Eosin (H&E) staining, *in situ* hybridization (ISH) with probes recognizing *Tpbpa*, *Tfeb* or *Gcm1* and a final slide subject to immunofluorescence with antibodies recognizing MCT1 (Slc16a1), MCT4 (Slc16a3) and endomucin (Fig. 2A). Such mid-sagittal sections include the maternal decidua, the parietal trophoblast giant cells (P-TGCs) and SpT layer of the JZ and the LZ (Fig. 2B). The LZ is comprised of maternal and fetal blood spaces separated by four distinct cell types: an endothelium, two layers of syncytiotrophoblasts (SynTI and SynTII) and sinusoidal trophoblast giant cells (S-TGCs). H&E-stained sections were assessed for chorioallantoic attachment, allantoic integration into the LZ, maternal and fetal blood spaces, and the P-TGC layer (Fig. 2C). *Tpbpa* was used to identify the SpT layer of the JZ (Fig. 2D; Fig. S3; Hu and Cross, 2011). *Tfeb* and *Gcm1* identify a portion of the LZ. *Tfeb* identifies SynTI cells and their precursors (Fig. 2E; Fig. S3; Marsh and Blelloch, 2020; Steingrímsson et al., 1998), while *Gcm1* identifies SynTII cells and their precursors (Fig. 2F; Fig. S3; Marsh and Blelloch, 2020; Simmons et al., 2008). MCT1 and MCT4 mark mature SynTI and SynTII cells, respectively (Nagai et al., 2010) and endomucin, an endothelial marker, is used to visualize the fetal vasculature.

#### Characterization of placentation during mid-gestation

**Fig. 3.**
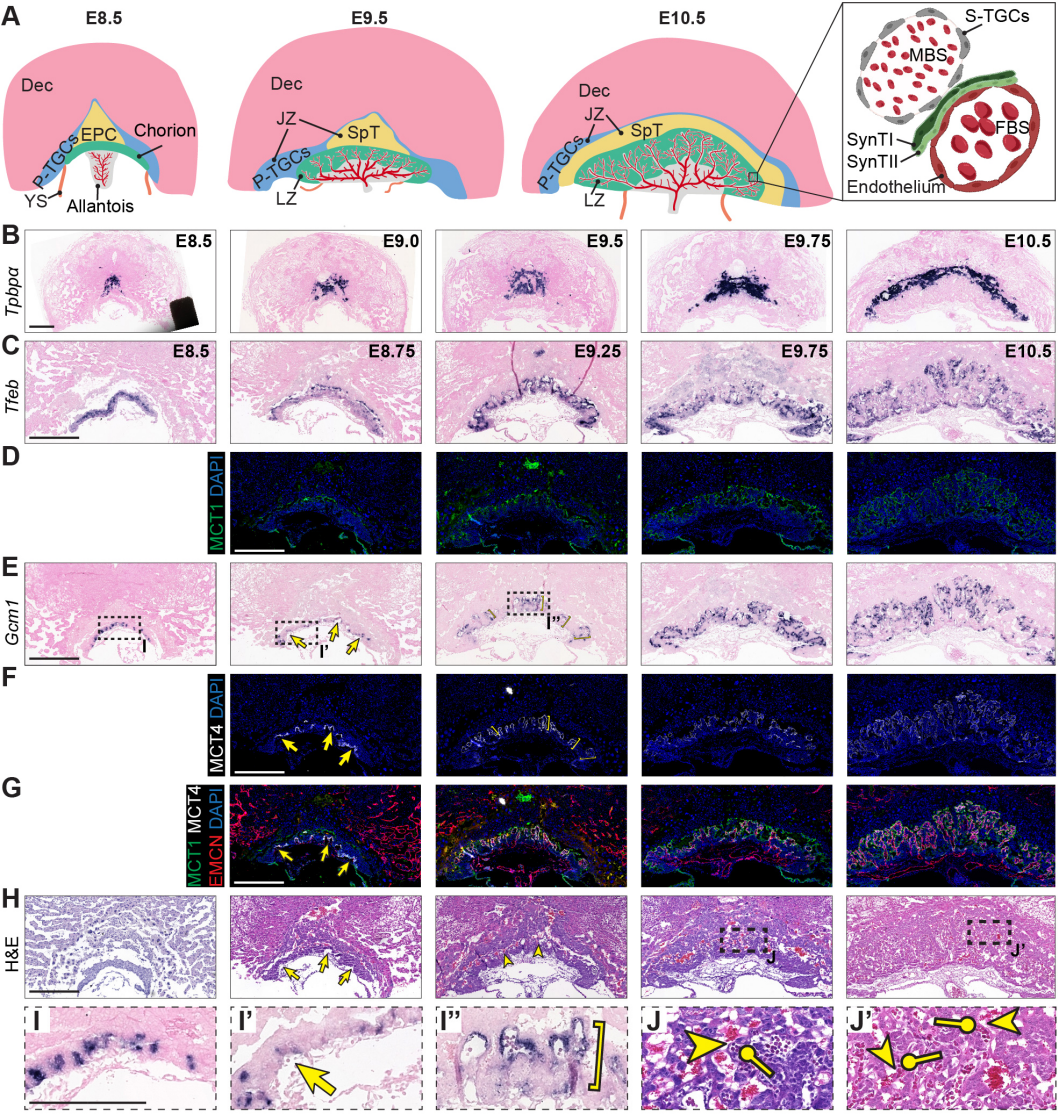
**Analysis of early mid-gestation placentation using a streamlined histological and molecular analysis of the E8.5-E10.5 placenta.** (A) Overview of mid-gestation placental morphogenesis using quarter day staging of E8.5, 9.5 and 10.5 collected conceptuses. At E8.5, the vascularized allantois is attached to the chorion while the ectoplacental cone (EPC), containing spongiotrophoblast (SpT) progenitors, is positioned atop the chorion. Parietal trophoblast giant cells (P-TGCs) line the maternal decidua (Dec) throughout gestation. By E9.5, chorioallantoic branching incorporates allantois-derived vasculature and fetal blood into the labyrinth zone (LZ). At E10.5, differentiated cells of the interhemal barrier include: an endothelium, two tightly apposed layers of syncytiotrophoblasts (SynTI and SynTII) and sinusoidal TGCs (S-TGCs). This barrier separates maternal (MBS) and fetal (FBS) blood spaces within the LZ. The interhemal barrier cartoon in A was created in BioRender by Guertin, T (2026). https://BioRender.com/6jakep4. (B-J′) *In situ* hybridization (ISH), immunofluorescence and Hematoxylin and Eosin (H&E) staining of E8.5-E10.5 control placentas. Because of size limitations, all sections of an E8.5 placenta were subjected to a single ISH probe or H&E staining; however, for E8.75-E10.5 placentas, adjacent sections were used for analysis except as noted. *Tpbpa* expression in E8.5-E10.5 placentas demonstrates the developmental progression of the SpT layer (B). *Tfeb*, MCT1, *Gcm1*, MCT4, endomucin and H&E staining of sectioned E8.5-E10.5 placentas illustrate LZ morphogenesis. Boxes in *Gcm1* and H&E-stained images indicate the LZ region in the magnified panels in I-I″ and J,J′. At E8.5, the SynTI marker *Tfeb* displays uniform expression of the chorion, except for the basal-most layer (C). As the interhemal barrier differentiates, this layer expands to form the LZ. MCT1 is expressed in the SynTI layer of the LZ (D). Unlike *Tfeb*, MCT1 is enriched at the junctional zone (JZ) border and is absent in basal chorionic trophoblasts adjacent to the chorioallantoic interface. At E8.5, clusters of *Gcm1*^+^ cells are observed sporadically along the basal chorion (E,I). At E8.75, signs of chorion involution and invasion of the allantois are observed at sites of *Gcm1* expression (arrows, I′). Villi with an allantois-derived core are outlined by *Gcm1* at the distal end as they elongate through the LZ (bracket, I″). The formation of additional villi and secondary branching morphogenesis contributes to the expansion of the E10.5 *Gcm1*^+^ network. The SynTII marker MCT4 overlaps with *Gcm1* to define chorioallantoic branching and SynTII layer formation (F). However, MCT4 expression is more uniform along the SynTII layer than *Gcm1*. Endomucin (EMCN) immunostaining reveals allantoic-derived fetal vasculature incorporation into the LZ (G). At E8.75, endothelial cells are present in the allantoic mesenchyme integrating into the LZ through chorioallantoic branching, adjacent to the developing SynTII MCT4^+^ layer. As interhemal barrier development proceeds, a more extensive fetal vasculature network is formed. At E10.5, MCT1^+^ SynTI cells are largely adjacent to MCT4^+^ SynTII cells which separate endomucin-lined fetal blood spaces and maternal blood spaces. By E8.75, maternal and fetal blood cells are interspersed among labyrinth trophoblasts (H). Fetal blood cells (lollipop, J,J′), identified by their large, round morphology and Hematoxylin-reactive nuclei, are distinguished from the smaller, enucleated maternal red blood cells. During development, maternal and fetal blood spaces accumulate and become closely juxtaposed (arrowhead, J,J′). Elongated SynT cells of the interhemal barrier are observed between maternal and fetal blood spaces (arrowhead, J,J′). The images used in E9.75 C-H,J, E9.25 C-H,I″ and E10.5 C-H,J′ are the littermate controls also used in Fig. 6B,C and F, respectively. Scale bars: 500 μm (B-E); 250 μm (I-I″,J,J′).

Due to the lack of a single accessible resource describing early placentation and to aid in the analysis of KO placentas, a developmental analysis of normal placentation between E8.5 and E10.5 was produced using the large complement of control placentas generated during this study. This analysis captures a dynamic developmental window, highlights key morphogenic events and illustrates how the E8.5 placental tissues develop into an integrated maternal-fetal interface by E10.5. While a more in-depth description is provided in Figure 1 at https://doi.org/10.6084/m9.figshare.c.8472939), the key events have been summarized in Fig. 3.

##### Key features of junctional zone development

The JZ, consisting of the P-TGC and SpT layer, developed adjacent to the maternal decidua and furthest from the embryo (Fig. 3A). The trophectoderm-derived P-TGC layer directly contacted the maternal decidua (Fig. 3A; Hu and Cross, 2010). P-TGCs, identified in H&E by their large Hematoxylin-reactive nuclei, were most readily observed at the placenta periphery (Fig. 2C, arrowhead). The *Tpbpa*-expressing SpT layer clustered in the core of the ectoplacental cone (EPC) overlying the E8.5 chorion (Fig. 3B). This central *Tpbpa*^+^ cluster expanded towards the placental periphery, forming a uniform layer that overlaid the entire LZ by E10.5 (Fig. 3B).

##### Key features of labyrinth zone morphogenesis

###### Chorioallantoic attachment

LZ morphogenesis initiates upon chorioallantoic attachment at ∼E8.25 (Downs and Gardner, 1995; Hernandez-Verdun, 1974). The allantois emerges from the posterior primitive streak (∼E7.25) and extends into the amniotic cavity to meet the overlying chorion (Inman and Downs, 2007). At the onset of chorioallantoic attachment, the apical chorion was comprised of a layer of *Tfeb*^+^ trophoblasts (E8.5; Fig. 3C). The allantois first attached to discrete sites within the chorion that spread peripherally until it reached the yolk sac (YS) insertion site boundary (E8.5-E9.25; Fig. 3C). Following attachment, the essential LZ morphogenic events include chorioallantoic branching, fetal vascularization, integration of maternal blood spaces and differentiation of the functional exchange unit of the placenta, the interhemal barrier. Each process is described below.

###### Chorioallantoic branching/villi expansion

Chorioallantoic branching, characterized by *Gcm1* expression at various stages, has previously been well described (overview in Simmons, 2014). It is reiterated here, with references to the source primary literature, to correlate the progression of branching morphogenesis with JZ development and other aspects of LZ development.

Chorioallantoic branching ultimately produces the extensive fetal vasculature network in the LZ (Fig. 3A; Cross et al., 2003). This process can be tracked by expression of the SynTII marker *Gcm1*, which initiates before chorioallantoic attachment at E8.25, marking the sites that will undergo branching (Cross et al., 2006; Stecca et al., 2002). At E8.5 *Gcm1* remained restricted to several cell clusters along the basal chorion (Fig. 3E,I). By E8.75, the chorion had involuted toward the decidua at sites of *Gcm1* expression and the vacated space filled with allantoic mesenchyme (E8.75; Fig. 3E,I′, arrow; Cross et al., 2006; Stecca et al., 2002). This movement creates projections, termed primary villi, composed of an inner core of allantoic mesenchyme lined by *Gcm1^+^* chorionic trophoblasts that differentiate into the SynTII layer of the interhemal barrier. These mature SynTII cells express MCT4 (Nagai et al., 2010) and continue to express *Gcm1* (Marsh and Blelloch, 2020).

These *Gcm1*/MCT4^+^ lined primary villi elongated and extended through the LZ, appearing to meet the boundary of the JZ (E9.25; Fig. 3E-G,I″, bracket). By E9.75, secondary branching was apparent, expanding the surface area of the fetal vasculature network, leading to further elaboration by E10.5 (E9.75-E10.5; Fig. 3A,E-G; Cross et al., 2006; Simmons et al., 2008).

###### Fetal vascularization

Fetal blood and the endothelium enter the LZ by chorioallantoic branching (Watson and Cross, 2005). Around the time of chorioallantoic attachment, nucleated fetal blood cells, produced by primitive hematopoiesis in the YS, have entered the developing endothelial network that extends throughout the allantois (Downs, 2022; Downs et al., 1998; Walls et al., 2008). During the early stages of chorioallantoic branching, endothelial cells and fetal blood cells were confined to the base of the chorion, within the invading allantois (E8.75; Fig. 3G,H). However, as the primary villi elongated, fetal blood in endothelial-lined vessels was delivered deeper into the LZ (E9.25-E9.75; Fig. 3G,H,J, lollipop). As secondary branching occurred, an increase in discrete fetal blood spaces was apparent throughout the LZ (E9.75-E10.5; Fig. 3G,H,J′, lollipop).

###### Integration of maternal blood

Maternal blood is delivered through the decidua in specialized vessels, termed spiral arteries. Differentiated TGC-subtypes remodel the spiral arteries and line channels that direct maternal blood through the JZ and into sinusoids of the LZ, where nutrients and gas exchange with fetal blood within the fetal vasculature (Adamson et al., 2002; Simmons et al., 2007). At the onset of maternal blood flow into the fetal compartment of the placenta, enucleated maternal blood cells initially pooled between the JZ and chorion in the region where MCT1^+^ SynTI differentiation was observed (E8.75; Fig. 3H). As chorioallantoic branching proceeded and interhemal barrier differentiation occurred, maternal blood and MCT1^+^ SynTI cells were integrated into the LZ (E9.25-E10.5; Fig. 3D,G,H). By E9.25, adjacent maternal and fetal blood spaces were present, and by E10.5 these opposing blood spaces became more abundant (E9.25-E10.5; Fig. 3H,J,J′, arrowhead). Large, Hematoxylin-reactive nucleated fetal blood cells are easily distinguished from the smaller enucleated maternal blood cells by H&E or via Eosin counterstaining based on their size differential.

###### Interhemal barrier development

Interhemal barrier development coincides with chorioallantoic branching and the entry of fetal and maternal blood into the LZ. This barrier is composed of two layers of syncytiotrophoblasts (SynTI and SynTII) that separate endothelial-lined fetal blood spaces from maternal blood spaces which are bound by a perforated layer of S-TGCs (Fig. 3A; Simmons et al., 2008). Individual layers cannot be identified without specific markers due to their close apposition. SynTII cells and their precursors express *Gcm1* (Fig. 3E; Marsh and Blelloch, 2020; Simmons et al., 2008). SynTII layer development coincides with chorioallantoic branching, as SynTII cells differentiate and outline the allantoic mesenchyme, carrying fetal vasculature, as it integrates into the labyrinth and subsequently expands through further branching. Mature SynTII cells also express MCT4 (Fig. 3F,G; Nagai et al., 2010). While *Gcm1* and MCT4 expression largely overlapped, MCT4 appeared to be more continuous along the SynTII layer, whereas *Gcm1* expression was more discontinuous and appeared to be restricted to a subset of SynTII cells (E9.25-E10.5; Fig. 3E,F). SynTI cells and their precursors expressed *Tfeb* (Fig. 3C; Marsh and Blelloch, 2020). Mature SynTI cells also expressed MCT1 (Fig. 3D,G). *Tfeb* and MCT1 expression appeared to be only partially overlapping. MCT1 was enriched at the JZ border and was absent from the more basal chorionic trophoblasts that express *Tfeb*, suggesting that these basal cells represent the precursors that give rise to the MCT1^+^ SynTI cells (Fig. 3C,D,G).

As interhemal barrier development proceeded, MCT1^+^ SynTI cells and maternal blood initially restricted to the JZ boundary, together with *Gcm1*/MCT4^+^ SynTII-lined fetal vasculature adjacent to the chorioallantoic interface, progressively integrated and extended into the developing labyrinth. By E10.5, an extensive network of adjacent SynTI, SynTII and endothelial cells was observed (Fig. 3G), along with closely apposed maternal and fetal blood spaces (Fig. 3H,J′, arrowhead).

### Histological and molecular analysis reveals common defects among mid-gestation lethal KO placentas

**Fig. 4.**
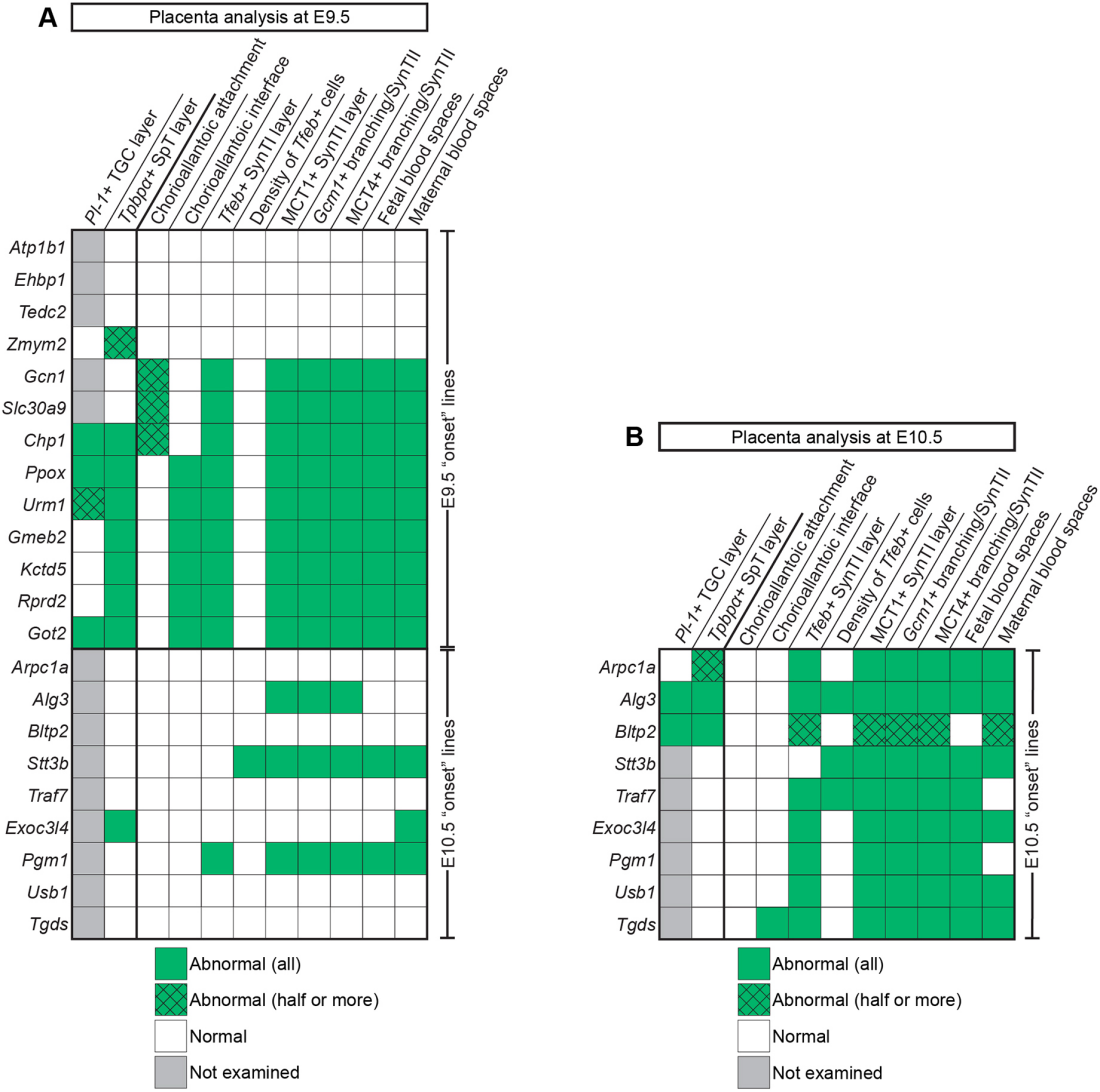
**Summary of placental defects observed in all 22 knockout lines.** (A,B) Knockout (KO) placentas analyzed at E9.5 (A) and E10.5 (B) compared with littermate controls. Each phenotype was scored as present (green) if observed in all placentas analyzed (*n*≥3) while the patterned overlay indicates the phenotype was observed in at least half but not all KO placentas. The junctional zone (JZ) was assessed for changes in the thickness of the *Tpbpa*^+^ spongiotrophoblast layer (SpT) compartment relative to controls. For KO lines that exhibited an abnormal *Tpbpa*^+^ SpT layer, additional placentas were stained for *Pl-1* and trophoblast giant cell (TGC) layer thickness assessed (gray, not assessed). Labyrinth zone (LZ) development was examined as follows: the association of the allantois and chorion (chorioallantoic attachment) and the morphology of the resulting interface was examined by H&E. The thickness of both the *Tfeb*^+^ and MCT1^+^ domains indicates SynTI layer development, while the intensity of *Tfeb* reflects trophoblast density. The extent and depth of *Gcm1* and MCT4 were used to assess chorioallantoic branching and SynTII layer development. H&E is used to assess the presence of discrete maternal and fetal blood spaces interspersed among labyrinth trophoblasts. The relative amounts of maternal and fetal blood in KO versus control placentas could not be reliably assessed qualitatively, except when differences were dramatic. The precise nature of the abnormality variation between KO lines and detailed descriptions are found in Figures 2-23 at https://doi.org/10.6084/m9.figshare.c.8472939.

**Fig. 5.**
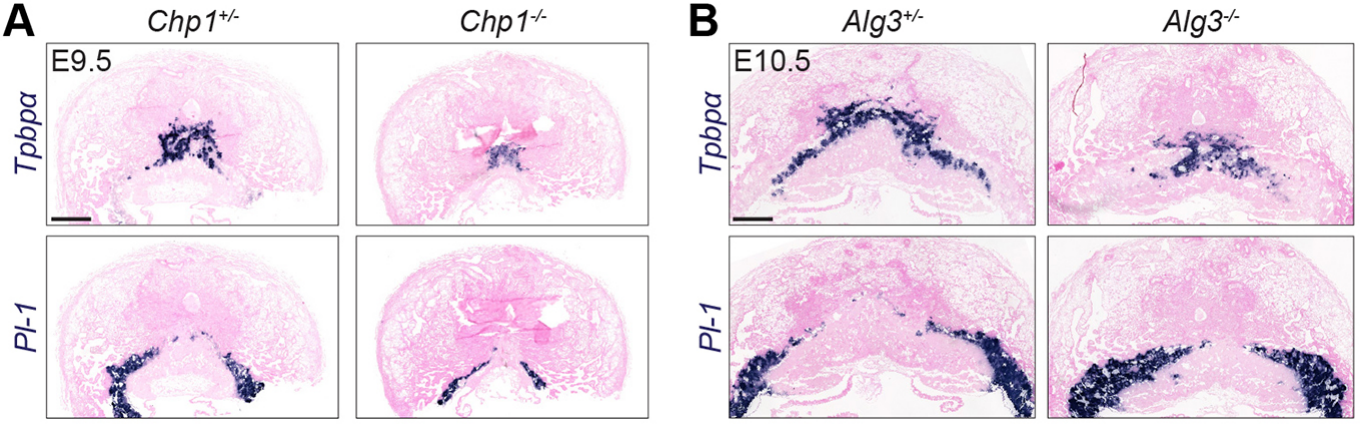
**Junctional zone defects in mid-gestation lethal knockout lines.** (A,B) *Tpbpa* and *Pl-1* expression in adjacent placental sections revealed spongiotrophoblast layer (SpT) and trophoblast giant cell (TGC) layer abnormalities, respectively. (A) E9.5 *Chp1*^−/−^ placentas exhibit reduced SpT and TGC layers compared with controls. (B) E10.5 *Alg3*^−/−^ placentas demonstrate a reduced SpT and an expanded TGC layer. Scale bars: 500 μm.

**Fig. 6.**
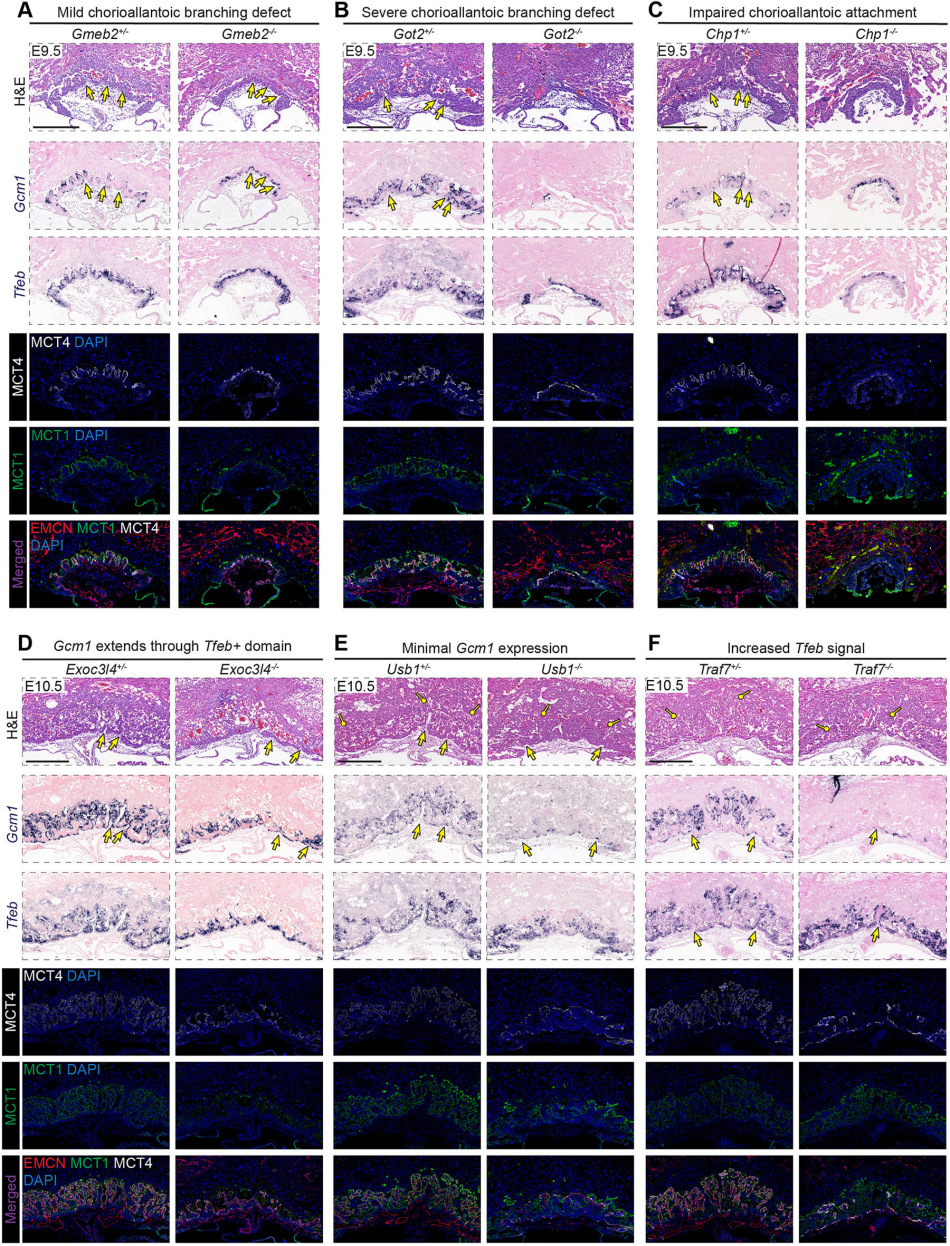
**Labyrinth zone defects in mid-gestation lethal knockout lines.** (A-F) Serial sections of E9.5 (A-C) and E10.5 (D-F) knockout (KO) placentas stained with H&E, probed for *Gcm1* and *Tfeb*, and immunostained for MCT1, MCT4 and endomucin revealed common labyrinth zone (LZ) defects. The *Tfeb*^+^ and MCT1^+^ region of controls has expanded and is interspersed with maternal and fetal blood spaces. Regions of chorionic involution and allantoic mesenchyme movement into the vacated region were observed (arrows) and rudimentary branching marked by *Gcm1*/MCT4. (A) *Gmeb2*^−/−^ placentas exhibited moderate LZ defects. Less allantois-derived tissue was attached to the chorion, demonstrating an abnormal chorioallantoic interface. The *Tfeb*^+^ and MCT1^+^ SynTI region remain abnormally thin, while *Gcm1*/MCT4 expression was reduced compared to controls. The chorion has initiated involution and shallow allantois invasion observed, indicating that primary villi have started to form (arrows) but have not elongated. (B) *Got2*^−/−^ placentas exhibited severe LZ defects. Less allantois is attached to the chorion and the interface has not expanded as widely as controls, demonstrating an abnormal chorioallantoic interface. The *Tfeb*^+^ and MCT1^+^ SynTI region remains abnormally thin and sporadic *Gcm1*/MCT4^+^ cells observed along the chorion, but there is no evidence of chorionic involution and primary villi initiation. These littermate controls images were also used to create the Fig. 3 E9.75 C-H and J panels. (C) *Chp1*^−/−^ placentas exhibit impaired chorioallantoic attachment, with the allantois loosely attached to the chorion. A thin layer of *Tfeb*^+^ and MCT1^+^ cells are observed and while *Gcm1*/MCT4 is expressed along the chorion, there is no evidence of the primary villi initiation. These *Chp1* littermate control images were also used to create the Fig. 3 E9.25 C-H and I″ panels. (D) Compared with controls the labyrinth zone (LZ) of *Exoc3l4*^−/−^ placentas exhibit a reduced *Tfeb* and MCT1^+^ SynTI region and *Gcm1*/MCT4^+^ chorioallantoic branching. Allantois-derived tissue has integrated into the LZ (arrows) and the presence of fetal blood indicates evidence of primary villi elongation. While the depth of elongation, based on *Gcm1* expression, is reduced compared to controls, it extends through the *Tfeb*^+^ region. A reduction in fetal blood spaces and an overabundance of maternal blood is apparent in the LZ*.* (E) LZ defects in *Usb1^−/−^* placentas include a reduction of both the *Tfeb*^+^ and MCT1^+^ SynTI layer and *Gcm1*/MCT4^+^ chorioallantoic branching. Unlike the moderate LZ defects of the *Exoc3l4^−/−^* placentas (D), the depth of *Gcm1*^+^ branching is more severely impaired. Although the chorion has started to fold and the allantois has initiated shallow integration in the LZ, other signs of branching morphogenesis are absent as *Gcm1*^+^ cells are sparse and restricted to the chorioallantoic interface. Strikingly, MCT4^+^ SynTII cells and fetal blood cells (lollipop) are observed beyond the *Gcm1* expression boundary. (F) *Traf7*^−/−^ placentas exhibit similar LZ defects as *Usb1^−/−^* placentas (E), however, the stronger *Tfeb* signal intensity suggests an increased density of *Tfeb*^+^ SynTI precursors. These Traf7 littermate control images were also used to create the Fig. 3 E10.5 C-H and J′ panels. Scale bars: 500 μm.

**Fig. 7.**
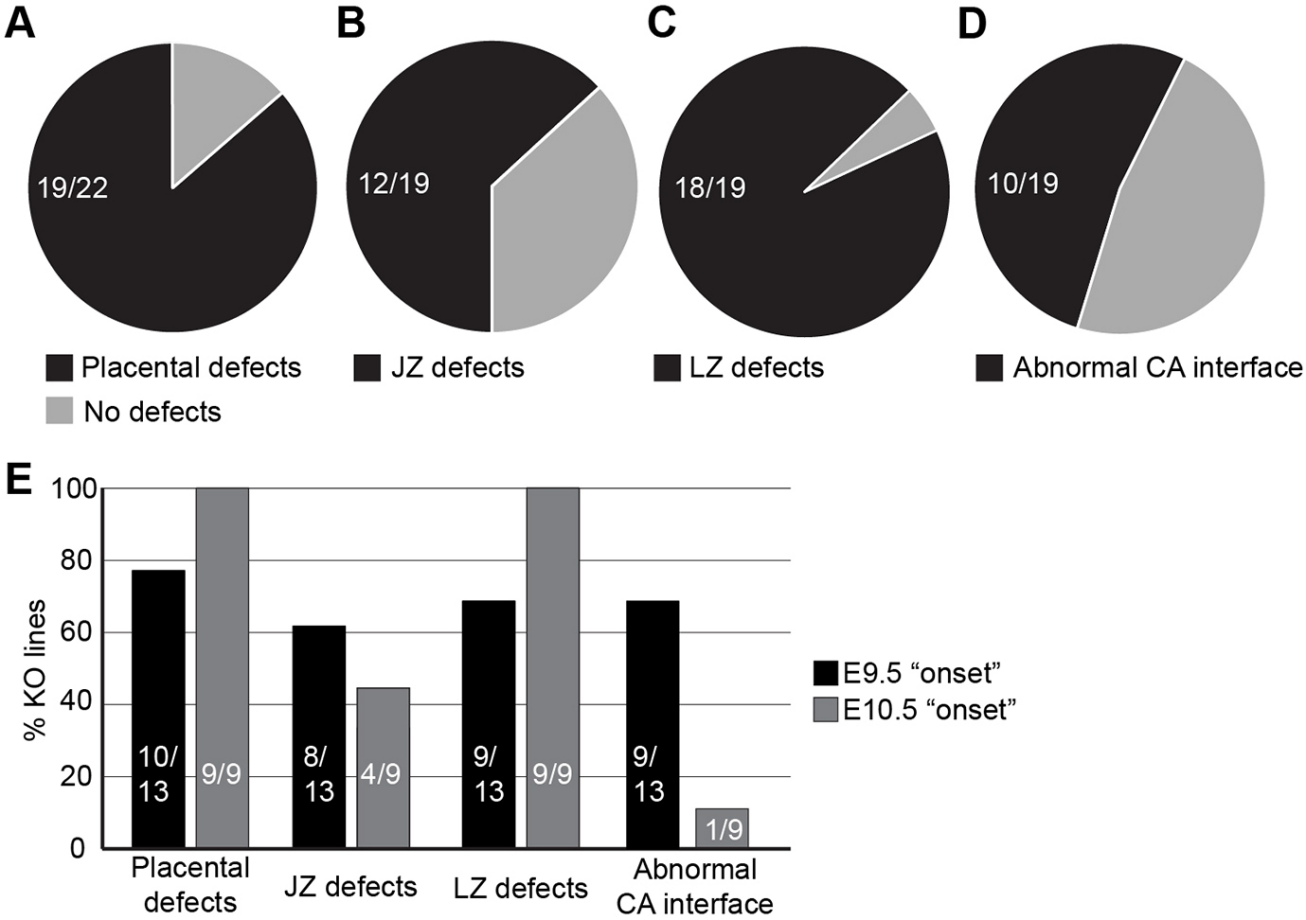
**Placental defects are observed in 86% of the mid-gestation lethal knockout lines examined.** (A-D) Summary of the prevalence of placental defects in the 22 mid-gestation lethal knockout (KO) lines examined. (A) Placental defects occur in 86% (19/22) of KO lines. Of the 19 lines with defects, 63% exhibit junctional zone (JZ) defects (B), 95% display labyrinth zone (LZ) defects (C), and 53% have an abnormal chorioallantoic (CA) interface (D). (E) Comparison of the prevalence of placental defects between the ‘E9.5 onset’ versus ‘E10.5 onset’ lines.

**Fig. 8.**
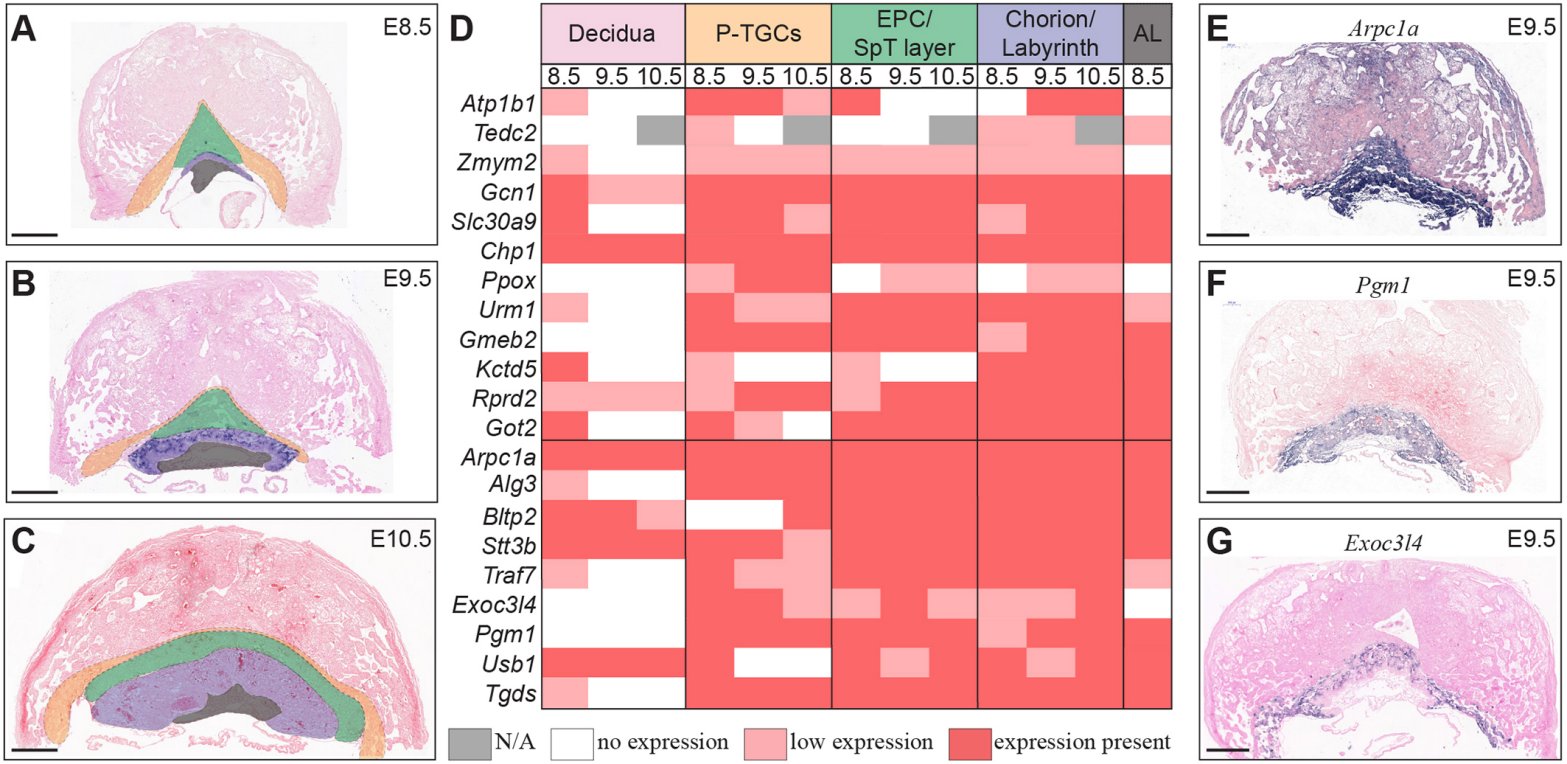
**Most mid-gestation lethal knockout genes examined herein are expressed in the fetal component of wild-type placentas.** (A-C) Regions of the E8.5-E10.5 placenta are overlaid onto histological images: the allantois (grey), the E8.5 chorion becomes the labyrinth zone (LZ) by E9.5 (purple), the E8.5 EPC gives rise to the spongiotrophoblast layer (SpT) layer at E9.5 (green), parietal trophoblast giant cells (P-TGCs; orange), and decidua (pink). After E9.5, the LZ contains both trophoblast and allantois-derived cells which cannot be readily distinguished without co-staining for cell type-specific markers. (D) Gene expression was assessed in key regions of the placenta and classified as readily identifiable (red), low/indistinct (pink), absent (white) or not examined (grey). The horizontal black line separates the ‘E9.5 onset’ lines (top) from the ‘E10.5 onset’ lines (bottom). Allantois (AL) expression was assessed using whole mount *in situ* hybridization of E8.5 embryos. (E) *Arpc1a* is broadly expressed throughout the placenta, including the fetal component (P-TGC, SpT, LZ, allantois) and maternal decidua. (F) *Pgm1* is detected throughout the fetal component. (G) *Exoc3l4* is restricted to a subset of cells within the LZ. The images used in panels E-G are also used in Fig. S6. Scale bars: 500 μm.

The key morphological events described above were assessed in KO placentas and directly compared with littermate controls from all 22 lines at E9.5 (Fig. 4A). The ‘E10.5 onset’ lines were similarly examined at E10.5 (Fig. 4B). Representative images of KO and littermate control placentas of each line, including the number examined, are provided in more detail (Figures 2-23 at https://doi.org/10.6084/m9.figshare.c.8472939). Nineteen of the 22 mid-gestation lines examined (86%) had identifiable placental defects that coincided with the onset of pronounced embryonic defects. All three of the lines with no identified placental defects were in the ‘E9.5 onset’ category (*Atp1b1^−/−^*, *Ehbp1^−/−^*, *Tedc2^−/−^*; Fig. 4A, Figures 2-4 at https://doi.org/10.6084/m9.figshare.c.8472939) and exhibited delay and pronounced embryonic defects at E9.5 (Fig. 1A) suggesting that at least through E9.5, a grossly abnormal embryo does not impact the aspects of placental development examined herein.

#### JZ defects

The size of the *Tpbpa*^+^ SpT layer, the main JZ layer examined, was altered in roughly half of the lines (Fig. 4; *n*=12/22). SpTs and TGCs are hypothesized to arise from a shared precursor, and abnormalities in one layer of mutant placentas often coincide with opposing abnormalities in the other layer, although this relationship is not always observed (Simmons and Cross, 2005). To examine the co-occurrence of P-TGC layer abnormalities, additional available placentas belonging to 11 of the lines with SpT layer abnormalities were examined and adjacent sections were stained with the P-TGC marker *Pl-1* (*Prl3d1*) and *Tpbpa*. A reduced SpT layer was associated with a thinner *Pl-1^+^* P-TGC layer in four lines (Fig. 5A; *Chp1*, *Urm1*, *Got2*, *Bltp2*), a thickened P-TGC layer in two lines (Fig. 5B; *Ppox*, *Alg3*), and a normal P-TGC layer in three lines (*Gmeb2*, *Kctd5*, *Rprd2*; Figures 11–13 at https://doi.org/10.6084/m9.figshare.c.8472939). In the remaining two lines (*Arpc1a and Zmym2*), despite the initial identification of a thickened SpT layer using the analysis pipeline, the additional placentas examined displayed a normal SpT and P-TGC layer (Figures 5 and 15 at https://doi.org/10.6084/m9.figshare.c.8472939). While most KO lines with JZ defects also displayed LZ defects, *Zmym2^−/−^* placentas represented the only KO in which a JZ defect was not accompanied by an LZ defect (Figure 5 at https://doi.org/10.6084/m9.figshare.c.8472939).

#### LZ defects in ‘E9.5 onset’ lines

All nine of the ‘E9.5 onset’ KO lines with LZ defects share several commonalities including a reduced LZ, decreased chorioallantoic branching, impaired SynTI and SynTII differentiation, and the failure of blood to integrate appropriately among the labyrinth trophoblasts. In KO placentas from each of these lines, the *Tfeb*^+^ and MCT1^+^ region failed to expand (Fig. 6A-C). Endomucin^+^ endothelium and fetal blood remained confined to the chorioallantoic interface and maternal blood accumulated abnormally between the JZ and LZ (Fig. 6A-C). While all nine KO placentas displayed reduced *Gcm1*/MCT4^+^ chorioallantoic branching compared with littermate controls, the severity displayed is KO-gene dependent (Fig. 6A-C).

Three of the nine KO lines with severe chorioallantoic branching defects were characterized as having ‘impaired’ chorioallantoic attachment (*Gcn1*, *Slc30a9*, *Chp1*; Fig. 4A). This impairment was not fully penetrant and, in some of the KO placentas examined for each these lines, chorioallantoic attachment was successful, but the resultant interface was abnormal (Figures 6-8 at https://doi.org/10.6084/m9.figshare.c.8472939). *Slc30a9* KO resulted in chorioallantoic attachment failure, as indicated by the lack of allantoic mesenchyme attached to the chorion, in the majority of conceptuses examined (*n*=8/14; Figure 7 at https://doi.org/10.6084/m9.figshare.c.8472939). In contrast, *Chp1* and *Gcn1* KO resulted in impaired chorioallantoic attachment, but not complete failure. In histological sections of *Chp1*^−/−^ and *Gcn1*^−/−^ conceptuses, allantoic mesenchyme was adjacent to the chorion, but the attachment appeared sparse and was easily disrupted during dissection (Fig. 6C; Figures 6 and 8 at https://doi.org/10.6084/m9.figshare.c.8472939). KO embryos from each of these lines exhibited an extended allantois (Fig. S4), suggesting that the attachment defects arose from a mechanism other than failed extension. Furthermore, regardless of the state of chorioallantoic attachment, LZ morphogenesis was severely disrupted, including a reduced *Tfeb*^+^ and MCT1^+^ layer and no evidence of primary villi initiation, as evidenced by the restriction of *Gcm1*/MCT4 to the basal chorion (Fig. 6C, arrows). Notably, impaired chorioallantoic attachment did not appear to directly influence JZ development, as two of these KOs had a normal SpT layer (*Gcn1*, *Slc30a9*; Figures 6 and 7 at https://doi.org/10.6084/m9.figshare.c.8472939).

#### LZ defects in ‘E10.5 onset’ lines

KO placentas from all nine of the ‘E10.5 onset’ lines exhibited LZ defects by E10.5, when all also displayed prominent embryonic phenotypes (Figs 4B and 6D-F). The shared LZ phenotypes included a reduced LZ with impaired SynTI differentiation, decreased *Gcm1*/MCT4^+^ chorioallantoic branching and SynTII development, and fewer adjacent maternal and fetal blood spaces compared with controls (Figs 4B and 6D-F). Although chorioallantoic branching and primary villi formation had initiated, as evidenced by the presence of chorionic involution and the shallow integration of allantoic mesenchyme into the LZ (Fig. 6D-F, arrows), the reduction of *Gcm1*/MCT4^+^ branches indicated impaired villi elongation. It is worth noting that in many of these KO placentas (5 of 9), the *Gcm1*/MCT4^+^ branches that were present appeared to extend normally through the *Tfeb*^+^ SynTI precursor layer to reach the MCT1^+^ SynTI cells, although they were dramatically reduced in number (Fig. 6D). In the remaining lines (4 of 9), *Gcm1*/MCT4 branching failed to traverse the *Tfeb*^+^ SynTI precursor layer and are rarely in contact with MCT1^+^ SynTI cells (Fig. 6E,F). Regardless, the extensive network of adjacent MCT1^+^ SynTI and MCT4^+^ SynTII cells observed in controls was absent in KO placentas from all nine lines, indicating impaired interhemal barrier development. A notable E10.5 placental phenotype, observed in three of the ‘E10.5 onset’ lines (*Alg3*, *Stt3b* and *Traf7*), was an abnormally dense *Tfeb*^+^/MCT1^−^ SynTI precursor layer that was coupled with the loss of interspersed *Tfeb*^−^ cells representing other differentiated cells of the interhemal barrier (Figs 4B and 6F).

A striking observation in many of the ‘E10.5 onset’ lines is the presence of fetal blood and MCT4 SynTII cells beyond the *Gcm1*^+^ branching/SynTII domain (Fig. 6E,F, lollipops). Such localization of fetal blood deeper into the placenta, beyond the *Gcm1*^+^ domain, suggests that primary villi had initially formed and elongated into the LZ. Analysis of KO placentas from the ‘E10.5 onset’ lines at E9.5 demonstrated that rudimentary chorioallantoic branching had initiated at E9.5 (Fig. 4A; Figures 19 and 22 at https://doi.org/10.6084/m9.figshare.c.8472939), suggesting that while villi initially form, they fail to be maintained and regress by E10.5.

Overall, the streamlined approach used herein defined specific placental defects in 86% (19/22) of the novel mid-gestation lethal KO lines examined (Fig. 7A). Among those lines with identified placental defects, 63% (12/19) exhibited JZ defects, 95% (18/19) possessed LZ defects and 53% (10/19) had an abnormal chorioallantoic interface (Fig. 7B-D). While LZ defects were present in almost all KO placentas regardless of the embryonic category, both JZ and chorioallantoic interface abnormalities were more prevalent in KO placentas from lines in the ‘E9.5 onset’ category (Fig. 7E).

### Endogenous expression of the genes altered in the KOs

To complement characterization of the null phenotype for each gene, ISH was performed to examine the endogenous expression pattern of each gene deleted in all KO lines in WT embryo and placentas. Of the 21 genes examined by colorimetric ISH (*Ehbp1* probe was not generated), most (16/21) had relatively widespread embryonic expression (Fig. S5). *Atp1b1*, *Pgm1*, *Ppox* and *Zmym2* exhibit tissue-specific embryonic expression (Fig. S5), while *Exoc3l4* was not detected (Fig. S5). The colorimetric ISH data mainly recapitulated the Mouse Organogenesis Spatiotemporal Transcriptomic Atlas (MOSTA) database and largely mirrored those with published expression during early mid-gestation, validating these newly created probes [(Chen et al., 2022); *Pgm1* and *Tgds*: (Tamplin et al., 2008); *Arpc1a* and *Atp1b1*: (Magdaleno et al., 2006); *Kctd5* and *Zmym2*: (Gray et al., 2004)].

Expression of each gene was also identified in WT placentas using section ISH (Fig. 8A-D; Fig. S6). Almost all (19/21) are expressed in a portion of the trophoblast compartment (Fig. 8D). *Tedc2* and *Zmym2* were not convincingly detected in the placenta (Fig. S6). A few, including *Arpc1a*, are expressed throughout the entire placenta, including the maternal decidua (Fig. 8E). Interestingly most genes examined are not detected or markedly reduced in the maternal decidua (Fig. 8; Fig. S6). Many, including *Pgm1*, display enriched expression solely in the trophoblast compartment of the placenta (Fig. 8F). While almost all genes are expressed throughout the LZ (Fig. 8D), expression of *Atp1b1*, *Chp1*, *Exoc3l4*, *Kctd5* and *Traf7* are LZ specific (Fig. 8G; Fig. S6).

Placental cell-type expression was queried using published mid-gestation placental-derived single cell or single nuclei RNA-sequencing (RNA-seq) databases (Fig. S7; Jiang et al., 2023; Marsh and Blelloch, 2020). These datasets mainly agreed with the colorimetric analysis, while revealing the enrichment of *Atp1b1* in S-TGC precursors and S-TGCs, that *Chp1* was highest in the EPC and moderately expressed in S-TGCs, SynTI and SynTII cells, and the specificity of *Exoc3l4* to S-TGC precursors, S-TGCs and SynTI cells. Unlike their localized expression in the colorimetric *in situs*, *Kctd5* and *Traf7* were uniformly expressed across trophoblast cell subtype clusters at E9.5 in both datasets.

### Mid-gestation lethal KO genes are associated with inherited human disorders

Examination of the Online Mendelian Inheritance in Man (OMIM) and Orphanet databases for genetic disorder associations for each of the 22 genes analyzed herein demonstrate that over half (12/22) are associated with at least one Mendelian inherited disorder (Table S2). Overall, these are multisystem disorders that manifest during gestation or the postnatal period (Fig. S8; Table S2). The most prevalent phenotype abnormalities among the associated disorders belong to the nervous system, head or neck, musculoskeletal system, and metabolism/homeostasis (Fig. S8). Disorders caused by *Slc30a9* and *Traf7* are characterized in part by abnormalities in prenatal development or birth (Fig. S8). Intrauterine growth restriction has been documented in disorders caused by variants in *Slc30a9*, *Stt3b*, *Tgds*, *Traf7*, and *Zmym2*. The only disorder associated with lethality, which includes childhood death, is Congenital disorder of glycosylation type 1x, linked to *Stt3b*.

## DISCUSSION

The null phenotype and endogenous expression of 22 understudied murine genes, each with a human ortholog, is presented herein. The vast majority of the E9.5 present/E12.5 absent KO lines examined have placental defects (19/22; 86%), a rate similar to that of another study that analyzed a wider range of mid-gestation mutants (Perez-Garcia et al., 2018). To facilitate consistent analysis of this understudied window, the histological and molecular pipeline, used to identify KO phenotypes, was also used to document the key features of normal mid-gestation placental development. While other resources describe aspects of placental development, including phenotyping approaches and histology of individual developmental events (Elmore et al., 2022; Natale et al., 2006; Simmons, 2014), no single accessible resource comprehensively documents the concurrent events of E8.5-E10.5 placental development. The pipeline identified a wide variety of placental phenotypes in early mid-gestation KO placentas. For example, while impaired chorioallantoic attachment blocks labyrinth development, such defects do not directly affect the SpT layer, which is properly developed in *Gcn1* and *Slc30a9* KO placentas. Similarly, the higher density of *Tfeb*^+^ SynTI precursors observed in the *Traf7* KO, among others, may contribute to the block in chorioallantoic branching and SynTII differentiation. Finally, it is important to note that mutants from three ‘E9.5 onset’ lines, *Atp1b1*, *Ehbp1* and *Tedc2*, all exhibit severe E9.5 embryonic defects, yet display relatively normal placental development, indicating that, at E9.5, the aspects of placental development analyzed herein can proceed normally despite a grossly abnormal embryo.

The most common placental defects observed in this large complement of mid-gestation lethal KO lines was a reduced LZ (*n*=18/19). This reduction is consistently accompanied by impaired chorioallantoic branching and SynTII layer development, which is essential for interhemal barrier development and the incorporation of the allantois-derived vasculature (Hernandez-Verdun, 1974; Watson and Cross, 2005). Consequently, in most of these lines fetal blood fails to integrate into the LZ and remains confined to the allantoic mesenchyme attached to the chorion (*n*=17/19). Additionally, SynTI layer development is consistently impaired (*n*=18/19), and maternal blood accumulates between the JZ and LZ instead of within the LZ (*n*=16/19). This failure of maternal blood dispersal is likely secondary to impaired chorioallantoic branching, as branching drives interhemal barrier development and distributes maternal blood into discrete sinusoids within the LZ (Adamson et al., 2002).

The severity of the LZ defects correlates with the onset of gross embryonic phenotypes. KO lines categorized as ‘E9.5 onset’ possess a complete or near complete block of LZ morphogenesis, whereas those categorized as ‘E10.5 onset’ display less severe LZ defects at E10.5. Although impaired chorioallantoic branching characterizes all KOs with LZ defects, the severity is KO gene dependent, arresting this process at distinct stages. For example, some ‘E9.5 onset’ lines, such as the *Got2* KO, fail at primary villi initiation, whereas others, such as the *Gmeb2* KO, exhibit primary villi elongation failure. Further examination of these KO placentas may highlight the mechanisms or signals required at each stage. Notably, in many ‘E10.5 onset’ mutant placentas, the labyrinth exhibits minimal to no chorioallantoic branching, as indicated by the lack of *Gcm1* expression. However, the presence of fetal blood interspersed among labyrinth trophoblasts suggests that branching initiated, but the villi subsequently regressed. In support of this hypothesis, examination of KO placentas from these lines at E9.5 revealed that most displayed normal chorioallantoic branching. The mechanism underlying the failed maintenance and subsequent regression of the E9.5 villi remains to be determined.

The placenta becomes functional at E10.0-E10.5, assuming primary responsibility for supporting embryonic growth. This transition is evidenced by the establishment of uteroplacental and fetoplacental circulation, accompanied by the shift in blood flow from the YS-embryo loop to a more dominant placenta-embryo circulatory connection (Adamson et al., 2002; Croy et al., 2012; Kalisch-Smith, 2025; Walls et al., 2008). Notably, many placenta and trophoblast-specific mutations produce structural labyrinth defects that impair the ability for fetal and maternal blood to interface and therefore appropriate functioning of the placenta. These defects compromise embryonic development and intrauterine viability only after the establishment of placental circulation. For example, the lack of chorioallantoic branching does not affect embryonic development or intrauterine viability until E10.5, as evidenced by *Gcm1* knockout embryos, which develop normally until E9.5 (Anson-Cartwright et al., 2000; Schreiber et al., 2000). *Tfeb* deletion also results in normal embryonic development at E9.5 (Steingrímsson et al., 1998). By E10.5, cell death is apparent in KO embryos, which become unrecognizable by E11.5. To better assess the function of this complement of genes, ISH was performed with WT tissues at relevant stages with gene-specific probes. Most are widely expressed in the embryo and unexpectedly confined to the trophoblast compartment of the placenta, indicating that their lethal KO phenotype could be due to an essential role in either the embryo or the trophoblast compartment. The exception is *Exoc3l4*, which is not embryonically expressed but is highly expressed in the placenta, suggesting that the essential role of *Exoc3l4* is in the placenta, where its loss drives the observed embryonic defects. *Exoc3l4*^−/−^ mutants are generally much smaller than their littermates at E10.5 and are present but dying at E11.5, a phenotype shared by embryos that are lacking crucial components of the LZ (Anson-Cartwright et al., 2000; Schreiber et al., 2000; Steingrímsson et al., 1998). The enriched expression of *Exoc3l4* in the S-TGC population (Fig. S7) suggests that it is S-TGC specific and that the KO may impact S-TGCs at the earliest stage.

The streamlined phenotyping pipeline used herein identified specific placental defects that coincide with mid-gestation embryo phenotypic onset in most KO lines examined; however, it is not without limitations. The analysis pipeline did not molecularly assess the YS and it is possible that YS or trophectoderm-specific defects contribute to embryonic demise. Similarly, the pipeline does not directly examine maternal spiral artery remodeling or the TGC subtypes that line the channels and deliver maternal blood into the labyrinth. In our approach, their differentiation is indirectly inferred by the presence of maternal blood in the trophoblast compartment. Nonetheless, the pipeline provides a wealth of information using cell-type specific analyses, making it well suited for identifying many aspects of the mid-gestation placenta.

Metadata analysis of well-studied genes reveals a strong correlation between the level of research attention a gene receives and the availability of an existing knockout model (Stoeger et al., 2018). The concentration on well-studied genes, rather than exploring understudied genes, not only limits the drug discovery pipeline but also our understanding of the genetic underpinnings of human health and disease. With approximately 1/33 babies born with congenital anomalies, large-scale exome sequencing efforts are underway at many major medical centers to identify potential genetic causes of these anomalies. While these technologies uncover numerous candidates, the lack of functional annotation for understudied genes can result in the classification as a variant of unknown significance (Richardson et al., 2024). The work presented here contributes to understanding how these 22 understudied genes function during murine development. Given the coincidence of embryonic and placental phenotype onset and their co-expression in both tissue-types, a next step in understanding their mammalian function is determining if these genes are crucial in the embryo or in the placenta.

## MATERIALS AND METHODS

### Mouse strains

Sex as a biological variable was not considered because the embryonic stages used occur before the onset of murine sex differentiation (end of E10.5). All animal studies are approved by the University of Massachusetts Amherst's Institutional Animal Care and Use Committee (IACUC protocol #3498 and #6603). KO alleles were generated by the Knockout Mouse Program (KOMP2) production centers. All KO lines obtained were produced in and maintained on a C57BL/6NJ background. KO alleles are each produced by endonuclease-mediated indel (‘em1’) mutations in a crucial exon of each gene, which are intended to result in a frameshift mutation leading to nonsense-mediated decay or early protein truncation (Elrick et al., 2024). Each heterozygous line was obtained live from one of the production centers that had already determined that KO embryos were present at E9.5 but absent at E12.5 and that this lethality was fully penetrant. Only lines for which a KO allele had not yet been published were selected. Additional strain information for each KO line can be found in Table S1 and by searching the IMPC site for the strain name.

### Embryo and placenta collection

KO embryos were generated from heterozygous intercrosses and the morning a copulatory plug was observed marked E0.5 of gestation. Genotypes were determined by PCR on ear clips or YS, using primers either provided by the production centers or designed in-house to optimize fidelity (Table S3). To examine the WT expression of each KO gene, embryos and placentas were collected from CD-1 animals (Charles River, bred in house). Placentas were fixed in 4% paraformaldehyde (PFA) overnight at 4°C whereas embryos were fixed in 4% PFA overnight at 4°C or for 1 h at room temperature. Placentas and embryos were then rinsed in DEPC-treated phosphate buffered saline with 1% Tween-20 (PBT) and dehydrated in an increasing methanol series in DEPC-treated PBT. Placentas were cleared in xylene and paraffin and embedded for sagittal sectioning (7 μm). For analysis of KO placentas, sets of five adjacent sections from the midline, identified by the allantoic attachment point/umbilical cord, were placed onto different slides for H&E, ISH or immunofluorescence.

### Embryonic phenotyping and analysis

To keep the embryo, extra-embryonic tissues and placenta intact, the decidua surrounding the embryonic portion of the embryo as well as adherent TGCs/parietal endoderm was carefully removed to reveal the YS surrounding the embryo. The condition of intact YS was noted. Upon removing the YS, the YS attachment to the placenta is preserved. After YS removal, the status of the allantois attachment to the chorion/placenta is made more apparent and noted. Finally, the placental attachment (allantois/umbilicus) to the embryo is carefully severed to avoid disturbing the placental attachments. After dissection, each intact embryo was assessed for phenotypic defects and photographed on a Nikon SMZ1500 dissecting microscope with a SPOT model 29.2-1.3 MP color camera and SPOT 5.6 software and dissection notes recorded. Because of the natural variation of embryo development and because ‘delay’ is relative to such variation, developmental delay was determined relative to the entire litter. A large array of embryonic phenotypes were assessed using mammalian phenotype (MP) terms and criteria (https://www.informatics.jax.org/vocab/mp_ontology); however, only those that were consistently found in more than one line have been included in the phenotyping rubrics presented herein. At each embryonic day at least ten mutant embryos were examined (except as noted) from a minimum of five litters, and all embryos were systematically phenotyped and scored. Embryonic delay, damage due to dissection or other confounding issues could prevent the scoring of a particular phenotype, and phenotypes were not scored if the number of applicable mutants was fewer than 7. A *P*-value≤1×10^−4^, obtained using a two-tailed Fisher's Exact test with mutant and control values for each phenotype, was used as a threshold for significance. Additional phenotype information is available at https://websites.umass.edu/kdtkomp/.

### *In situ* hybridization probe production

*Tpbpa* (Adelman et al., 2000), *Tfeb* and *Gcm1* probes were a gift (Celeste Simon, University of Pennsylvania, PA, USA). Sequencing demonstrated that the *Tfeb* probe corresponds to an ∼400 bp region towards the 5′ end of the exon coding region (NM_011549.4) and that the *Gcm1* probe recognizes almost the entire coding region (NM_008103.3). The *Pl-1* probe was also a gift (Janet Rossant, University of Toronto, Canada)*.* Probes that correspond to the WT gene altered in each KO line were generated as previously described (Guertin et al., 2022). The primer sets used to generate the cloned probe from cDNA are listed in Table S4. An *Ehbp1* probe was never successfully generated, and the primers used to generate the *Zmym2* probe have been previously published (Gray et al., 2004).

### *In situ* hybridization

Whole mount *in situ* hybridization (WISH) on embryos was performed as previously described (Archambault et al., 2020) and embryos were imaged using a Nikon SMZ1500 dissecting microscope with a SPOT model 29.2-1.3 MP color camera and SPOT 5.6 software.

Section *in situ* hybridization (SISH) on placentas was performed as follows. Slides were dewaxed in xylene and rehydrated in ethanol. Slides were rinsed in PBS before being fixed for 15 min at room temperature in 4% PFA. After a PBS rinse, slides were treated with 15 μg/ml of Proteinase K (Sigma-Aldrich, P2308) in PBS for 5 min followed by 5 min of 2 mg/ml glycine in PBS. Slides were rinsed in PBS and fixed again in 4% PFA for 15 min. Slides were acetylated for 10 min with a freshly emulsified solution of 0.25% acetic anhydride with 0.1 M triethanolamine in PBS. Slides were rinsed in PBS and then incubated for at least 1 h at 65°C in hybridization buffer containing 50% deionized formamide (Millipore, S4117), 5× SSC, 4% SDS (Sigma-Aldrich, 71725), 50 μg/ml heparin (Sigma-Aldrich, H3393) and 50 μg/ml brewer's yeast tRNA (Thermo Fisher Scientific, 15401029). Hybridization was carried out overnight at 70°C with the probes of interest in hybridization buffer. The next day, slides were washed three times for 15 min each at 65°C in solutions of 50% formamide (Thermo Fisher Scientific, F84-1), 5× SSC and 1% SDS. Slides were then washed three times for 15 min each at 65°C in solutions of 50% formamide and 2× SSC. Slides were allowed to reach room temperature and then washed three times with maleic acid buffer/0.1% Tween-20 (MABT). Blocking was performed for 30 min in a solution of MAB with 2% Boehringer blocking reagent (BBR; Roche, 11096176001), 5% heat inactivated sheep serum (Sigma-Aldrich, S2263) and 1% Tween-20. Slides were then incubated for 2 h in MAB with anti-digoxigenin-AP (Roche, 11093274910, 1:5000), 2% BBR and 1% heat inactivated sheep serum. Slides were washed four times in MABT buffer followed by three washes in alkaline phosphatase buffer (NTMT) composed of 0.1 M NaCl, 0.1 M Tris-base (pH 9.5), 0.05 M MgCl_2_ and 1% Tween-20. Slides were incubated with BM purple (Roche, 11442074001) until sufficient color had developed. The reaction was stopped in a PBS wash with 1 mM EDTA. Slides were then dehydrated in ethanol, counterstained with Eosin Y (Polysciences, Inc., 09859) for 20 s, rinsed twice in 100% ethanol for 10 min and washed in xylene for 10 min. Slides were cover-slipped with Cytoseal60 (Electron Microscopy Services) and imaged on a Nikon Eclipse Ti2-S-U inverted microscope with a Nikon DS-Ri2 camera and NIS Elements imaging software or on a Panoramic MIDI slide scanner (3DHistech). A 20× objective was used on the Nikon microscope. To capture the entire placenta, multiple 20× images were acquired and stitched together in NIS elements. Due to the nature of colorimetric staining, signal intensity and Eosin counterstaining can vary between experiments. However, all KO and control littermate pairs were processed side by side, from dissection through staining to imaging, and are therefore directly comparable with each other.

### Hematoxylin and Eosin staining

H&E staining of placenta samples was performed as previously described (Guertin et al., 2022). Slides were imaged on a Nikon Eclipse Ti2-S-U inverted microscope with a Nikon DS-Ri2 camera and NIS Elements imaging software or on a Panoramic MIDI slide scanner (3DHistech).

### Immunofluorescence

Slides were deparaffinized and rehydrated before antigen retrieval in boiling citric acid buffer (pH 6). After cooling to room temperature, slides were blocked in 1% bovine serum albumin (BSA)/PBT for 2 h at room temperature. Primary antibodies were prepared in 1% BSA/PBT and incubated on slides in a humidified chamber overnight at 4°C. The primary antibodies and working dilutions used are as follows: rabbit anti-MCT4 (Millipore, AB3314P, 1:500), chicken anti-MCT1 (Sigma-Aldrich, AB1286-I, 1:500), and goat anti-Endomucin (R&D Systems, AF4666, 1:400). Slides were then incubated with secondary antibody solutions prepared in 1% BSA in PBT for 1 h at room temperature. The secondary antibodies used were Alexa Fluor 488, 546 and 647 donkey antibodies (Molecular Probes, A78948, A11056, A31373, 1:500). Slides were counterstained with DAPI (Roche, 1:10,0000 in PBS) and cover slipped with ProLong™ Gold (Invitrogen). Slides were imaged using a Nikon Eclipse Ti2-S-U inverted microscope with a Photometrics Iris 15™ sCMOS camera and NIS Elements imaging software.

### Placenta phenotyping and analysis

For each KO line, at least three mutant and control placentas from conceptuses with stage-matched embryos were analyzed. Controls were typically heterozygous littermates and for most KO lines, pairs of KO and controls from at least two litters were examined. Placental defects were identified in KO placentas after direct comparison to control littermates. A KO line was considered to exhibit a particular phenotype (Fig. 4, see figure legend for assessment criteria) if the abnormality was observed in all placentas analyzed (minimum of *n*=3) unless otherwise stated. All data were assessed by two independent individuals.

### Published mouse placenta RNA-seq datasets use

Publicly available single-nucleus RNA-seq data from Marsh and Blelloch (2020) and single-cell RNA-seq from Jiang et al. (2023) were re-analyzed using the processed R object, or by replicating the authors’ analysis pipeline available on GitHub, respectively. All downstream visualizations, including dot plots and feature plots, were performed using R package Seurat v4 (Hao et al., 2021).

### Gene-disease association

Human disease associations were collected from the OMIM and Orphanet (v 1.0.19) databases. Non-Mendelian inherited disorders and diseases in which the gene of interest was not the sole loci involved were not included. Human phenotype ontology annotations were downloaded (https://hpo.jax.org/data/annotations) for each gene with an associated Mendelian-inherited disorder. Redundant annotations were excluded, and specific phenotype abnormality annotations were mapped back to one of the 23 broader parent terms. The ratio of annotations for each parent term to total annotations was calculated for each annotated disorder. Mode of inheritance was determined from HPO annotations, and clinical onset was specified using HPO terms or annotations from the OMIM/Orphanet databases.

## Supplementary Material



10.1242/develop.205276_sup1 Supplementary information

Table S3. Genotyping primers for the 22 KO lines. PCR product sizes for each reaction are listed in parentheses.
