# Supplementary material for: Onset of embryonic and placental defects coincide in 19 of 22 novel mid-gestation lethal murine knockout lines
Source: Development. 2026 Jun 2;153(10):dev205276. doi: 10.1242/dev.205276 (PMC13286363; doi:10.1242/dev.205276)
Supplement: Supplementary information [file develop-153-205276-s1.pdf]

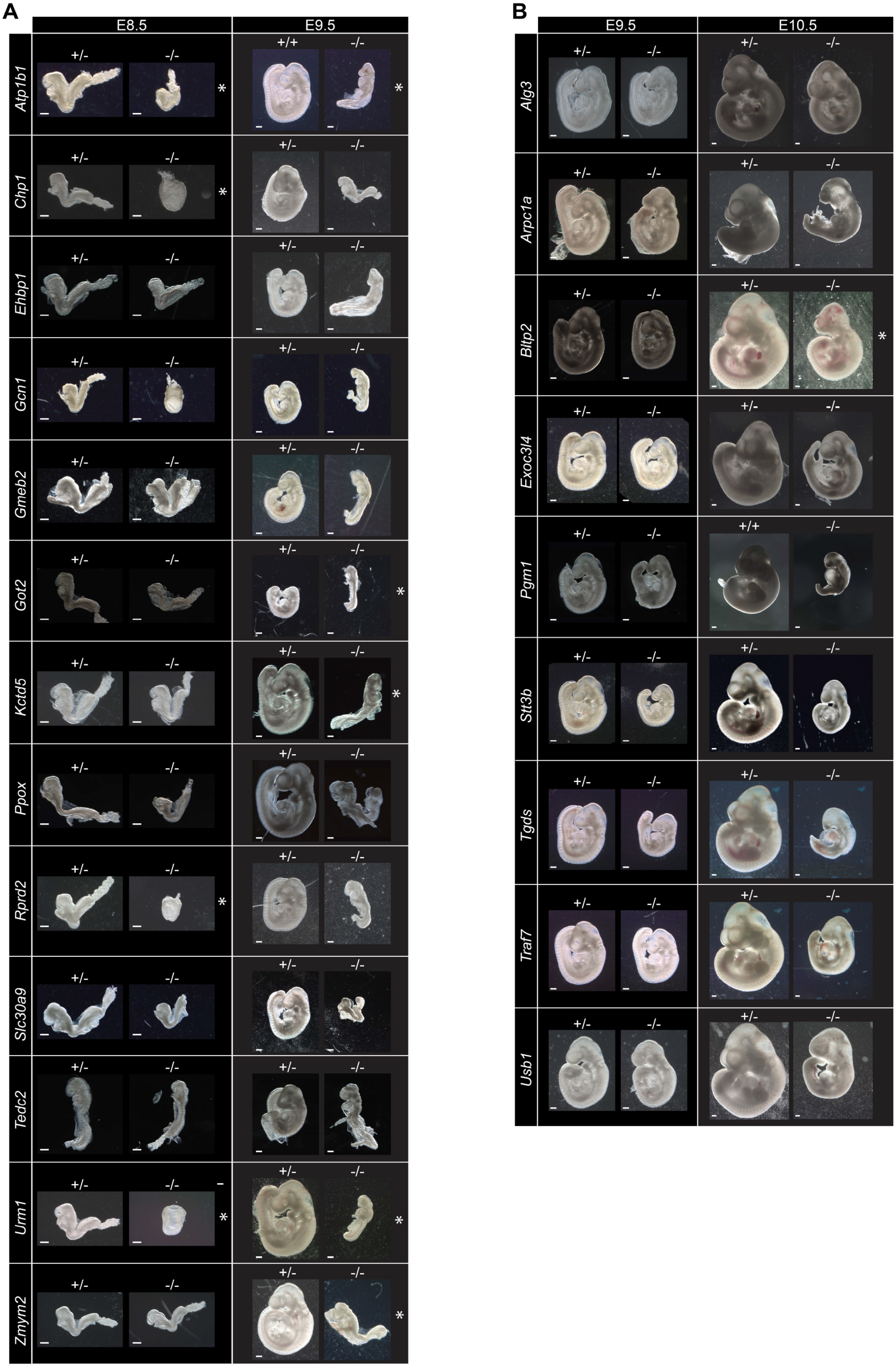

Development • Supplementary information

Fig. S1. Representative null KO embryos for all 22 KO lines. (A) Representative control (+/-) and null littermate at E8.5 and 9.5 from each of the 13 “E9.5 onset” KO lines as indicated. (B) Representative control (+/-) and null littermate at E9.5 and 10.5 from all 9 of the “E10.5 onset” lines as indicated. Please note that some of these images are the same as used in Fig. 1. *Indicates some KO embryos have less severe phenotype. Scale bars: 250 µm.

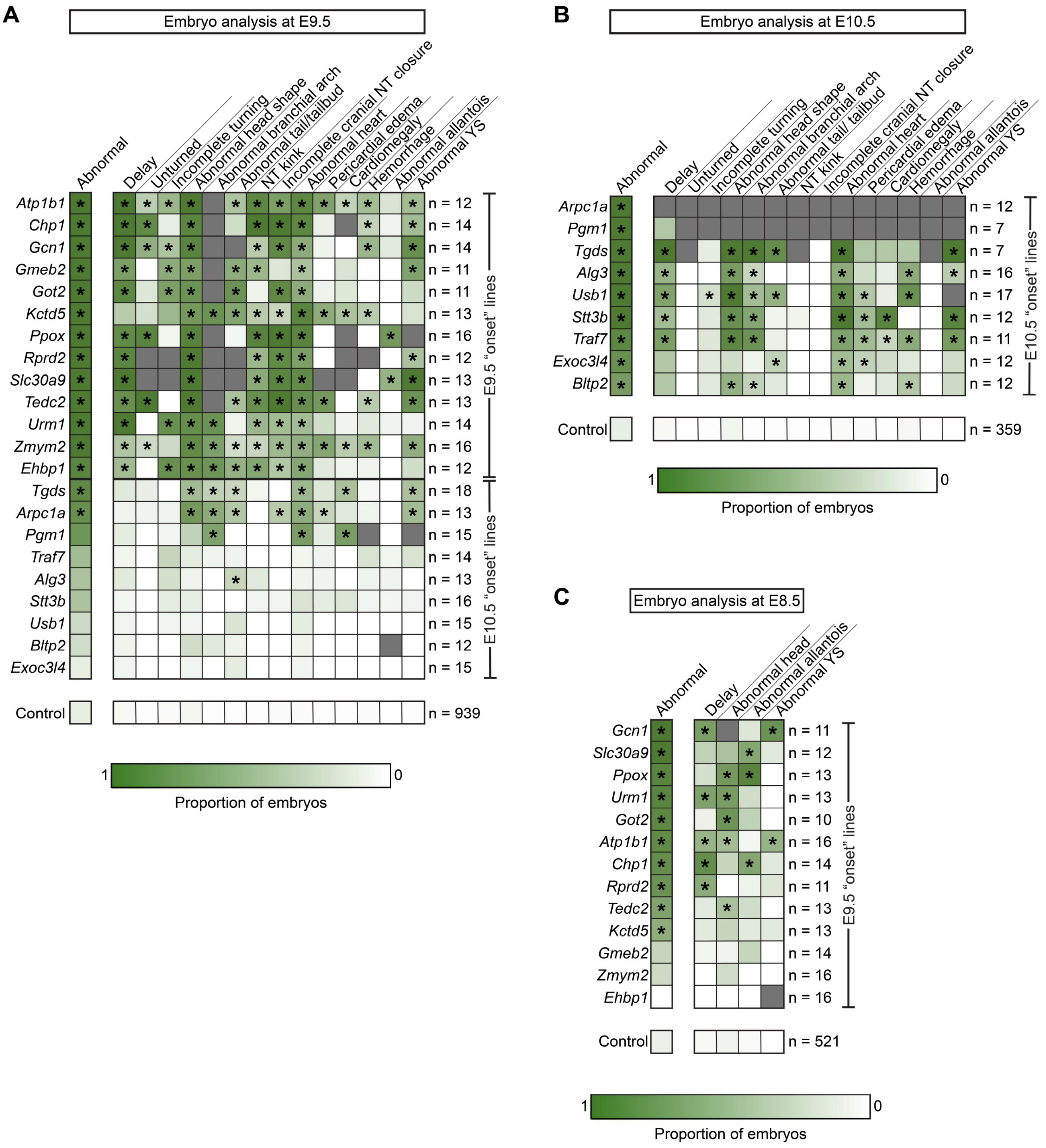

Development • Supplementary information

Fig. S2. Summary of null embryonic defects observed in 22 KO lines. (A-C) Heatmap representing the proportion of embryos that display defects in each phenotype category, representing Mammalian Phenotyping (MP) terms listed on MGI. The KO lines are listed (top to bottom) by those with the highest proportion of abnormal KO embryos to those with the lowest proportion. A grey box indicates that the phenotype was not scored. The total number of embryos examined in each column is provided. The control group includes all non-mutant littermates. An asterisk is placed on any category with a p-value < 0.001 using a 2-tailed Fishers exact test. (A) Phenotype analysis of mutant embryos from all KO lines at E9.5. (B) Phenotype analysis of E10.5 embryos from the "E10.5 onset" lines. *Arpc1a* $^{-/-}$, *Pgm1*$^{-/-}$ and *Tgds*$^{-/-}$ embryos undergo a precipitous decline between E9.5 and 10.5 often resulting in too few analyzable embryos to reliably phenotype. (C) A modified phenotyping scheme was used to examine "E9.5 onset" lines at E8.5.

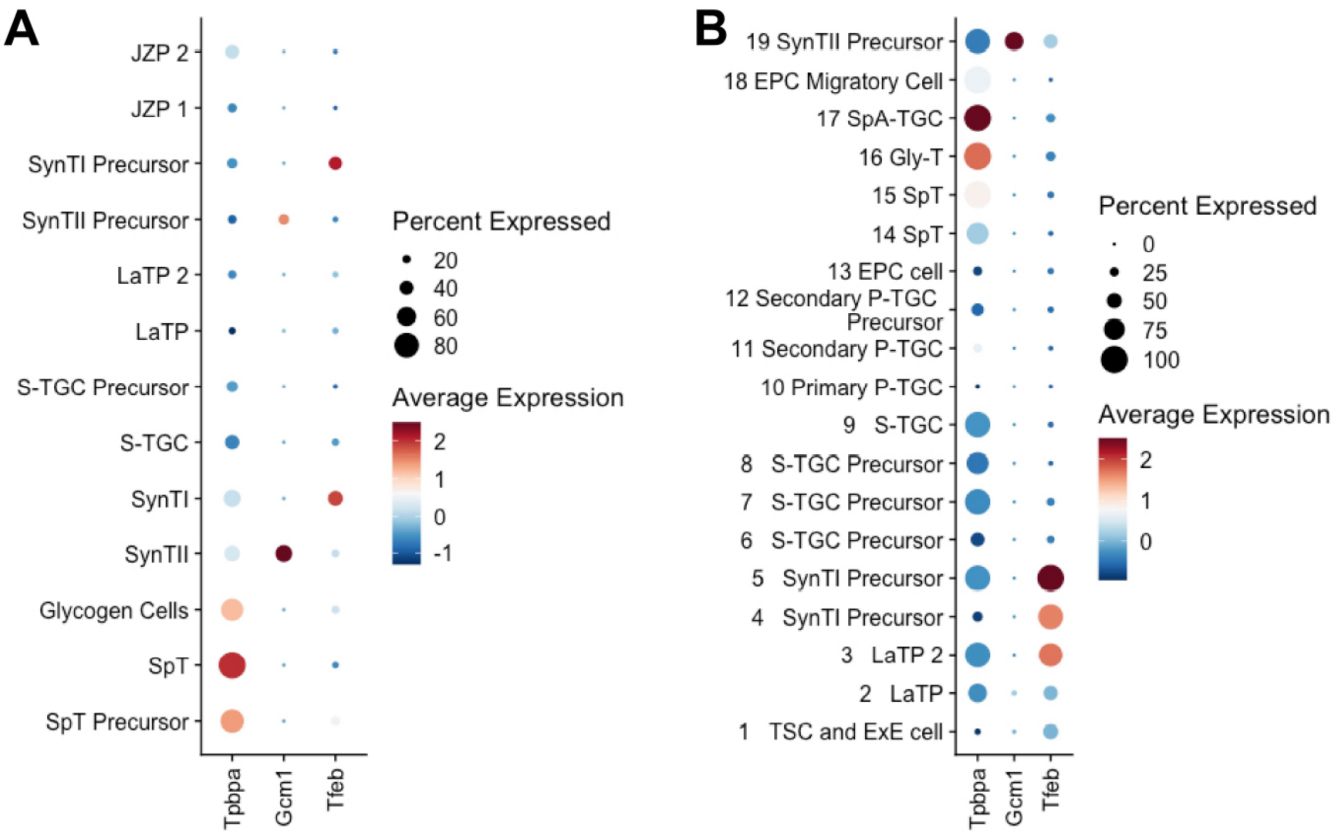

Fig. S3. Expression of *Tpbpa*, *Tfeb*, and *Gcm1* in trophoblast clusters from published RNA-seq datasets. (A) Dot plot showing relative expression and percentage of nuclei expressing each of the placental markers used for *in situ* hybridization analysis across the 13 trophoblast clusters from Marsh and Blelloch (2020). *Tpbpa* is enriched in the SpT layer and to a lesser extent the SpT precursors and glycogen cells. *Gcm1* is specific to the SynTII cells and their precursors while *Tfeb* is specific to the SynTI cells and their precursors. (B) Dot plot showing relative expression and percentage of expressing cells in each trophoblast cluster from Jiang et al. (2023) for the placenta markers used in ISH analysis. *Tpbpa* is enriched in the glycogen trophoblasts and SpA-TGCs. *Gcm1* is specific to SynTII precursors. *Tfeb* is enriched in the LaTP2 population and SynTI precursors.

Development • Supplementary information

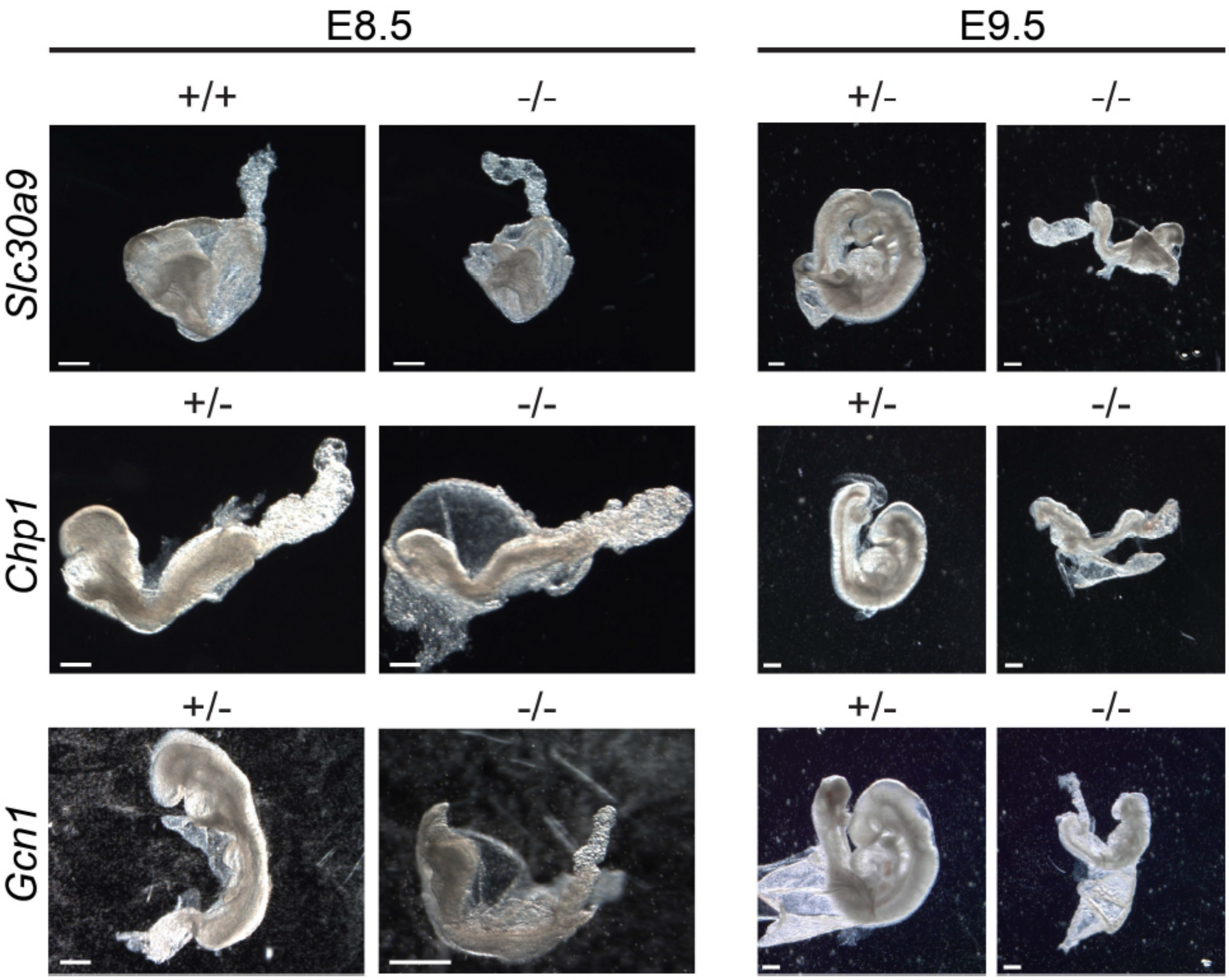

Fig. S4. Allantois extension is observed in KO conceptuses with impaired chorioallantoic attachment. At E8.5 and E9.5, allantois extension is observed from the posterior of *Slc30a9*$^{-/-}$, *Chp1*$^{-/-}$, and *Gcn1*$^{-/-}$ embryos. Scale bars: 250 µm.

Development • Supplementary information

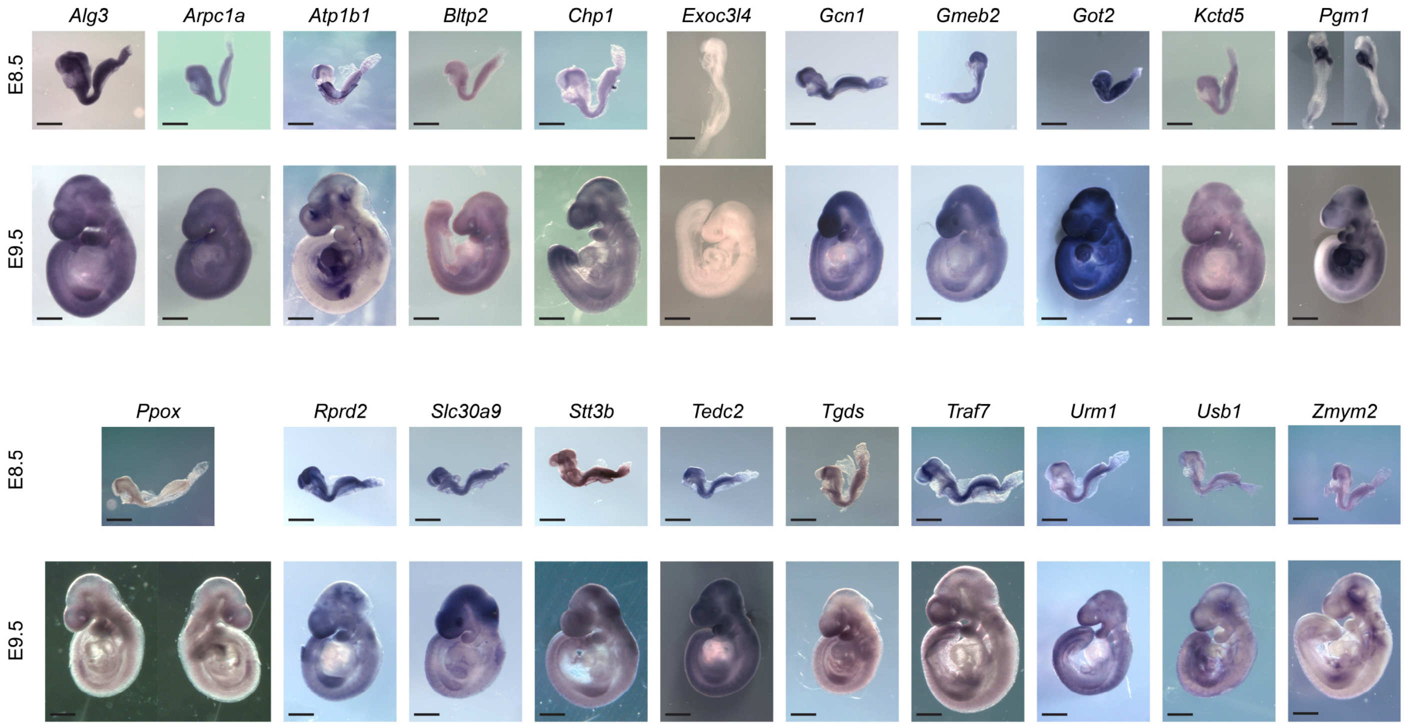

Fig. S5. Endogenous expression of each gene in E8.5 and E9.5 embryonic tissues. Whole mount *in situ* hybridization images showing the endogenous expression pattern of each gene in WT embryos at E8.5 and E9.5. At E8.5, the allantois is present; however, gene expression was rarely observed throughout its length and was typically restricted to the base where it connects to the posterior of the embryo. The expression patterns gathered here largely mirrored those previously reported for six genes with published whole embryo mid-gestation expression [*Pgm1* and *Tgds*: (Tamplin et al., 2008); *Arpc1a* and *Atp1b1*: (Magdaleno et al., 2006); *Kctd5* and *Zmym2*: (Gray et al., 2004)]. However, *Kctd5* expression appeared more robust and widespread in the E9.5 embryo compared to the reported restriction to the limb buds and pharyngeal arch at E10.5 (Gray et al., 2004). This unexpected difference may reflect variation in the duration of color development.

Development • Supplementary information

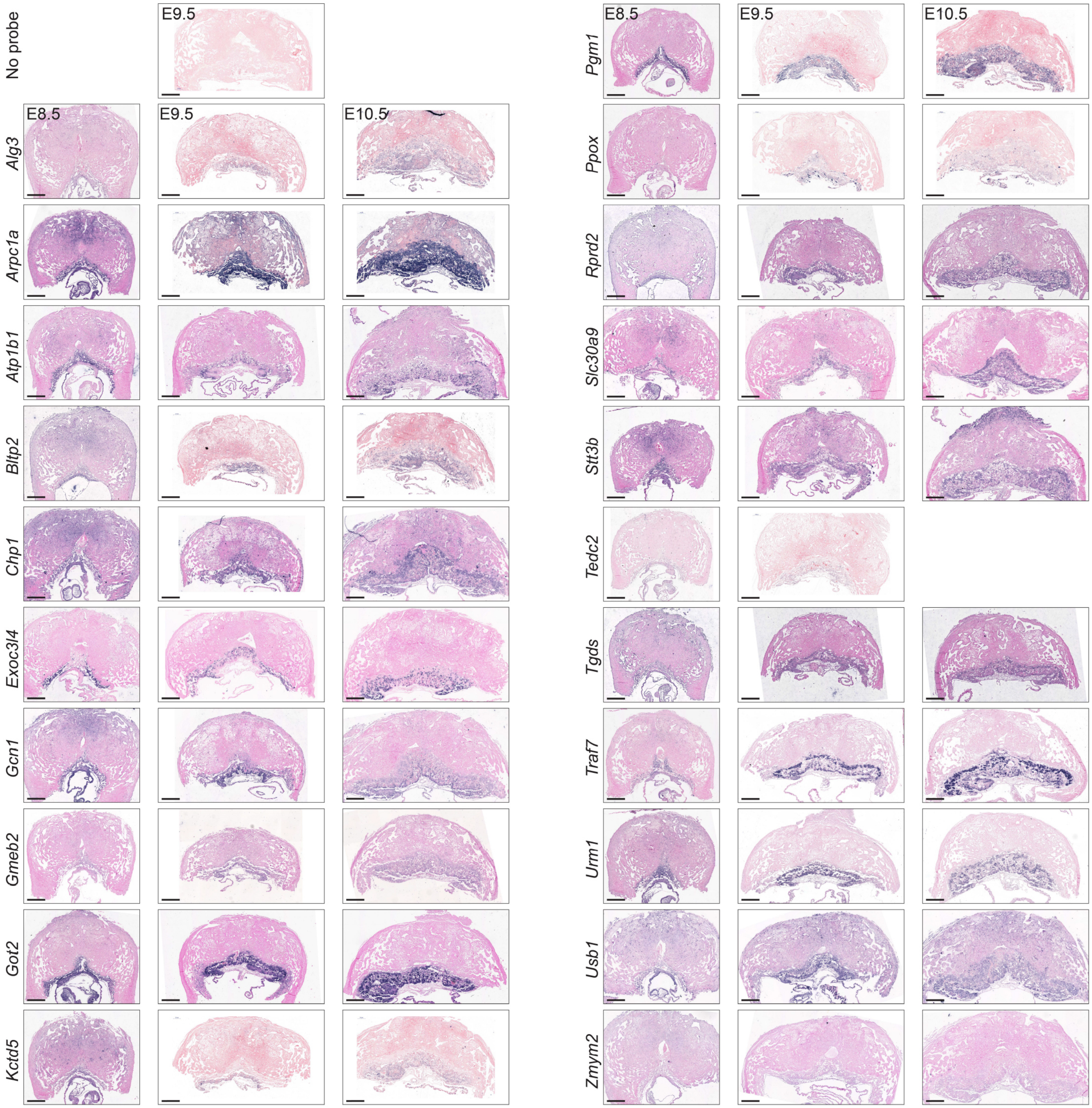

Fig. S6. Endogenous expression of each gene in the E8.5-E10.5 placenta. Sectioned *in situ* hybridization images showing the endogenous expression pattern of each gene in the placenta at E8.5, 9.5, and 10.5. The E9.5 *Arpc1*, *Exoc3l4* and *Pgm1* images are used in Fig. 8.

Development • Supplementary information

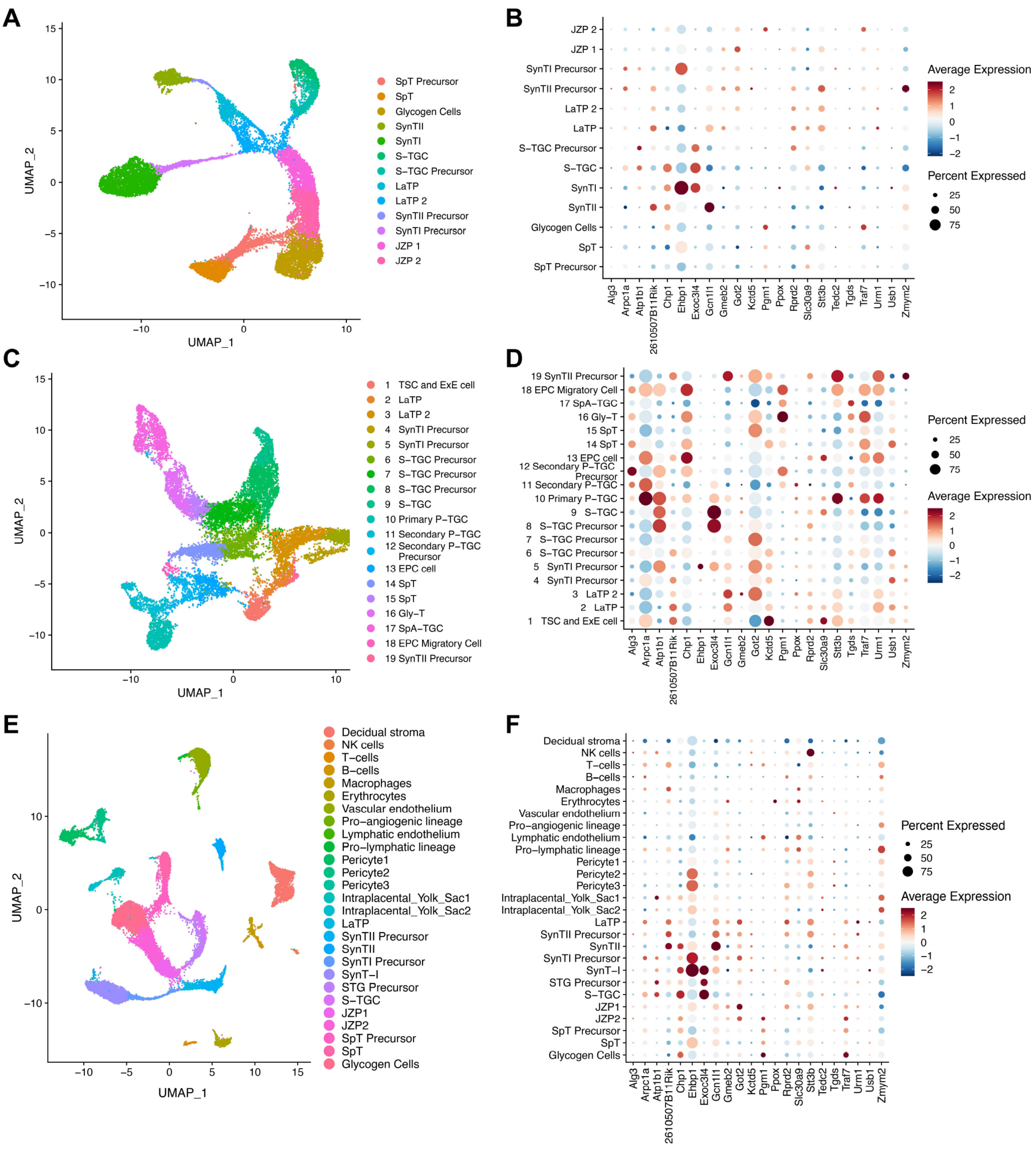

A
UMAP_1
UMAP_2
SpT Precursor
SpT
Glycogen Cells
SynTII
SynTI
S−TGC
S−TGC Precursor
LaTP
LaTP 2
SynTII Precursor
SynTI Precursor
JZP 1
JZP 2
B
Average Expression
Percent Expressed
Alg3
Arpc1a
Atp1b1
2610507B11Rik
Chp1
Ehbp1
Exoc3l4
Gcn1l1
Gmeb2
Got2
Kctd5
Pgm1
Ppox
Rprd2
Slc30a9
Stt3b
Tedc2
Tgds
Traf7
Urm1
Usb1
Zmym2
C
1 TSC and ExE cell
2 LaTP
3 LaTP 2
4 SynTI Precursor
5 SynTI Precursor
6 S−TGC Precursor
7 S−TGC Precursor
8 S−TGC Precursor
9 S−TGC
10 Primary P−TGC
11 Secondary P−TGC
12 Secondary P−TGC Precursor
13 EPC cell
14 SpT
15 SpT
16 Gly−T
17 SpA−TGC
18 EPC Migratory Cell
19 SynTII Precursor
D
E
Decidual stroma
NK cells
T−cells
B−cells
Macrophages
Erythrocytes
Vascular endothelium
Pro−angiogenic lineage
Lymphatic endothelium
Pro−lymphatic lineage
Pericyte1
Pericyte2
Pericyte3
Intraplacental_Yolk_Sac1
Intraplacental_Yolk_Sac2
LaTP
SynTII Precursor
SynTII
SynTI Precursor
SynT−I
STG Precursor
S−TGC
JZP1
JZP2
SpT Precursor
SpT
Glycogen Cells
F

Fig. S7. Expression of mid-gestation lethal genes in trophoblast clusters from published RNA-seq datasets.

(A) UMAP projection of trophoblast nuclei isolated from E9.5, E10.5, E12.5, and E14.5 placentas revealing the 13 distinct cell clusters (Marsh and Blelloch, 2020). Two labyrinth trophoblast progenitor populations (LaTP and LaTP2) give rise to the three trophoblast lineages of the interhemal barrier; S-TGC, SynTI, and SynTII. Two JZ progenitor populations (JZP 1, and JZP 2) give rise to the SpT and glycogen cell lineages. (B) Dot plot showing relative expression and percentage of nuclei expressing each of the 22 mid-gestation lethal genes across the 13 trophoblast clusters from Marsh and Blelloch (2020). *Bltp2* and *Gcn1* were identified in the dataset under their alternative names/ synonyms, 2610507B11Rik and Gcn1l1, respectively. (C) UMAP projection of trophoblast cells isolated from E7.5, E8.5, E9.5, E10.5, E11.5, E12.5, E13.5, and E14.5 extraembryonic tissues/ placentas revealing 19 distinct clusters (Jiang et al., 2023). E7.5-E8.5 cells predominately give rise to the trophoblast stem cell (TSC) and extraembryonic (ExE) cell cluster, ectoplacental cone (EPC) cell cluster, EPC migratory cell cluster, primary trophoblast giant cells (TGCs), secondary parietal TGC (P-TGC) precursors, and mature P-TGC. E9.5-E14.5 trophoblast cells rise to two labyrinth trophoblast progenitor populations (LaTP), two SynTI precursor clusters, 3 S-TGC precursors, mature S-TGCs, SynTII precursors, two spongiotrophoblast (SpT) clusters, glycogen trophoblasts (GlyT), and spiral artery-associated TGCs (SpA-TGC). (D) Dot plot showing relative expression and percentage of expressing cells in each trophoblast cluster from Jiang et al. (2023) for 21 of the mid-gestation lethal genes. *Tedc2*, nor any of its alternative names/ synonyms were found in the dataset, likely due to low expression. *Bltp2* and *Gcn1* were identified in the dataset under their alternative names/ synonyms, 2610507B11Rik and Gcn1l1, respectively. (E) UMAP projection of all nuclei isolated from E9.5, E10.5, E12.5, and E14.5 placentas revealing 27 cell clusters (Marsh and Blelloch, 2020). (F) Dot plot showing relative expression and percentage of nuclei expressing each of the 22 mid-gestation lethal genes across all cell clusters from Marsh and Blelloch (2020). *Bltp2* and *Gcn1* were identified in the dataset under their alternative names/ synonyms, 2610507B11Rik and Gcn1l1, respectively.

Development • Supplementary information

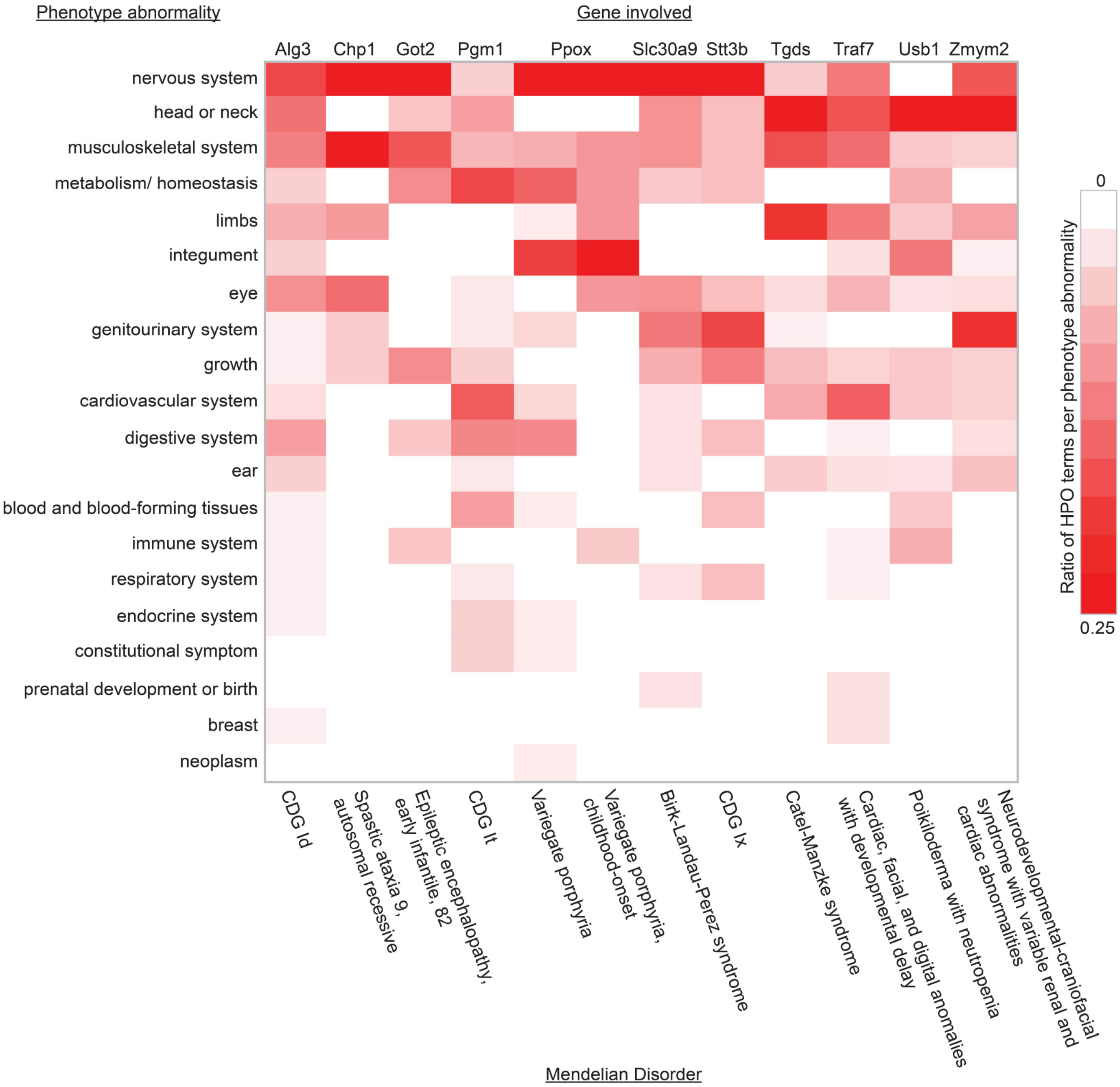

Fig. S8. Many mid-gestation lethal genes are associated with a human Mendelian disorder. Heatmap displaying the distribution of Human Phenotype Ontology (HPO) annotations across parent phenotype abnormality categories, normalized to the total number of annotations per disorder. Parent phenotype categories are listed on the left. Associated Mendelian disorders are shown at the bottom, with the corresponding gene involved are listed above.

Development • Supplementary information

Table S1. Gene and strain information for the 22 KO lines.

Gene symbol (mouse)	Gene Name	Human Ortholog	MMRRC Strain Number
Alg3	ALG3 alpha-1,3- mannosyltransferase	ALG3	046096-JAX
Arpc1a	actin related protein 2/3 complex, subunit 1A	APRC1A	046238-JAX
Atp1b1	ATPase, Na+/K+ transporting, beta 1 polypeptide	ATP1B1	067430-UCD
Bltp2	bridge-like lipid transfer protein family member 2	BLTP2	046131-JAX
Chp1	calcineurin-like EF hand protein 1	CHP1	068049-UCD
Ehbp1	EH domain binding protein 1	EHBP1	051252-JAX
Exoc3l4	exocyst complex component 3-like 4	EXOC3L4	051121-JAX
Gcn1	GCN1 activator of EIF2AK4	GCN1	066826-JAX
Gmeb2	glucocorticoid modulatory element binding protein 2	GMEB2	068243-JAX
Got2	glutamatic-oxaloacetic transaminase 2, mitochondrial	GOT2	065485-UCD
Kctd5	potassium channel tetramerisation domain containing 5	KCTD5	051084-JAX
Pgm1	phosphoglucomutase 1	PGM1	043780-JAX
Ppox	protoporphyrinogen oxidase	PPOX	051148-JAX
Rprd2	regulation of nuclear pre-mRNA domain containing 2	RPRD2	046086-JAX
Slc30a9	solute carrier family 30 (zinc transporter), member 9	SLC30A9	071118-UNC
Stt3b	STT3, subunit of the oligosaccharyltransferase complex, homolog B (S. cerevisiae)	STT3B	046074-JAX
Tedc2	tubulin epsilon and delta complex 2	TEDC2	043782-JAX
Tgds	TDP-glucose 4,6-dehydratase	TGDS	067340-UCD
Traf7	TNF receptor-associated factor 7	TRAF7	068046-UCD
Urm1	ubiquitin related modifier 1	URM1	065750-JAX
Usb1	U6 snRNA biogenesis 1	USB1	068413-MU
Zmym2	zinc finger, MYM-type 2	ZMYM2	051239-JAX

Table S2. Human disorders associated with genes from KO line examined herein.

Human ortholog	Disorder	Inheritance	Phenotype MIM #/ *Orphacode*	Onset (earliest)
ALG3	Congenital disorder of glycosylation, type Id	AR	601110/ *79321*	Congenital
ATP1B1	Essential hypertension	*	145500	
CHP1	Spastic ataxia 9, autosomal recessive	AR	618438	Childhood
EHBP1	Prostate cancer, hereditary, 12	-	618438	-
GOT2	Developmental and epileptic encephalopathy 82	AR	618721	Neonatal
PGM1	Congenital disorder of glycosylation, type It	AR	614921/ *319646*	Neonatal
PPOX	Variegate porphyria<br>Variegate porphyria, childhood-onset	AD<br>AR	176200/ *79473*<br>620483	Young adult<br>Neonatal
SLC30A9	Birk-Landau-Perez syndrome	AR	617595/ *505242*	Fetal
STT3B	Congenital disorder of glycosylation, type Ix	AR	615597/ *370924*	Fetal
TGDS	Catel-Manzke syndrome	AR	616145/ *1388*	Congenital
TRAF7	Cardiac, facial, and digital anomalies with developmental delay<br>Meningioma	AD<br>**	618164/ *592570*<br>*2495*	Congenital<br>-
USB1	Poikiloderma with neutropenia<br>Dyskeratosis congenita	AR<br>**	604173<br>*1775*	Neonatal
ZMYM2	Neurodevelopmental-craniofacial syndrome with variable renal and cardiac abnormalities<br>Non-specific syndromic intellectual disability	AD<br>**	619522<br>*528084*	Neonatal<br>Infantile

-, Information not available in OMIM or Orphanet databases.
*, Non-Mendelian inheritance.
**, Variants in other genes are also independently associated with the same disorder.

Table S3. Genotyping primers for the 22 KO lines. PCR product sizes for each reaction are listed in parentheses.

Allele	Primer	Sequence (5' to 3')	Source
Alg3 wild type	Forward	CCT TGG TGC TTG TTT TCT TGA	JAX
(208 bp)	Reverse	GAG CCG CAG TAC AAA GAT GG	*
Alg3 knockout	Forward	GGA GAT AGC TTA GCA CGT GG	*
(549 bp)	Reverse	TCT CCT GCA GAA CAT TGG GA	*
Arpc1a wild type	Forward	AGA AGG AGG GGA AGAAACCA	JAX
(108 bp)	Reverse	AGC ACA GGT CAC TTG CTA CG	JAX
Arpc1a knockout	Forward	AGA AGG AGG GGA AGA AAC CA	JAX
(93 bp)	Reverse	GGA GTA AGG AAG GGA AGT GAA GA	JAX
Atp1b1 wild type	Forward	CTT TCA CTG CAA GTG ACA TTC TCG	UCD
(459 bp)	Reverse	CCA GTC ACT TAC TGC CAC AGT CCT C	UCD
Atp1b1 knockout	Forward	CTT TCA CTG CAA GTG ACA TTC TCG	UCD
(450 bp)	Reverse	GCA GAG ACC GTG GTA GGT TCT AGG T	UCD
Bltp2 wild type	Forward	GAG CTA CTT GGA GGA CTC TGC	JAX
(101 bp)	Reverse	CGG CCT CTT GAG AAA CAA G	JAX
Bltp2 knockout	Forward	AAA TCC GGC TTC AGG ATA CC	JAX
(102 bp)	Reverse	CGG CCT CTT GAG AAA CAA G	JAX
Chp1 wild type	Forward	GCC CTG GTT GGA CTA TTG CC	*
(416 bp)	Reverse	GCC CTG GTT GGA CTA TTG CC	*
Chp1 knockout	Forward	GTA CTG ACT TTC ATG TGT GGT GC	TCP
(808 bp)	Reverse	CCA CTG AGC TCA AAC CTT CTA GTA TC	TCP
Ehbp1 wild type	Forward	TCT CCA GTT CTC ACT GTC CTG	JAX
(149 bp)	Reverse	GCA TTC ATC CTC TGA AAA CA	JAX
Ehbp1 knockout	Forward	AGT GCT GGA CTT ATA GGT GTG AG	JAX
(147 bp)	Reverse	GCA TTC ATC CTC TGA AAA CA	JAX
Exoc3l4 wild type	Forward	GAG AGG AGG TAG CTG GTG GA	JAX
(393 bp)	Reverse	GCC TGT GGT GAG AAG CTT TC	*
Exoc3l4 knockout	Forward	GAG AGG AGG TAG CTG GTG GA	JAX
(218 bp)	Reverse	AGG GAT GCC ACA TGT TGA GA	*
Gcn1 wild type	Forward	GAG GCA CTG GAT GGG CAA A	*
(491 bp)	Reverse	AGG CCT CAC TGG CTA AAT CC	JAX
Gcn1 knockout	Forward	TAG AAA CAG GGT GTC TGT CTG TC	*
(302 bp)	Reverse	AGG CCT CAC TGG CTA AAT CC	JAX
Gmeb2 wild type	Forward	ATA CTT TTG TGG GGC GTA GC	JAX
(117 bp)	Reverse	CAT GCT CTA TCA GCC ACA GG	JAX
Gmeb2 knockout	Forward	ATA CTT TTG TGG GGC GTA GC	JAX
(157 bp)	Reverse	CCA TCA CCA CTA CCA CCA AGT	JAX
Got2 wild type	Forward	GTG GTG GCT CCA GGT TTT ACA CG	UCD
(489 bp)	Reverse	CTG GAG GTC CCA TTT CAA CAT GG	UCD
Got2 knockout	Forward	GTG GTG GCT CCA GGT TTT ACA CG	UCD
(443 bp)	Reverse	GCA GTG CTT GGT TGT GGT CTA GC	UCD
Kctd5 wild type	Forward	ACC TTC CAA GCT GCA GAA AA	*
(244 bp)	Reverse	AAA GCA GGT ACG CCC CTA GT	*
Kctd5 knockout	Forward	ACC TTC CAA GCT GCA GAA AA	*
(184 bp)	Reverse	GCT CTC CTC TCT GCT GTG CT	*

Development • Supplementary information

Pgm1 wild type	Forward	CTC AGT AAC ATG GTA GAG ATA TCC AAG	JAX
(111 bp)	Reverse	GAA GAA ATG CCA GGG GAA GA	JAX
Pgm1 knockout	Forward	CTC AGT AAC ATG GTA GAG ATA TCC AAG	JAX
(129 bp)	Reverse	TCA CAA AGC ACC AGG ACT CT	JAX
Ppox wild type	Forward	CCA CTG GCA AAA ACT AGG TAA G	JAX
(204 bp)	Reverse	GCG CCC ACT CTC TAT ACA GT	*
Ppox knockout	Forward	CCA CTG GCA AAA ACT AGG TAA G	JAX
(538 bp)	Reverse	TTA CTC TTC CAC CAG CAC GT	*
Rprd2 wild type	Forward	TTA AGG GTT TTT CGG TGC AT	JAX
(139 bp)	Reverse	AAC TGA AAA GCA GCC AGT GC	JAX
Rprd2 knockout	Forward	TTA AGG GTT TTT CGG TGC AT	JAX
(139 bp)	Reverse	TAT GCT CAC CCC ATT TTT GC	JAX
Slc30a9 wild type	Forward	TTT AAT GCT TTG GAC AGA ACA GTC	BCM
(512 bp)	Reverse	ACC CCT GGC TAT CTT GTA GAA	BCM
Slc30a9 knockout	Forward	TTT AAT GCT TTG GAC AGA ACA GTC	BCM
(376 bp)	Reverse	ATG AGC AGG GTT GAC TGG AG	BCM
Stt3b wild type	Forward	CCT TAA ACC TTG AAA ACT GGT TC	JAX
(108 bp)	Reverse	TGG CTA CAC ATA TCT CAC TAT GAA G	JAX
Stt3b knockout	Forward	CCT TAA ACC TTG AAA ACT GGT TC	JAX
(121 bp)	Reverse	TTC CCC CTT CTC TAG GAG TTC	JAX
Tedc2 wild type	Forward	TGC TTT TAG ACT CCG GTT GC	JAX
(101 bp)	Reverse	CAG CCA TGG TAT GGT CCA A	JAX
Tedc2 knockout	Forward	TGC TTT TAG ACT CCG GTT GC	JAX
(81 bp)	Reverse	CAA GCC CGG CAT TAG GAC T	JAX
Tgds wild type	Forward	ACA CTT TCC AGG ATC ACT CCA GCT	UCD
(491 bp)	Reverse	GTG CTT ACC TAC ATG TGT CTG TGC AG	UCD
Tgds knockout	Forward	ACA CTT TCC AGG ATC ACT CCA GCT	UCD
(505 bp)	Reverse	CGA CAA CGT GAG TGG CAG TCA G	UCD
Traf7 wild type	Forward	CAG GGC TTG AAT AGT GTT GTA AGG	JAX
(492 bp)	Reverse	TGT ATG AAG AGC TCT CCA ATC TGC	JAX
Traf7 knockout	Forward	GCT CTC TGC CTT GGT GTA GG	*
(540 bp)	Reverse	TGT GAC TTC TGG CTC TGG TG	*
Urm1 wild type	Forward	TGT TCT GTG CCT CCC TCT CT	*
(420 bp)	Reverse	TCC TGG CTG TCC TGA AAC TC	*
Urm1 knockout	Forward	GCT GGG AAC TGA GGT CTT TG	*
(305 bp)	Reverse	TCC TGG CTG TCC TGA AAC TC	*
Usb1 wild type	Forward	AGC TCT GGA AAG CAG GAT CA	*
(502 bp)	Reverse	ATC CAG TGG TGA CGG AGA AC	*
Usb1 knockout	Forward	AGC TCT GGA AAG CAG GAT CA	*
(503 bp)	Reverse	AAC TTG CGG GAT GAA GTC CT	*
Zmym2 wild type	Forward	GTG ATT TCC TGG ATC TAA AAA TGG	JAX
(158 bp)	Reverse	AAC AAT GGT TCC CTT CAT TG	JAX
Zmym2 knockout	Forward	GTG ATT TCC TGG ATC TAA AAA TGG	JAX
(168 bp)	Reverse	CAA CAA CCA AAA AGA GCA ATC	JAX

*primers designed in-house.
JAX, Primers from the genotyping protocol provided by the Jackson Laboratory.
UCD, Primers from the genotyping protocol provided by UC Davis.
TCP, Primers from the genotyping protocol provided by The Centre for Phenogenomics.
BCM, Primers from the genotyping protocol provided by the Baylor College of Medicine.

Available for download at
https://journals.biologists.com/dev/article-lookup/doi/10.1242/dev.205276#supplementary-data

Development • Supplementary information

Table S4. RT-PCR primers used to generate the cloned probe. All primers were designed in house, except for *Zmym2* (Gray et al., 2004).

Gene	RT Forward Primer (5' to 3')	RT Reverse Primer (5' to 3')
Alg3	TTC TGG CAC TGC TGA AGG AT	GTT CCA CCC ATG ATG CTG AG
Arpc1a	TGT CTC TGC ATC AGT TTC TGC	GCT CAC CCA CCA GTC ATT CT
Atp1b1	AGG CAG CTG GAA GAA ATT CA	CTG CAC ACC TTC CTC TCT CC
Bltp2	CCT GTT TGA GAT CCG GGA CT	TCC CAG TGC ATG TTC TCA GT
Chp1	GCT CCA CCC CTT GTT TCT CT	CTG TGG GAA AAG CCA GTC TC
Exoc3l4	GTA GCT GAG CAT GTG AAG GC	TGG GGT CAC ATG CTC AAG AT
Gcn1	ACT CAG GTC CCC ACA GTC AC	ACC ATT GGC CAG CTT AAC AC
Gmeb2	GAG GAA GTT TGT GTG CCC TG	CTG CAG GCC TTT CAT GGT TT
Got2	GAC CTC CAG ATC CCA TCC TG	GGA CAC TCT GCT CTG GGA TT
Kctd5	AAG ACC AGG CTG AGT TCC TC	CAT CTT CAG GAA TGC ACG GG
Pgm1	TCT TCG ATT TCA ACG CAC TG	TCT CCA CAG AGG GAC AGC TT
Ppox	TTT GCC GAG GAG TAT TTG CT	GAC AAG CTC CTC GGT ACT GC
Rprd2	TGG GCC CTC ACT AAC AGA AG	TTT CCG GTC CTC CAC AAT GA
Slc30a9	CCC GTA CGG CTT TTC AAA TA	CTT ACT GAC GGG TCG CTC TC
Stt3b	CAT TTT TGC GCT TCA GTT CA	AAG ACA CCC ATG TCG TAG GC
Tedc2	CAG AAC CCC CAC TCT GAC AT	TGA GTT TTG GCG GCT CTA GT
Tgds	TGG ATC AGG GCT TCA AAG GA	TCC TTC TTC CCA TGG CAC TT
Traf7	TTC GAC CGT CAC TAC CAT CA	CTC AGC GAC TGC AAT GTT GT
Urm1	CCC TGG GAT ATA CGG AAC CT	CTC CTG ACA CAG CAC AGG AA
Usb1	TTC TCC GTC ACC ACT GGA TC	AGC AGC CTA CCA ACC TAA GG
Zmym2	ACC AAG CAT ACT TCC AGA TGG G	GTA GAT GTG TAC CAG ACA GGG

Development • Supplementary information
