# Supplementary material for: Onset of embryonic and placental defects coincide in 19 of 22 novel mid-gestation lethal murine knockout lines
Source: Development. 2026 Jun 2;153(10):dev205276. doi: 10.1242/dev.205276 (PMC13286363; doi:10.1242/dev.205276)
Supplement: Supplementary file 1 [file develop-153-205276_review_history.pdf]

Onset of embryonic and placental defects coincide in 19 of 22 novel mid-gestation lethal murine knockout lines

Taylor M. Guertin, Chip Sisson, Rossella M. Gargiulo, Olivia Macrorie, Emily L. Pease, Hannah E. Garth, Joris Vriens, Katrien De Clercq, Jesse Mager and Kimberly D. Tremblay
DOI: 10.1242/dev.205276

Editor: Liz Robertson

Review timeline

Original submission:	27 September 2025
Editorial decision:	24 November 2025
First revision received:	24 March 2026
Editorial decision:	13 April 2026
Second revision received:	20 April 2026
Accepted:	22 April 2026

Original submission

First decision letter

MS ID#: dev.205276

MS TITLE: Onset of embryonic and placental defects coincide in 19 of 22 novel mid-gestation lethal murine knockout lines

AUTHORS: Kimberly D. Tremblay, Taylor M. Guertin, Jesse Mager, Katrien De Clercq, Emily L. Pease, Chip Sisson, Olivia Macrorie, Hannah E. Garth, Rossella M. Gargiulo and Joris Vriens

Dear Kim, Apologies for the delay in getting back to you about your paper. Unfortunately one of the reviewers who originally agreed to review the paper failed to submit the review inspite of numerous reminders! However I have now received two referees' reports on the above manuscript, and have reached a decision. The referees' comments are appended below.

As you will see, the referees express considerable interest in your work, but have some criticisms and recommend a substantial revision of your manuscript before we can consider publication. If you are able to revise the manuscript along the lines suggested, which may involve further experiments, I will be happy receive a revised version of the manuscript. Your revised paper will be re-reviewed by one or more of the original referees, and acceptance of your manuscript will depend on your addressing satisfactorily the reviewers' major concerns. Please also note that Development will normally permit only one round of major revision. If it would be helpful, you are welcome to contact us to discuss your revision in greater detail. Please send us a point-by-point response indicating your plans for addressing the referees' comments, and we will look over this and provide further guidance.

Please attend to all of the reviewers' comments and ensure that you upload both a 'clean' version of your Word file, along with a highlighted version clearly showing where you have made changes in the revised manuscript. Please avoid using 'Tracked changes' in Word files as these are lost in PDF conversion. I should be grateful if you would also provide a point-by-point response detailing how you have dealt with the points raised by the reviewers in the 'Response to Reviewers' box. If you do not agree with any of their criticisms or suggestions please explain clearly why this is so.

© 2026. Published by The Company of Biologists under the terms of the Creative Commons Attribution License (https://creativecommons.org/licenses/by/4.0/).

Reviewer 1

SUMMARY OF THE ADVANCE MADE IN THIS PAPER AND ITS POTENTIAL SIGNIFICANCE TO THE FIELD

Tremblay et al. describe 22 distinct mouse knock-out lines which are lethal from E9.5 and E10.5. Here they use a panel of several cell type markers to show defects in placental trophoblast-derived cells. Analysis of genetic knock-outs in the placenta is under-appreciated and a much-needed area of research. Assessment of 22 knock-outs in itself is an achievement. The authors describe a 'placental atlas' which, at present, is not comprehensive as it is lacking in many cell types of the placenta. There is also a fundamental lack of statistics to support findings from each model. Further, the embryonic phenotyping is vaguely described, which muddies the interpretation and potential commonalities between the knockouts. Attending to these major issues would increase my enthusiasm of this manuscript.

SUGGESTIONS TO AUTHORS

Major comments
The placental 'atlas' is currently lacking several placental cell types in both histology and in single cell datasets. These include infiltrating maternal decidua, immune cells (e.g. dNK cells), channel TGCs (lining maternal venous return), as well as all allantois derivatives including endothelial cells, pericytes, smooth muscle, fibroblasts, infiltrating fetal-derived macrophages etc. Considering the large proportion of labyrinth defects, I'm surprised that endothelial cells/vascular expansion and formation of other allantoic derivatives were not further investigated. Marsh et al. (2020) data used by the authors, does include endothelial cells (PECAM1/CD31+), pericytes (a-SMA+, Acta2+, NG2+) and smooth muscle cells (SM-MHC+, Myh11+), so it does not make sense why these cell types were removed from the analysis. Further, the described 'novel pipeline' is an overreach, with all the markers described being established elsewhere. Simmons et al. 2014 Chapter 12 Postimplantation Development of the Chorioallantoic Placenta, Guide to the Investigation of Mouse Pregnancy (https://doi.org/10.1016/B978-0-12-394445-0.00012-6) gives a comprehensive set of placental cell type markers, which may have escaped the authors attention. Furthermore, the single cell datasets were not created in house and have since been used by many to establish where genes of interest are expressed in the placenta, which reduces this 'novelty'.

Phenotyping of defects is all qualitative, and not quantitative. Can the authors provide statistical analysis for penetrance of placental and embryonic defects? E.g. Fisher's exact test for presence/absence of defects for KO vs littermate controls. Did the authors quantify any area occupied by the markers they describe? Figure 4 summarising this data seems inconsistent; Exoc3l4 for example shows defects in Gcm1 and Tfeb at E10.5 but not at E9.5. The data on the website at E9.5, however, shows there are likely to be changes already at this stage. Similarly for Arpc1a, website phenotyping at E9.5 shows Tpbpa and Tfeb staining are smaller in size, which are not represented in Figure 4. In this type of phenotyping without quantification, did two independent observers assess the staining?

Gross embryonic defects are poorly described. What were the 'gross defects' at E9.5 and E10.5 that categorised them at these stages? I suggest an additional supplemental table that describes what phenotypes were assessed. Abnormal heart and abnormal branchial arch are not highly descriptive. Was this determined from visualisation of the whole embryo and one midline section? There are publicly available datasets of the embryo which would help validate where genes of interest are expressed. Are they all in the cardiovascular system (a likely cause of embryonic lethality)? This will help understanding of the causes of embryo-placental demise and hypothesis generation for future studies.

* E6-E8.25 Mittnenzweig et al. 2021 Cell
* E9.5-E13.5 Cao et al. 2019 Nature
* E13.5 whole embryo dataset; Huang et al 2023 Nature.

Perez-Garcia et al. 2018 Nature, and Radford et al. 2023 Nat Communs highlight the importance of SynT-I layer in causing embryonic defects. Are you able to differentiate between SynT-I vs SynT-II defects? SynT-I and SynT-II are routinely identified with antibodies for MCT1 and MCT4 (Moreau et al. 2014 Developmental Biology, Radford et al. 2023 Nat Communs, Zhang et al. 2025 Nat Communs). Have the authors tried these?

© 2026. Published by The Company of Biologists under the terms of the Creative Commons Attribution License (https://creativecommons.org/licenses/by/4.0/).

Terminology
Line 79; 'embryonic mesoderm-derived allantois.' Mesoderm gives rise to embryonic and extra-embryonic portions; the allantois is an 'extra-embryonic' tissue.
Line 82-83; 'two fetal derived layers, the junctional zone and labyrinth zone'. 'Fetus' is the wrong term here as it describes the embryo proper. Trophoblast or trophectoderm-derived is appropriate. Similarly for other areas of the manuscript e.g. line 339 etc Line 198-199; the authors describe 'allantoic mesoderm' in primary villi. Mesodermal markers including T (Brachyury) from my understanding are turned off by this stage. Can you provide a reference or ISH for this? Could this be instead ECs and other mesenchymal cells?

Minor comments
Line 74 typo; 'is critical a bottleneck' or 'is a critical bottleneck'
Results section changed from past tense to present tense which is odd.

Reviewer 2

This manuscript by Taylor Guertin and colleagues describes the phenotypic characterization of 22 mouse KO lines that exhibit intrauterine lethality. The particular emphasis of this study is on the assessment of the placenta to determine the frequency with which placental defects can be detected in cases of mid-gestational embryonic lethality. The authors apply a series of stainings to determine placental differentiation defects, and broadly categorize their mutants into different classes depending on the precise phenotype observed.

The study was clearly a vast effort with the import of 22 IMPC/KOMP em1 KO mouse lines, the set-up of heterozygous intercrosses and the systematic capture of embryonic and placental phenotypes. As such, the study is a good fit with the target journal.

There are, however, a number of more serious issues with the current manuscript that need to be addressed.

The authors describe a "novel placental phenotyping pipeline" which implies that this is superior to previous efforts. This is not necessarily the case, and some of this needs to be addressed:
- Given that many other studies have assessed placental phenotypes with various markers, the word "novel" should be taken out of this description throughout, and it simply be referred to as "placental phenotyping pipeline applied".
- The pipeline completely omits the analysis of TGC markers, and makes a somewhat peculiar choice on the "labyrinth" marker Tfeb. As such, the study would tremendously benefit from an ISH analysis for Plf (or a similarly strong TGC marker).
- Perhaps more importantly even, the study should employ stainings for the well-established SynT markers MCT1 and MCT4. Given that 22 mouse lines were investigated, this would mean another 22 double-IF stainings (for MCT1&MCT4) and 22 ISHs (for Plf), which does not seem too unreasonable and would tremendously improve the interpretability of the data.
- At the very least, this should be performed for the select lines where this is of particular importance (i.e., lines with reduced/expanded JZ need a TGC marker assessment, lines with "Gcm1+ cell penetrating through Tfeb+ region" need MCT1/4 assessment).

The crux with Tfeb is that its endogenous expression pattern is not well defined. The stainings on the earlier placentas show that, in general, Tfeb stains the cell layer(s) just above Gcm1+ cells. The most likely interpretation is that Tfeb is expressed in an intermediate progenitor and that SynT-I cells emerge on the upper edges of the Tfeb+ domain. This would be in line with Tfeb being described as a SynT-I progenitor and SynT-I marker in the Marsh&Blelloch snRNA-seq study. What is almost certainly NOT the case is that Tfeb is a pan-labyrinth trophoblast marker, as described in the text, and the old reference does not provide sufficient resolution on this point. Ultimately, double stainings (IF or ISH) would be needed to fully resolve the question around what cell type(s) Tfeb demarcates exactly, but an inclusion of the above-mentioned MCT1/4 stainings would at least provide reasonable clarity on this point.

Following on from the not-well-defined expression domain of Tfeb, the authors describe that in some mutants, Gcm1+ cells seem to "reach through" the Tfeb+ region. This, however, appears to

© 2026. Published by The Company of Biologists under the terms of the Creative Commons Attribution License (https://creativecommons.org/licenses/by/4.0/).

happen during normal development between E9.5-E10.0, which can be seen in Extended Figure 1 as well as in Figure 3. Hence, the Exoc3l4-mutant phenotype may be an indicator of premature differentiation of certain trophoblast subtypes. The phenotype description should be reworded to leave this possibility open, and/or the text re-evaluated after the above stainings have been performed.

The reference to the Placenta Atlas, or even "newly developed" Placenta Atlas, should be reworded to something like "Characterizing placental development across a mid-gestational time course", or similar. The Placenta Atlas terminology is somewhat aggrandized; while it is very nice and was a lot of work, there is nothing new to this compilation, even with the gene expression patterns which have largely been reported in Simmons et al. 2008. Also, the quarter-day staging is likely inaccurate and perhaps an 'overkill', see for example E8.75 vs E9.0 where placental development may have been captured in a discontinuous way (more domed chorion morphology of the E9.0 placenta compared to the E8.75, in theory it should be inverse).

The manuscript states that serial placental sections were used for the histology analysis. Clicking through their cited database, this is not always the case; in fact it is quite frequently not the case.

The authors conclude that 19 out of the 22 lines they investigated have a placental phenotype. However, the other 3 lines are those that die even earlier. Therefore, it is possible that these, too, have an extraembryonic phenotype in the trophoblast lineage or in the yolk sac, but the stages assessed and markers employed would not capture this. Hence, it might be better to say that those 19 with a mid-gestational lethality all have a placental phenotype, and refer to the other three as an earlier-onset lethality.
Indeed, a closer inspection of all three lines seems to show a lack of fetal blood in the developing placenta, and at least for Atp1b1 and perhaps Tedc1 a delamination of the visceral yolk sac endoderm from the mesoderm (i.e., the blood islands are not as clearly defined as in the controls). For Ehbp1, the histology is too poor to tell. The authors can either re-check their sections to determine YS phenotypes, and/or leave this possibility open that other extra-embryonic phenotypes may be present.

Other wording issues:
E9.5-onset phenotype: This is incorrect, since several of these lines already have an overt phenotype at E9.5 and even earlier at E8.5. So the "onset" is not at E9.5, it should rather be "E9.5 phenotype" lines.

Lines 73-74, fix sentence structure.

Line 94, "phenotypes" (plural)

Line 108 and other places, "heterozygous KO" is an oxymoron

Line 128 and elsewhere, what is an "intermediate lethal KO line"?

Line 129, see also above, the "onset" problem

Lines 241/242, it is exceedingly difficult - if not impossible - to make out the two syncytiotrophoblast layers in H&E stainings. Hence the recommendation about the added MCT1/4 staining detailed above.
Also, what is an "attenuated morphology"?

Lines 256/257, and also Discussion lines 387-391, please see above about YS defects (or other, earlier trophoblast defects).

JZ defects section: Whether or not the JZ is expanded or reduced makes most sense in conjunction with knowledge of TGC differentiation. Most commonly, mouse lines with a smaller EPC/JZ have an overabundance or over-enlargement of TGCs. Historically, this bifurcation is exemplified by the Ascl2 mutant (precocious exit from a mitotic cell cycle to an endoredupplicative cell cycle, i.e. more TGCs at the expense of diploid SpT cells).

© 2026. Published by The Company of Biologists under the terms of the Creative Commons Attribution License (https://creativecommons.org/licenses/by/4.0/).

Lines 310/311, the lollipops appear to highlight maternal blood spaces, please verify that these are indeed fetal, as described.

Lines 336/337, "sectioned ISH", is this ISH on sections, or whole-mount ISH sectioned? Presumably the former.

First revision

Author response to reviewers' comments

We wholeheartedly thank the reviewers for their insight and critique of our manuscript. We realize that reviewing this manuscript was a massive effort. We agree with your critiques and have taken extensive efforts to address almost all as outlined below. The reviewers critiques are provided in *italics* and how it was addressed in bold. Because the revision was extensive, we did not include all revised text nor the Extended data updates here. We have highlighted in yellow the major revisions in the "highlighted Manuscript" portion of the submission.

Reviewer 1: SUMMARY OF THE ADVANCE MADE IN THIS PAPER AND ITS POTENTIAL SIGNIFICANCE TO THE FIELD

Tremblay et al. describe 22 distinct mouse knock-out lines which are lethal from E9.5 and E10.5. Here they use a panel of several cell type markers to show defects in placental trophoblast-derived cells. Analysis of genetic knock-outs in the placenta is under-appreciated and a much-needed area of research. Assessment of 22 knock-outs in itself is an achievement. The authors describe a 'placental atlas' which, at present, is not comprehensive as it is lacking in many cell types of the placenta. There is also a fundamental lack of statistics to support findings from each model.

Further, the embryonic phenotyping is vaguely described, which muddies the interpretation and potential commonalities between the knockouts. Attending to these major issues would increase my enthusiasm of this manuscript.

SUGGESTIONS TO AUTHORS

Major comments
The placental 'atlas' is currently lacking several placental cell types in both histology and in single cell datasets. These include infiltrating maternal decidua, immune cells (e.g. dNK cells), channel TGCs (lining maternal venous return), as well as all allantois derivatives including endothelial cells, pericytes, smooth muscle, fibroblasts, infiltrating fetal-derived macrophages etc. Considering the large proportion of labyrinth defects, I'm surprised that endothelial cells/vascular expansion and formation of other allantoic derivatives were not further investigated. Marsh et al. (2020) data used by the authors, does include endothelial cells (PECAM1/CD31+), pericytes (a-SMA+, Acta2+, NG2+) and smooth muscle cells (SM-MHC+, Myh11+), so it does not make sense why these cell types were removed from the analysis.

We did not mean for the former "placental atlas" to be comprehensive. We produced it so that we and others would have an accessible resource that describes normal murine placental development on sections during this narrow window of development. It complements and facilitates the use of our streamlined placental analysis approach. We acknowledge that this approach has shortcomings and does not analyze all cell types and this is stated in the penultimate paragraph of the discussion as quoted below.

"The streamlined phenotyping pipeline used herein identified specific placental defects that coincide with mid-gestion embryo phenotypic onset in most KO lines examined, however it is not without limitations. The analysis pipeline did not molecularly assess the YS and it is possible that YS or trophectoderm-specific defects contribute to embryonic demise. Similarly,

© 2026. Published by The Company of Biologists under the terms of the Creative Commons Attribution License (https://creativecommons.org/licenses/by/4.0/).

the pipeline does not directly examine maternal spiral artery remodeling or the TGC subtypes that line the channels and deliver maternal blood into the labyrinth. In our approach, their differentiation is indirectly inferred by the presence of maternal blood in the trophoblast compartment. Nonetheless, the pipeline provides a wealth of information using cell-type specific analyses, making it well suited for identifying many aspects of the mid-gestation placenta.

Furthermore, we now include an "all cells" analysis of the Marsh and Blelloch data in SFig3 (see below).

Supplemental Figure 7: Expression of mid-gestation lethal genes in trophoblast clusters from published RNA-seq datasets.

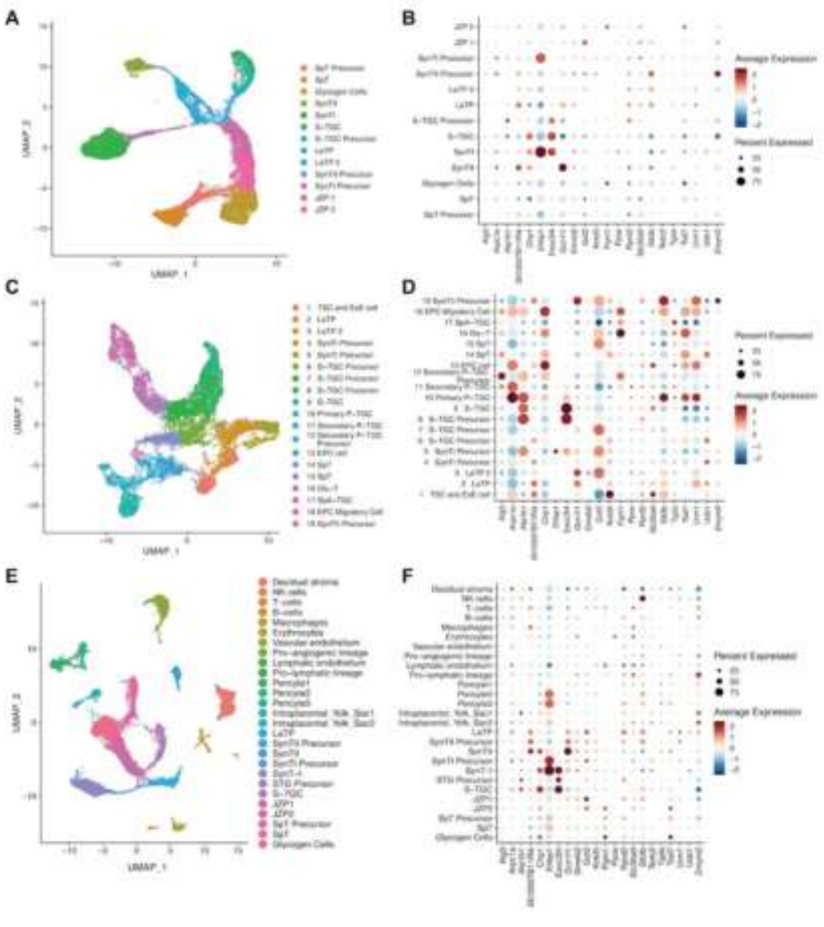

Further, the described 'novel pipeline' is an overreach, with all the markers described being established elsewhere. Simmons et al. 2014 Chapter 12 Postimplantation Development of the Chorioallantoic Placenta, Guide to the Investigation of Mouse Pregnancy (https://doi.org/10.1016/B978-0-12-394445-0.00012-6) gives a comprehensive set of placental cell type markers, which may have escaped the authors attention.

We have deleted the word "novel" in relation to the analysis pipeline. We did not mean to imply that the markers we were using were novel, nor did we mean to convey that the atlas was comprehensive. Our intention was that the streamlined approach we used and the eventual accessible nature of our manuscript would facilitate its use by others, particularly relevant since placental defects are drastically underreported. The Simmons 2014 book chapter is cited in the current MS when describing the chorioallantoic branching process (lines 189) and for us the electronic version is behind a paywall (we checked out the hardcopy a few years ago and returned it a few months ago). This resource does not include accompanying histology. For the *in situ* markers used to create our mid-gestation placenta guide, we cited original sources (lines 149-154).

© 2026. Published by The Company of Biologists under the terms of the Creative Commons Attribution License (https://creativecommons.org/licenses/by/4.0/).

Furthermore, the single cell datasets were not created in house and have since been used by many to establish where genes of interest are expressed in the placenta, which reduces this 'novelty'.

We agree that the single cell datasets are extremely useful resources that have been widely utilized and that their use in our paper is not novel.

Phenotyping of defects is qualitative, and not quantitative. Can the authors provide statistical analysis for penetrance of placental and embryonic defects? E.g. Fisher's exact test for presence/absence of defects for KO vs littermate controls.

Because of the streamlined nature of our placental phenotyping pipeline, all placental defects are qualitative. We were very strict with our designations and only scored a placental phenotype with a green box when all 3 of the placentas analyzed demonstrated the phenotype. If variability was detected, additional placentas were examined and the phenotype scored as present when more than half of the mutant placentas displayed the phenotype (hashed marks on the green phenotype box).

We now present calculated p-values for each of the embryo phenotypes listed in the chart presented in SFig 2 (below). A 2-tailed Fisher's exact test was performed using the number of embryos in each category that displayed the presence and absence of each phenotype in the mutant versus all littermate controls. A p-value less than or equal to 1 X 10^{-4} was used as a threshold for significance. All significant phenotypes are now listed in SFig2 with an asterisk. The phenotyping chart now includes the number of mutant embryos examined for each line (n). At the bottom of each chart we now include a row that indicates the phenotype penetrance in all control littermates.

Such significance tests were not feasible for the placenta defects since the control placentas themselves were not scored as normal/abnormal but instead were considered normal. Abnormalities in KO placentas were determined after direct comparison to the littermate controls that were all processed identically.

© 2026. Published by The Company of Biologists under the terms of the Creative Commons Attribution License (https://creativecommons.org/licenses/by/4.0/).

Supplemental Figure 2: Summary of embryonic defects observed in all 22 KO lines

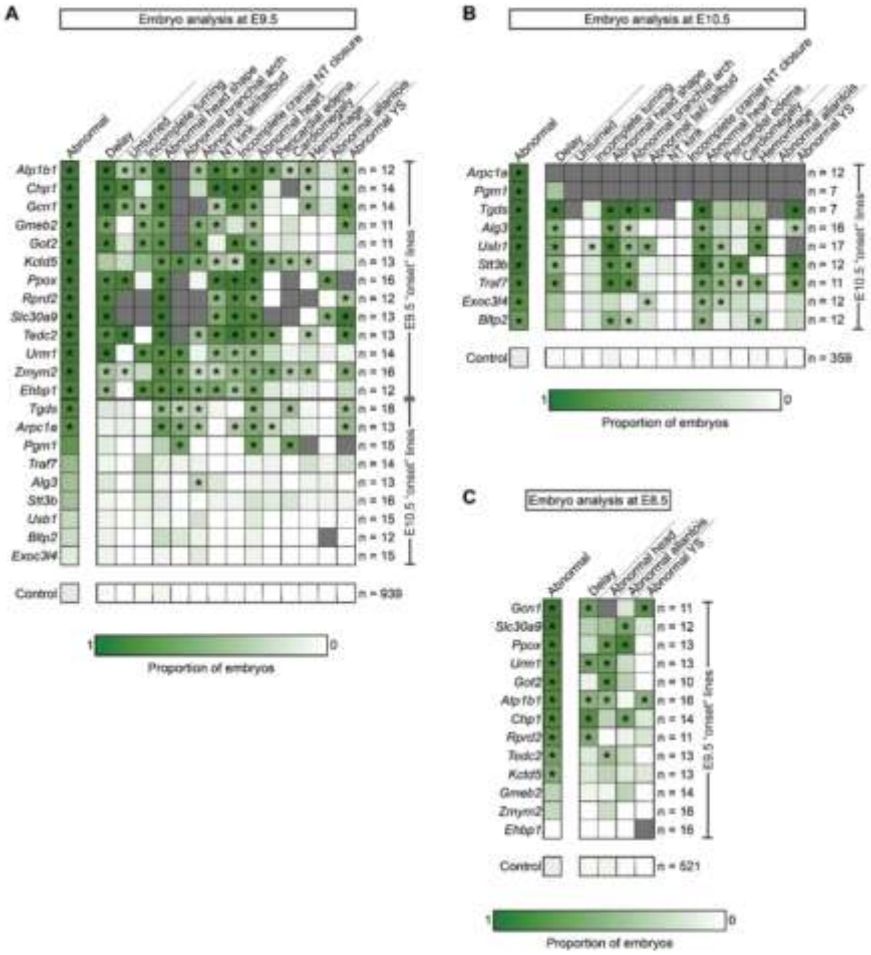

Did the authors quantify any area occupied by the markers they describe?

We did not perform an area analysis, because there were not enough sections from each placenta to perform the required statistics.

Figure 4 summarising this data seems inconsistent; Exoc3l4 for example shows defects in Gcm1 and Tfeb at E10.5 but not at E9.5. The data on the website at E9.5, however, shows there are likely to be changes already at this stage. Similarly for Arpc1a, website phenotyping at E9.5 shows Tpbpa and Tfeb staining are smaller in size, which are not represented in Figure 4.

Thank you for looking carefully at our extended data and correlating it with the Fig 4 phenotyping summary. As stated above, we tried to be very stringent with classifying abnormal phenotypes. It is possible that with a more quantitative approach, which would require the analysis of more placentas, we would be able to identify and parse out more subtle phenotypes. However due to the scope of the work, we presented only the most striking and penetrant phenotypes.

Regarding the particular lines in question. For *Exoc3l4*, the second data reviewer agreed with you and when prompted by your comment, the first data reviewer went back to re-review the data, agreed. That E9.5 phenotype call is now updated (see Fig. Both reviewers stated that no phenotype is apparent in the *Tpbpa*+ layer at E10.5. Both reviewers agree

© 2026. Published by The Company of Biologists under the terms of the Creative Commons Attribution License (https://creativecommons.org/licenses/by/4.0/).

that there was no phenotype for either *Tpbpa* or *Tfeb* at E9.5 for the *Arpc1a* mutant placentas.

In this type of phenotyping without quantification, did two independent observers assess the staining?

The images were identified independently by two individuals and that is now incorporated into the last sentence of the Placenta Phenotyping and Analysis section of the Methods.

Gross embryonic defects are poorly described. What were the 'gross defects' at E9.5 and E10.5 that categorised them at these stages? I suggest an additional supplemental table that describes what phenotypes were assessed. Abnormal heart and abnormal branchial arch are not highly descriptive. Was this determined from visualisation of the whole embryo and one midline section?

Because of the size of the data set and our interest in focusing on the extraembryonic defects, we intentionally did not focus on particular embryonic phenotypes in detail in the manuscript. To categorize each we used the mammalian phenotype (MP) terms that describe defects in embryonic processes (such as embryonic turning or allantois extension) or structures (listed below). These defects can be identified by a trained eye with extensive experience in normal/abnormal development between E8.5-10.5 (KDT performed almost all dissections and analysis, and much was concurrently analyzed first author or by other contributing authors who were assigned a line. Only the processes and structures that were scored as abnormal in multiple lines were documented in our chart (Fig. S2). The exact mammalian MP terms are explained in MGI include the following:

Delay: MP003984
Unturned: MP000418
Incomplete embryo turning: MP
0001701 Neural Tube Kink:
MP0003400
Abnormal head development:
MP0030325 Incomplete cranial neural
tube closure: MP0000928 Abnormal
heart development: MP0000267
Pericardial edema:MP0001787
Abnormal allantois morphology:
MP0001726 Abnormal visceral yolk sac
morphology: MP0001718 Abnormal
branchial arch morphology: MP0002884
Abnormal tail bud
morphology:MP0002861
Enlarged heart (cardiomegaly): MP:0000274
Hemorrhage: MP001914 (while this term is not typically associated with embryonic defects, we included because several KO lines consistently had visible abnormal blood deposition in embryo or yolk sac when compared with littermates indicating an anomaly in vascular or heart defects.)

Other gross structures examined at E9.5-10.5 include limb bud morphology, regularity in somite formation, caudal neural tube closure, presence of optic eminence, and the presence of the otic pit. None of the lines examined had consistent defects in any of these structures and thus were not included in the phenotype analysis chart.

All phenotyping information is also displayed with additional details on our publicly accessible blog and is also integrated with MGI and IMPRESS (IMPC). We are preparing manuscripts that highlight specific aspects of several of the lines examined herein.

There are publicly available datasets of the embryo which would help validate where genes of interest are expressed. Are they all in the cardiovascular system (a likely cause of embryonic

© 2026. Published by The Company of Biologists under the terms of the Creative Commons Attribution License (https://creativecommons.org/licenses/by/4.0/).

lethality)? This will help understanding of the causes of embryo-placental demise and hypothesis generation for future studies.

**E6-E8.25 Mittnenzweig et al. 2021 Cell*
**E9.5-E13.5 Cao et al. 2019 Nature*
**E13.5 whole embryo dataset; Huang et al 2023 Nature.*

We felt that this information is beyond the scope of this paper and have not included this information in the MS. Our blog has additional information on expression, and this data has also been integrated into the MGI expression database which can be accessed the gene name.

Perez-Garcia et al. 2018 Nature, and Radford et al. 2023 Nat Communs highlight the importance of SynT-I layer in causing embryonic defects. Are you able to differentiate between SynT-I vs SynT-II defects? SynT-I and SynT-II are routinely identified with antibodies for MCT1 and MCT4 (Moreau et al. 2014 Developmental Biology, Radford et al. 2023 Nat Communs, Zhang et al. 2025 Nat Communs). Have the authors tried these?

In response to both reviewers' comments, the phenotyping pipeline and accompanying analysis of all has been expanded to include SynTI, SynTII and endothelial markers (MCT4, MCT1 and Endomucin, respectively). This panel of markers was performed on all mutant and control placentas examined in the MS using the extra slide that had been collected from each. This new data is now integrated throughout the MS, has been added to Figs 2 (minor update in B), 3, 5, 6 (and their legends) and is incorporated into all Extended Data Figs (1-23 and their legends). Figure 3 and its legend is pasted below.

© 2026. Published by The Company of Biologists under the terms of the Creative Commons Attribution License (https://creativecommons.org/licenses/by/4.0/).

Figure 3: Histological and molecular analysis of E8.5-E10.5 placenta development.

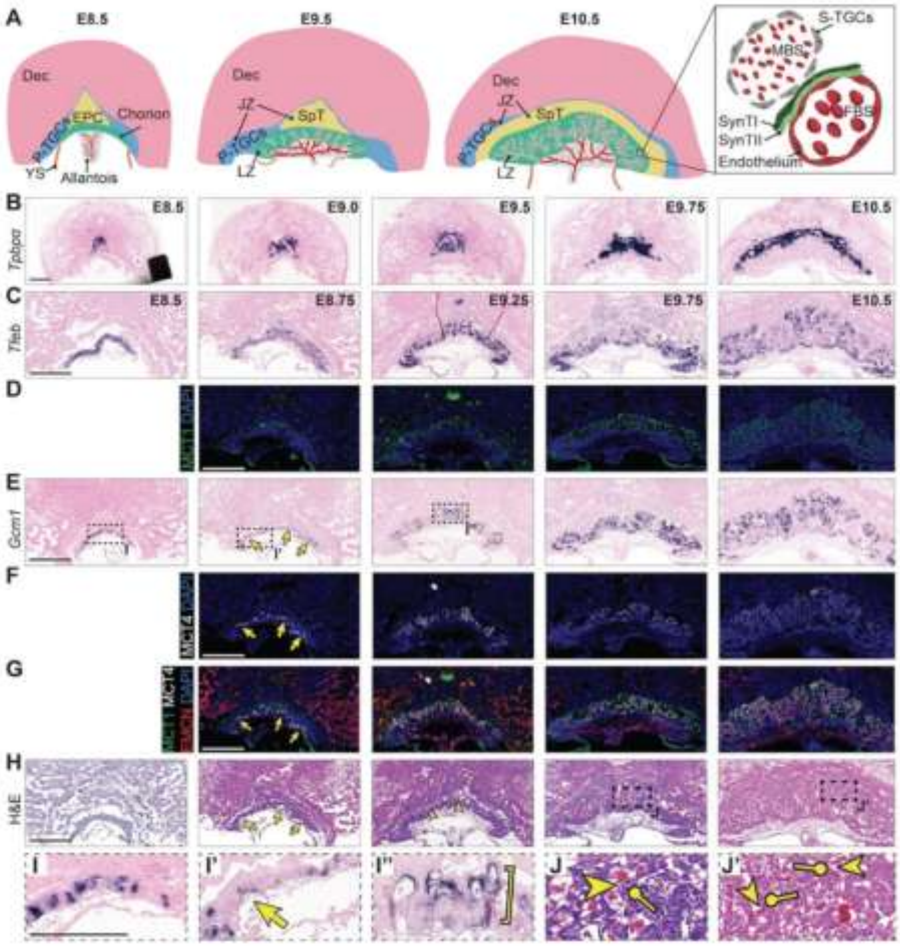

Figure 3: Analysis of early mid-gestation placentation using a streamlined histological and molecular analysis of the E8.5-E10.5 placenta. (A) Overview of mid-gestation placental morphogenesis (E8.5-E10.5). At E8.5, the vascularized allantois is attached to the chorion while the ectoplacental cone (EPC), containing spongiotrophoblast (SpT) progenitors, is positioned atop the chorion. Parietal trophoblast giant cells (P-TGCs) line the maternal decidua (Dec) throughout gestation. By E9.5, chorioallantoic branching incorporates allantois-derived vasculature and fetal blood into the LZ. At E10.5, differentiated cells of the interhemal barrier include: an endothelium, two tightly apposed layers of syncytiotrophoblasts (SynTI and SynTII), and sinusoidal TGCs (S-TGCs). This barrier separates maternal (MBS) and fetal (FBS) blood spaces within the LZ. (B-E) *In situ* hybridization (ISH), immunofluorescence, and hematoxylin and eosin (H&E) staining of E8.5-E10.5 control placentas. Because of size limitations, all sections of an E8.5 placenta were subjected to a single ISH probe or H&E staining, however, for E8.75-E10.5 placentas, adjacent sections were used for analysis except as noted. (B) *Tpbpa* expression in E8.5-E10.5 placentas demonstrates the developmental progression of the SpT layer. (C-H) *Tfeb*, MCT1, *Gcm1*, MCT4, Endomucin, and H&E staining of sectioned E8.5-E10.5 placentas illustrate LZ morphogenesis. Boxes in *Gcm1* and H&E-stained images indicate the LZ region in the magnified insets at the bottom (I-I'', J-J'). (C) At E8.5, the SynTI marker *Tfeb* displays uniform expression of the chorion, except for the basal-most layer. As the interhemal barrier differentiates, this layer expands to form the LZ. (D) MCT1 is expressed in the SynTI layer of the LZ. Unlike *Tfeb*, MCT1 is enriched at the JZ border and is absent in basal chorionic trophoblasts adjacent to the chorioallantoic interface. (E) At E8.5, clusters of *Gcm1*+ cells are observed sporadically along the basal chorion. At E8.75, signs of chorion involution and invasion

© 2026. Published by The Company of Biologists under the terms of the Creative Commons Attribution License (https://creativecommons.org/licenses/by/4.0/).

of the allantois are observed at sites of *Gcm1* expression (arrow). Villi with an allantois-derived core are outlined by *Gcm1* at the distal end as they elongate through the LZ (bracket). The formation of additional villi and secondary branching morphogenesis contributes to the expansion of the E10.5 *Gcm1*+ network.
(F) The SynTII marker MCT4 overlaps with *Gcm1* to define chorioallantoic branching and SynTII layer formation. However, MCT4 expression is more uniform along the SynTII layer than *Gcm1*.
(G) Endomucin (EMCN) immunostaining reveals allantoic-derived fetal vasculature incorporation into the LZ. At E8.75, endothelial cells are present in the allantoic mesenchyme integrating into the LZ through chorioallantoic branching, adjacent to the developing SynTII MCT4+ layer. As interhemal barrier development proceeds, a more extensive fetal vasculature network is formed. At E10.5, MCT1+ SynTI cells are largely adjacent to MCT4+ SynTII cells which separate Endomucin-lined fetal blood spaces and maternal blood spaces. (H) By E8.75, maternal and fetal blood cells are interspersed among labyrinth trophoblasts. Fetal blood cells (lollipop, J-J’) identified by their large, round morphology and hematoxylin-reactive nuclei are distinguished from the smaller, enucleated maternal red blood cells. During development, maternal and fetal blood spaces accumulate and become closely juxtaposed (arrowhead). Elongated SynT cells of the interhemal barrier are observed between maternal and fetal blood spaces (arrowhead). Scale bars: 500 μm (B-E) or 250 μm for the higher-magnification images (I-I’’, J-J’).

Terminology
Line 79; 'embryonic mesoderm-derived allantois.' Mesoderm gives rise to embryonic and extra-embryonic portions; the allantois is an 'extra-embryonic' tissue.

We now state “mesoderm-derived allantois” (line 77).

Line 82-83; 'two fetal derived layers, the junctional zone and labyrinth zone'. 'Fetus' is the wrong term here as it describes the embryo proper. Trophoblast or trophectoderm-derived is appropriate. Similarly for other areas of the manuscript e.g. line 339 etc

We now use trophoblast or trophectoderm-derived tissue as suggested (lines 78-80; 365-366). We sometimes use “fetal” or “fetal derived" when also referring to the maternal component of the placenta.

Line 198-199; the authors describe 'allantoic mesoderm' in primary villi. Mesodermal markers including T (Brachyury) from my understanding are turned off by this stage. Can you provide a reference or ISH for this? Could this be instead ECs and other mesenchymal cells?

We now use the phrase “allantoic mesenchyme” or “allantois-derived mesenchyme” when referring to the allantois derivatives.

Minor comments
Line 74 typo; 'is critical a bottleneck' or 'is a critical bottleneck'

Changed- thanks.

Results section changed from past tense to present tense which is odd.

We have now made the tense consistent. Thank you.

Reviewer 2: This manuscript by Taylor Guertin and colleagues describes the phenotypic characterization of 22 mouse KO lines that exhibit intrauterine lethality. The particular emphasis of this study is on the assessment of the placenta to determine the frequency with which placental defects can be detected in cases of mid-gestational embryonic lethality. The authors apply a series of stainings to determine placental differentiation defects, and broadly categorize their mutants into different classes depending on the precise phenotype observed.

The study was clearly a vast effort with the import of 22 IMPC/KOMP em1 KO mouse lines, the set-up of heterozygous intercrosses and the systematic capture of embryonic and placental phenotypes. As such, the study is a good fit with the target journal.

© 2026. Published by The Company of Biologists under the terms of the Creative Commons Attribution License (https://creativecommons.org/licenses/by/4.0/).

Thank you for this recognition.

There are, however, a number of more serious issues with the current manuscript that need to be addressed.

The authors describe a "novel placental phenotyping pipeline" which implies that this is superior to previous efforts. This is not necessarily the case, and some of this needs to be addressed:
- Given that many other studies have assessed placental phenotypes with various markers, the word "novel" should be taken out of this description throughout, and it simply be referred to as "placental phenotyping pipeline applied".

To address this critique, as suggested by both reviewers, we have removed the words novel when describing the placental phenotyping pipeline.

- The pipeline completely omits the analysis of TGC markers, and makes a somewhat peculiar choice on the "labyrinth" marker Tfeb. As such, the study would tremendously benefit from an ISH analysis for Plf (or a similarly strong TGC marker).

- Perhaps more importantly even, the study should employ stainings for the well-established SynT markers MCT1 and MCT4. Given that 22 mouse lines were investigated, this would mean another 22 double-IF stainings (for MCT1&MCT4) and 22 ISHs (for Plf), which does not seem too unreasonable and would tremendously improve the interpretability of the data.

- At the very least, this should be performed for the select lines where this is of particular importance (i.e., lines with reduced/expanded JZ need a TGC marker assessment, lines with "Gcm1+ cell penetrating through Tfeb+ region" need MCT1/4 assessment)

To address these interrelated critiques, we have performed IF with MCT1/MCT4 and Endomucin on the fifth adjacent section slide that we saved from each of the mutant/control littermate pairs examined in the first version of this manuscript. This new IF data has been extensively incorporated throughout the MS (Fig. 2,3,5,6) and is incorporated into all Extended Data Figs (1-23 and their legends). The revised Figure 3 and legend is pasted below.

© 2026. Published by The Company of Biologists under the terms of the Creative Commons Attribution License (https://creativecommons.org/licenses/by/4.0/).

Figure 3: Histological and molecular analysis of E8.5-E10.5 placenta development.

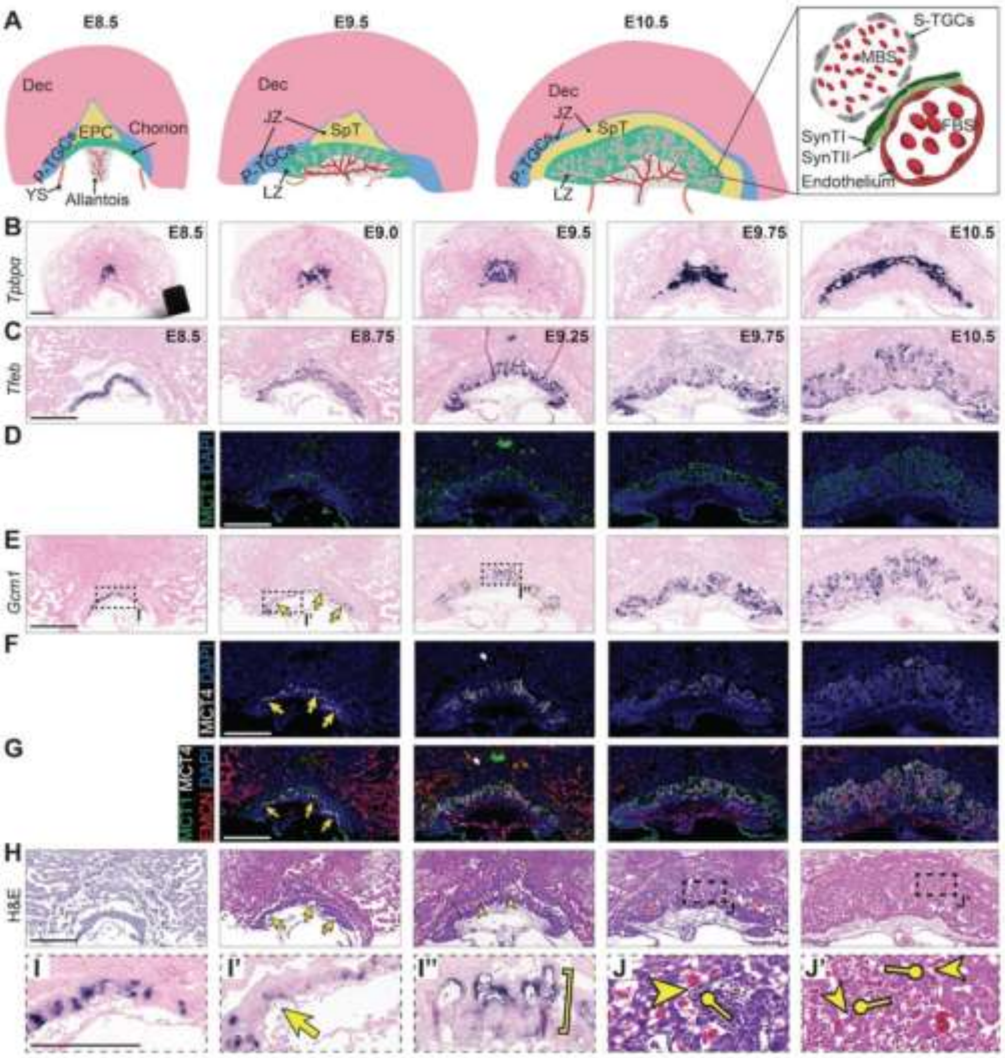

Figure 3: Analysis of early mid-gestation placentation using a streamlined histological and molecular analysis of the E8.5-E10.5 placenta. (A) Overview of mid-gestation placental morphogenesis (E8.5-E10.5). At E8.5, the vascularized allantois is attached to the chorion while the ectoplacental cone (EPC), containing spongiotrophoblast (SpT) progenitors, is positioned atop the chorion. Parietal trophoblast giant cells (P-TGCs) line the maternal decidua (Dec) throughout gestation. By E9.5, chorioallantoic branching incorporates allantois-derived vasculature and fetal blood into the LZ. At E10.5, differentiated cells of the interhemal barrier include: an endothelium, two tightly apposed layers of syncytiotrophoblasts (SynTI and SynTII), and sinusoidal TGCs (S-TGCs). This barrier separates maternal (MBS) and fetal (FBS) blood spaces within the LZ. (B-E) *In situ* hybridization (ISH), immunofluorescence, and hematoxylin and eosin (H&E) staining of E8.5-E10.5 control placentas. Because of size limitations, all sections of an E8.5 placenta were subjected to a single ISH probe or H&E staining, however, for E8.75-E10.5 placentas, adjacent sections were used for analysis except as noted. (B) *Tpbpa* expression in E8.5-E10.5 placentas demonstrates the developmental progression of the SpT layer. (C-H) *Tfeb*, MCT1, *Gcm1*, MCT4, Endomucin, and H&E staining of sectioned E8.5-E10.5 placentas illustrate LZ morphogenesis. Boxes in *Gcm1* and H&E-stained images indicate the LZ region in the magnified insets at the bottom (I-I'', J-J'). (C) At E8.5, the SynTI marker *Tfeb* displays uniform expression of the chorion, except for the basal-most layer. As the interhemal barrier differentiates, this layer expands to form the LZ. (D) MCT1 is expressed in the SynTI

© 2026. Published by The Company of Biologists under the terms of the Creative Commons Attribution License (https://creativecommons.org/licenses/by/4.0/).

layer of the LZ. Unlike *Tfeb*, MCT1 is enriched at the JZ border and is absent in basal chorionic trophoblasts adjacent to the chorioallantoic interface. (E) At E8.5, clusters of *Gcm1*+ cells are observed sporadically along the basal chorion. At E8.75, signs of chorion involution and invasion of the allantois are observed at sites of *Gcm1* expression (arrow). Villi with an allantois-derived core are outlined by *Gcm1* at the distal end as they elongate through the LZ (bracket). The formation of additional villi and secondary branching morphogenesis contributes to the expansion of the E10.5 *Gcm1*+ network.
(F) The SynTII marker MCT4 overlaps with *Gcm1* to define chorioallantoic branching and SynTII layer formation. However, MCT4 expression is more uniform along the SynTII layer than *Gcm1*.
(G) Endomucin (EMCN) immunostaining reveals allantoic-derived fetal vasculature incorporation into the LZ. At E8.75, endothelial cells are present in the allantoic mesenchyme integrating into the LZ through chorioallantoic branching, adjacent to the developing SynTII MCT4+ layer. As interhemal barrier development proceeds, a more extensive fetal vasculature network is formed. At E10.5, MCT1+ SynTI cells are largely adjacent to MCT4+ SynTII cells which separate Endomucin-lined fetal blood spaces and maternal blood spaces. (H) By E8.75, maternal and fetal blood cells are interspersed among labyrinth trophoblasts. Fetal blood cells (lollipop, J-J') identified by their large, round morphology and hematoxylin-reactive nuclei are distinguished from the smaller, enucleated maternal red blood cells. During development, maternal and fetal blood spaces accumulate and become closely juxtaposed (arrowhead). Elongated SynT cells of the interhemal barrier are observed between maternal and fetal blood spaces (arrowhead). Scale bars: 500 µm (B-E) or 250 µm for the higher-magnification images (I-I'', J-J').

We also sectioned additional whole placentas (minimum of 2) from the 11 of the 12 lines that displayed JZ defects with *Tpbpa* and placed alternating sections onto 2 slides (*Exoc3l4* was not examined because we did not have additional placentas that could be used for *in situ*). One slide was subjected to *Pl-1 in situ* hybridization and the other to *Tpbpa in situ* hybridization. TGC defects have been identified in 6 of the 11 lines analyzed and this additional information has been integrated into Figs. 4-and into all relevant Extended Data Figures (5, 8-17). An example of the incorporation of the new data into the MS (on line 272-286) is copied below.

"*JZ defects*
The size of the *Tpbpa* + SpT layer, the main JZ layer examined, was altered in roughly half of the lines (Fig. 4, n=12/22). SpTs and TGCs are hypothesized to arise from a shared precursor and abnormalities in one layer of mutant placentas often coincide with opposing abnormalities in the other layer, although this relationship is not always observed (Simmons and Cross, 2005). To examine the co-occurrence of P-TGC layer abnormalities, additional available placentas belonging to 11 of the lines with SpT layer abnormalities were examined and adjacent sections were stained with the P-TGC marker *Pl-1* and *Tpbpa*. A reduced SpT layer was associated with a thinner *Pl-1*+ P-TGC layer in four lines (Fig. 5A; *Chp1*, *Urm1*, *Got2*, *Bltp2*), a thickened P-TGC layer in two lines (Fig. 5B; *Ppox*, *Alg3*), and a normal P-TGC layer in three lines (*Gmeb2, Kctd5*, *Rprd2*). In the remaining two lines (*Arpc1a and Zmym2)*, despite the initial identification of a thickened SpT layer using the analysis pipeline, the additional placentas examined displayed a normal SpT and P-TGC layer (Extended Data Fig. 5,15). While most KO lines with JZ defects also display LZ defects, *Zmym2*$^{-/-}$ placentas represent the only KO in which a JZ defect is not accompanied by a LZ defect (Extended Data Fig. 5)."

The updated phenotype categories in the placental phenotype summary (Fig 4 and legend) and the updated JZ specific Figure 5 are each copied below:

© 2026. Published by The Company of Biologists under the terms of the Creative Commons Attribution License (https://creativecommons.org/licenses/by/4.0/).

Figure 4: Summary of placental defects observed in all 22 KO lines.

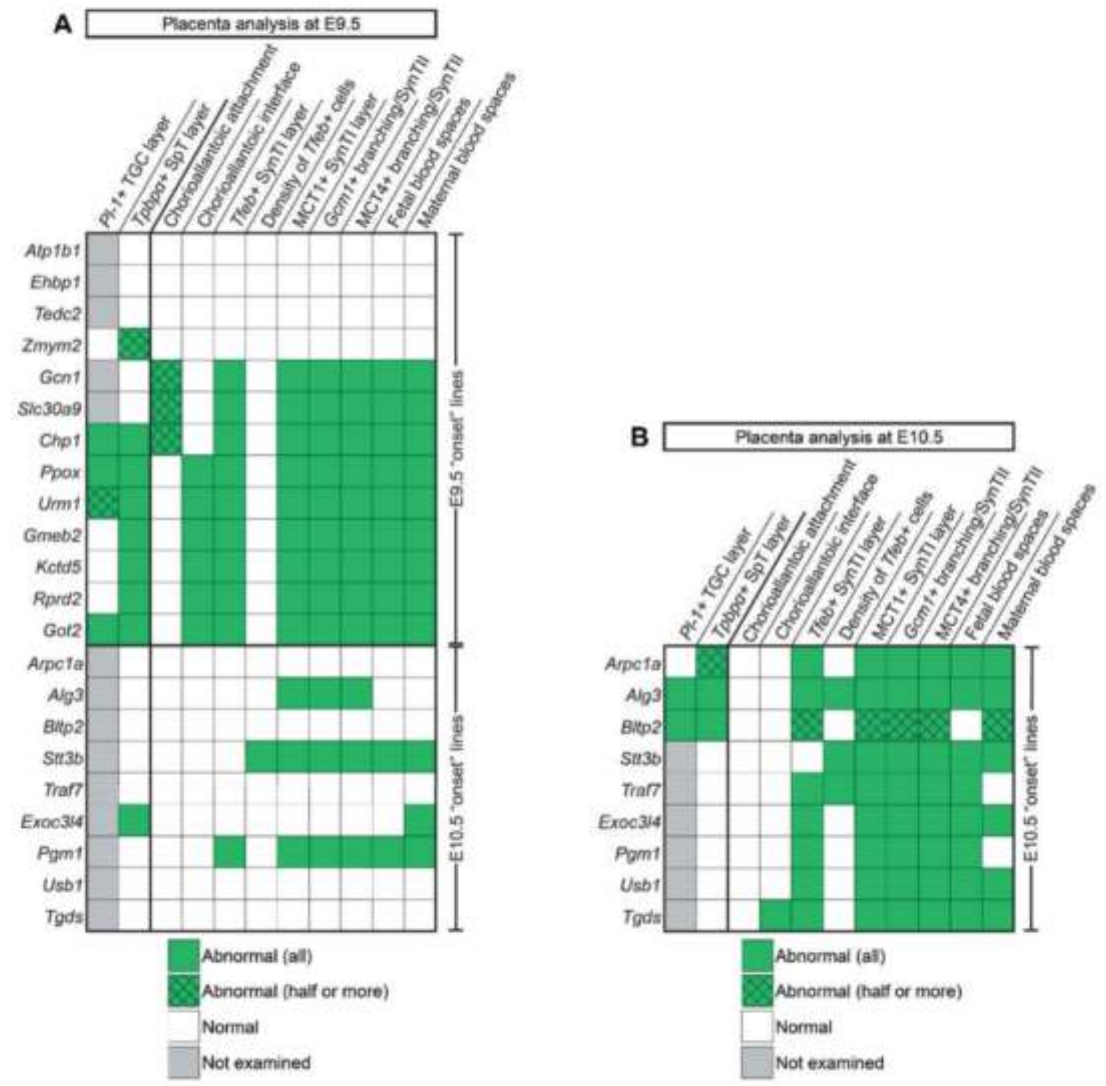

Figure 4: Summary of placental defects observed in all 22 KO lines. (A-B) KO placentas analyzed at E9.5 (A) and E10.5 (B) compared with littermate controls. Each phenotype was scored as present (green) if observed in all placentas analyzed (n¤3) while the patterned overlay indicates the phenotype was observed in at least half but not all KO placentas. The JZ was assessed for changes in the thickness of the *Tpbpa*+ SpT compartment relative to controls. For KO lines that exhibited an abnormal *Tpbpa*+ SpT layer, additional placentas were stained for *Pl-1* and TGC layer thickness assessed (grey shading = not assessed). LZ development examined as follows. The association of the allantois and chorion (chorioallantoic attachment) and the morphology of the resulting interface was examined by H&E. The thickness of both the *Tfeb*+ and MCT1+ domain indicate SynTI layer development while the intensity of *Tfeb* reflects trophoblast density. The extent and depth of *Gcm1* and MCT4 were used to assess chorioallantoic branching and SynTII layer development. H&E is used to assess the presence of discrete maternal and fetal blood spaces interspersed among labyrinth trophoblasts. The relative amounts of maternal and fetal blood in KO versus control placentas could not be reliably assessed qualitatively, except when differences were dramatic. The precise nature of the abnormality varies between KO lines and detailed descriptions are found in Extended Data Figures 2-23.

© 2026. Published by The Company of Biologists under the terms of the Creative Commons Attribution License (https://creativecommons.org/licenses/by/4.0/).

Figure 5: Junctional zone defects in mid-gestation lethal KO lines.

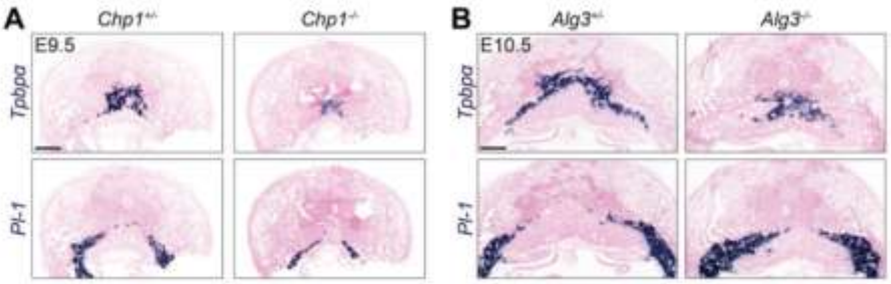

Figure 5: Junctional zone defects in mid-gestation lethal KO lines. *Tpbpa* and *Pl-1* expression in adjacent placental sections revealed SpT and TGC layer abnormalities, respectively. (A) E9.5 *Chp1*$^{-/-}$ placentas exhibit reduced SpT and TGC layers compared with controls. (B) E10.5 *Alg3*$^{-/-}$ placentas demonstrate a reduced SpT and expanded SpT layer. Scale bars: 500 µm.

The crux with Tfeb is that its endogenous expression pattern is not well defined. The stainings on the earlier placentas show that, in general, Tfeb stains the cell layer(s) just above Gcm1+ cells. The most likely interpretation is that Tfeb is expressed in an intermediate progenitor and that SynT-I cells emerge on the upper edges of the Tfeb+ domain. This would be in line with Tfeb being described as a SynT-I progenitor and SynT-I marker in the Marsh&Blelloch snRNA-seq study. What is almost certainly NOT the case is that Tfeb is a pan-labyrinth trophoblast marker, as described in the text, and the old reference does not provide sufficient resolution on this point. Ultimately, double stainings (IF or ISH) would be needed to fully resolve the question around what cell type(s) Tfeb demarcates exactly, but an inclusion of the above-mentioned MCT1/4 stainings would at least provide reasonable clarity on this point.

Thank you for your insight. Firstly, we no longer refer to *Tfeb* as a pan-labyrinth marker. All description of *Tfeb* and *Gcn1* expression has been updated. Furthermore, we have included an additional supplemental figure (SFig 3 below) with dot plots for *Tfeb, Gcm1* and *Tpbpa* from the scRNA datasets. These show that *Tpbpa* is most highly expressed in the SpT cluster and less abundantly in the SpT precursor, that *Gcm1* is robustly expressed in a subset of SynTII cells and SynTII precursors and that *Tfeb* is most highly expressed in SynTI precursors and a subcluster of SynT1 cells.

© 2026. Published by The Company of Biologists under the terms of the Creative Commons Attribution License (https://creativecommons.org/licenses/by/4.0/).

Supplemental Figure 3: Expression of *Tpbpa*, *Tfeb*, and *Gcm1* in trophoblast clusters from published RNA-seq datasets.

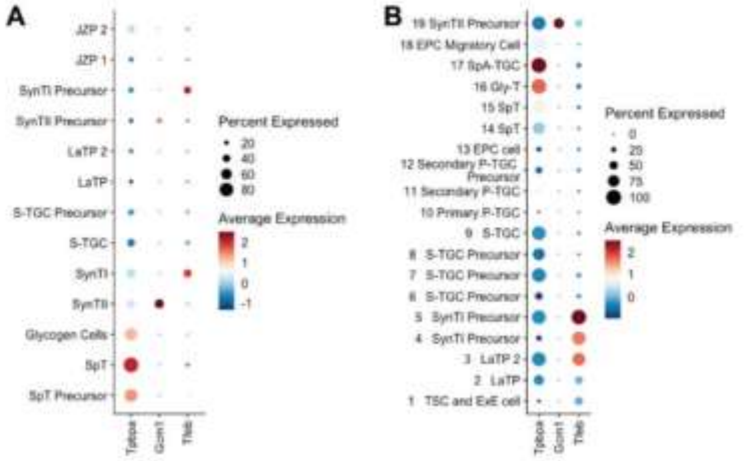

Because the MS revisions addressing these markers occur throughout the text we include a quote from the "Interhemal barrier development"section (lines 234-251) also see Figure 3 and legend (above) as examples of how we now discuss these markers :
"*5. Interhemal barrier development*

Interhemal barrier development coincides with chorioallantoic branching and the entry of fetal and maternal blood into the LZ. This barrier is composed of two layers of syncytiotrophoblasts (SynTI and SynTII) that separate endothelial-lined fetal blood spaces from maternal blood spaces which are bound by a perforated layer of S-TGCs [Fig. 3A; (Simmons et al., 2008)]. Individual layers cannot be identified without specific markers due to their close apposition. SynTII cells and their precursors express *Gcm1* [Fig. 3E, (Marsh and Blelloch, 2020; Simmons et al., 2008)]. SynTII layer development coincides with chorioallantoic branching, as SynTII cells differentiate and outline the allantoic mesenchyme, carrying fetal vasculature, as it integrates into the labyrinth and subsequently expands through further branching. Mature SynTII cells also express MCT4 [Fig. 3F/G; (Nagai et al., 2010)]. While *Gcm1* and MCT4 expression largely overlap, MCT4 appears more continuous along the SynTII layer whereas *Gcm1* expression is more discontinuous and appears restricted to a subset of SynTII cells (E9.25-E10.5, Fig. 3E-F). SynTI cells and their precursors express *Tfeb* [Fig. 3C; (Marsh and Blelloch, 2020)]. Mature SynTI cells also express MCT1 (Fig. 3D, G). *Tfeb* and MCT1 expression appear only partially overlapping. MCT1 is enriched at the JZ border and is absent from the more basal chorionic trophoblasts that express *Tfeb*, suggesting these basal cells represent the precursors that give rise to the MCT1+ SynTI cells (Fig. 3C, D, G)."

Following on from the not-well-defined expression domain of Tfeb, the authors describe that in some mutants, Gcm1+ cells seem to "reach through" the Tfeb+ region. This, however, appears to happen during normal development between E9.5-E10.0, which can be seen in Extended Figure 1 as well as in Figure 3.

Agreed. We do believe that this is a normal process. In the "intrahemal barrier formation" section of the MS we state (line 252-257) "As interhemal barrier development proceeds, MCT1+ SynTI cells and maternal blood initially restricted to the JZ boundary, together with *Gcm1*/MCT4+ SynTII lined fetal vasculature adjacent to the chorioallantoic interface, progressively integrate and extend into the developing labyrinth. By E10.5, an extensive network of adjacent SynTI, SynTII, and endothelial cells is observed (Fig. 3G), along with closely apposed maternal and fetal blood spaces (Fig. 3H, J', arrowhead)."

In the results section termed "LZ defects in E10.5 onset lines" (line 320-328) we state that "Although chorioallantoic branching and primary villi formation have initiated, as evidenced by

© 2026. Published by The Company of Biologists under the terms of the Creative Commons Attribution License (https://creativecommons.org/licenses/by/4.0/).

the presence of chorionic involution and the shallow integration of allantoic mesenchyme into the LZ (Fig 6D-F, arrows), the reduction of *Gcm1*/MCT4+ branches indicate impaired villi elongation. It is worth noting that in many of these KO placentas (5 of 9), the *Gcm1*/MCT4+ branches that are present appear to normally extend through the *Tfeb*+ SynTI precursor layer to reach the MCT1+ SynTI cells, although they are dramatically reduced in number (Fig. 6D). In the remaining lines (4 of 9), *Gcm1*/MCT4 branching fails to traverse the *Tfeb*+ SynTI precursor layer and are rarely in contact with MCT1+ SynTI cells (Fig. 6E, F)."

Hence, the Exoc3l4-mutant phenotype may be an indicator of premature differentiation of certain trophoblast subtypes. The phenotype description should be reworded to leave this possibility open, and/or the text re-evaluated after the above stainings have been performed.

We do not understand the context of this question and so it has not been addressed.

The reference to the Placenta Atlas, or even "newly developed" Placenta Atlas, should be reworded to something like "Characterizing placental development across a mid-gestational time course", or similar. The Placenta Atlas terminology is somewhat aggrandized; while it is very nice and was a lot of work, there is nothing new to this compilation, even with the gene expression patterns which have largely been reported in Simmons et al. 2008. Also, the quarter-day staging is likely inaccurate and perhaps an 'overkill', see for example E8.75 vs E9.0 where placental development may have been captured in a discontinuous way (more domed chorion morphology of the E9.0 placenta compared to the E8.75, in theory it should be inverse).

As stated in our comments to reviewer 1 (above), we no longer use the terms "novel" or "atlas" to describe our characterization of normal placentation during mid-gestation.

The manuscript states that serial placental sections were used for the histology analysis. Clicking through their citeddatabase, this is not always the case; in fact it is quite frequently not the case.

There are two exceptions to the serial section analysis. Because we were unable to reliably obtain 4 or 5 sequential midline sections from E8.5 embryos, we performed individual histological or molecular assays on complete E8.5 placentas and thus any E8.5 sections included in the developmental time-course and in our blog are each from a different placenta.
This sentence is in the Figure legend of Fig 3 "Because size limitations, all sections of an E8.5 placenta were subjected to a single ISH probe or H&E staining"
Because the *Slc30a9* mutants were consistently delayed at E9.5, these placentas were the only later stage exception to the serial section analysis. This information is clearly stated in the extended data figure for *Slc30a9* (Extended Data Figure 7) and is represented on the blog. Two of us reviewed the blog and all mutant/control placenta sections presented (except those noted above) represent serial section analysis.

Finally, we should note that within the normal developmental time course (Fig3), the E9.0-9.5 Tpbpa sections are from a different placenta than the remainder of the analysis in that column. The figure legend now states this. "Because size limitations, all sections of an E8.5 placenta were subjected to a single ISH probe or H&E staining, however for E8.75-E10.5 placentas, adjacent sections was used for all assays except where noted. (B) *Tpbpa* expression in E8.5-E10.5 placentas demonstrates the developmental progression of the SpT layer. The E9.0-9.5 sections are from a different placenta than the remainder in their respective columns."

The authors conclude that 19 out of the 22 lines they investigated have a placental phenotype. However, the other 3 lines are those that die even earlier. Therefore, it is possible that these, too, have an extraembryonic phenotype in the trophoblast lineage or in the yolk sac, but the stages assessed and markers employed would not capture this. Hence, it might be better to say that those 19 with a mid-gestational lethality all have a placental phenotype, and refer to the other three as an earlier-onset lethality.

© 2026. Published by The Company of Biologists under the terms of the Creative Commons Attribution License (https://creativecommons.org/licenses/by/4.0/).

Indeed, a closer inspection of all three lines seems to show a lack of fetal blood in the developing placenta, and at least for Atp1b1 and perhaps Tedc1 a delamination of the visceral yolk sac endoderm from the mesoderm (i.e., the blood islands are not as clearly defined as in the controls). For Ehbp1, the histology is too poor to tell. The authors can either re-check their sections to determine YS phenotypes, and/or leave this possibility open that other extra-embryonic phenotypes may be present.
We have left that door open. The following comment has been added (lines 266-270) "All three of the lines with no identified placental defects were in the "E9.5 onset" category (*Atp1b1*$^{-/-}$, *Ehbp1*$^{-/-}$, *Tedc2*$^{-/-}$; Fig. 4A, Extended Data Fig. 2-4), exhibited delay and pronounced embryonic defects at E9.5 (Fig. 1A), suggesting that at least through E9.5, a grossly abnormal embryo does not impact the aspects of placental development examined herein."

The following is now the penultimate paragraph of the discussion "The streamlined approach used herein has identified specific placental defects that coincide with early mid-gestion embryo phenotypic onset in most of KO lines examined, however it is not without limitations. The analysis pipeline did not molecularly assess the YS and thus it is possible that YS or trophectoderm-specific defects contribute to embryonic demise. Similarly, our analysis does not directly examine the S-TGC that remodel maternal vasculature and line channels that deliver maternal blood into the labyrinth, in our approach their differentiation is indirectly inferred by the presence of maternal blood in the trophoblast compartment. Nonetheless, the approach presented herein provides a wealth of information using cell-type specific analyses, making it well suited for identifying many aspects of the mid-gestation placenta."

Other wording issues:
E9.5-onset phenotype: This is incorrect, since several of these lines already have an overt phenotype at E9.5 and even earlier at E8.5. So the "onset" is not at E9.5, it should rather be "E9.5 phenotype" lines.

We appreciate this comment. Coming up with names for our two broad embryo phenotype categories was difficult. Because of the clear wiggle room in the overall categorization process, we now use the term "E9.5 onset" and "E10.5 onset" lines always in quotes to denote those caveats. We have re-written the descriptor section (lines 117-130)

"The gross embryonic phenotyping results for mutant from all lines are summarized in Fig. S2 and can be found in more detail here (https://websites.umass.edu/kdtkomp/). Broadly, the embryonic phenotype produced by each KO line was placed in two overarching categories, determined by gross phenotype onset. The first includes lines that were delayed or small at E8.5. By E9.5 mutants from each of these lines failed to appropriately turn and all mutants from each line displayed gross phenotypes that distinguish them from their littermates (termed "E9.5 onset"; n=13/22 lines; Fig. 1A). This category includes 5 lines that were uniformly smaller or delayed compared to E8.5 littermates (*Chp1, Gcn1, Slc30a9, Rprd2, Urm1*; Fig. 1A, S1A, S2). In the second category, mutants from all lines initiated turning by E9.5. Although the majority of mutants from many of these lines could not be distinguished from their E9.5 littermates, by E10.5 all lines display overall gross defects and several experienced a rapid morphological decline ("E10.5 onset"; n=9/22 lines; Fig. 1B, S2). Together, this analysis classifies the null embryonic phenotypes produced by each of the 22 KO lines analyzed herein into two broad descriptors, henceforth termed "E9.5 onset" or "E10.5 onset". "

Lines 73-74, fix sentence structure.
Changed to: "A large-scale analysis of KO lines that produce lethality during a similar window of murine development suggests that placentation is a critical bottleneck for development past mid-gestation."

Line 94, "phenotypes" (plural)
Fixed.

Line 108 and other places, "heterozygous KO" is an oxymoron
Thank -you, this has been changed throughout.

© 2026. Published by The Company of Biologists under the terms of the Creative Commons Attribution License (https://creativecommons.org/licenses/by/4.0/).

Line 128 and elsewhere, what is an "intermediate lethal KO line"?

This is the official IMPC designation for this stage of lethal lines (present at 9.5 absent at E12.5). We have eliminated this terminology in the manuscript.

Line 129, see also above, the "onset" problem
See description above.

Lines 241/242, it is exceedingly difficult - if not impossible - to make out the two syncytiotrophoblast layers in H&E stainings. Hence the recommendation about the added MCT1/4 staining detailed above. Also, what is an "attenuated morphology"?

This comment has been addressed by the inclusion of MCT1/4 IF as noted above. We now use "elongated" instead of "attenuated."
Lines 256/257, and also Discussion lines 387-391, please see above about YS defects (or other, earlier trophoblast defects).

The following comment has been added to lines 266-270 "All three of the lines with no identified placental defects were in the "E9.5 onset" category (*Atp1b1*$^{-/-}$, *Ehbp1*$^{-/-}$, *Tedc2*$^{-/-}$; Fig. 4A, Extended Data Fig. 2-4) and exhibited delay and pronounced embryonic defects at E9.5 (Fig. 1A) suggesting that at least through E9.5, a grossly abnormal embryo does not impact the aspects of placental development examined herein."

The following is now the penultimate paragraph of the discussion "The streamlined approach used herein has identified specific placental defects that coincide with early mid-gestion embryo phenotypic onset in most of KO lines examined, however it is not without limitations. The analysis pipeline did not molecularly assess the YS and thus it is possible that YS or trophectoderm-specific defects contribute to embryonic demise. Similarly, our analysis does not directly examine the S-TGC that remodel maternal vasculature and line channels that deliver maternal blood into the labyrinth, in our approach their differentiation is indirectly inferred by the presence of maternal blood in the trophoblast compartment. Nonetheless, the approach presented herein provides a wealth of information using cell-type specific analyses, making it well suited for identifying many aspects of the mid-gestation placenta."

JZ defects section: Whether or not the JZ is expanded or reduced makes most sense in conjunction with knowledge of TGC differentiation. Most commonly, mouse lines with a smaller EPC/JZ have an overabundance or over-enlargement of TGCs. Historically, this bifurcation is exemplified by the Ascl2 mutant (precocious exit from a mitotic cell cycle to an endoredupplicative cell cycle, i.e. more TGCs at the expense of diploid SpT cells).

Thank-you for your insight. As noted above, new placentas from 11 of the 12 lines with previously noted *Tpbpa* defects have been analyzed for both *Pl-1* and *Tpbpa*. The new information has been integrated into the MS as noted above.

Lines 310/311, the lollipops appear to highlight maternal blood spaces, please verify that these are indeed fetal, as described.

We are certain that the lollipops are on fetal blood spaces and this should be more apparent in the high-resolution images supplied.

Lines 336/337, "sectioned ISH", is this ISH on sections, or whole-mount ISH sectioned? Presumably the former.

Section *in situ* hybridization. This has been corrected.

© 2026. Published by The Company of Biologists under the terms of the Creative Commons Attribution License (https://creativecommons.org/licenses/by/4.0/).

Second decision letter

MS ID#: dev.205276R1

MS TITLE: Onset of embryonic and placental defects coincide in 19 of 22 novel mid-gestation lethal murine knockout lines

AUTHORS: Kimberly D. Tremblay, Taylor M. Guertin, Jesse Mager, Katrien De Clercq, Emily L. Pease, Chip Sisson, Olivia Macrorie, Hannah E. Garth, Rossella M. Gargiulo and Joris Vriens

Dear Kim, The overall evaluation is very positive and we would like to publish a revised manuscript in Development, provided you can address the minor issues raised by Reviewer 1. Please attend to all of the reviewers' comments in your revised manuscript and detail them in your point-by-point response. If you do not agree with any of their criticisms or suggestions explain clearly why this is so. Your manuscript will not require any further review, rather I will accept it after you upload the final version.

Reviewer 1

Line 77 should read 'extra-embryonic derived mesoderm gives rise to the allantois'

While SFig2 has been amended to include Fisher's tests for embryonic phenotyping this was not performed by the authors for placental phenotyping (Fig4), which I think is a missed opportunity to give more weight to the findings. My query illuminated that while embryonic phenotyping used n >7, placental phenotyping was only n=3 for KOs (from lines 112-113 - is this correct?), which is low number for F tests. It should be mentioned somewhere in the manuscript that this experiment is underpowered. N numbers for this experiment should also be mentioned in the Fig4 legend.

While mentioned in the Response to Reviewers, I did not see in the manuscript where it was mentioned that control placentas (littermate controls) were assumed to be 'normal'. This is a sizable assumption which needs mentioning.

My other comments have been addressed.

Reviewer 2

THe authors have made a superb effort to address all reviewers' comments raised, and have included multiple new stainings to improve the interpretation of their findings. Other wording issues that were flagged have also been addressed.
This is a very comprehensive study of the placental phenotypes in 22 mid-gestational lethal mouse mutant lines, that will be a wonderful resource for the wider community to better understand the impact of extra-embryonic tissues on embryonic development and add to the list of genes that are indispensable in extra-embryonic tissues.

Second revision

Author response to reviewers' comments

We have addressed the concerns as outlined below. The smaller italicized text is the reviewers' critique and the larger text my response. Any altered text is highlighted in the Manuscript and bolded in my response below.

Reviewer 1: Line 77 should read 'extra-embryonic derived mesoderm gives rise to the allantois'

© 2026. Published by The Company of Biologists under the terms of the Creative Commons Attribution License (https://creativecommons.org/licenses/by/4.0/).

Line 77 now reads “In the mouse, placentation initiates at E8.25 when the **extra-embryonic mesoderm-derived allantois**, extending from the posterior primitive-streak, attaches to the trophectoderm-derived chorion,

While SFig2 has been amended to include Fisher's tests for embryonic phenotyping this was not performed by the authors for placental phenotyping (Fig4), which I think is a missed opportunity to give more weight to the findings. My query illuminated that while embryonic phenotyping used n >7, placental phenotyping was only n=3 for KOs (from lines 112-113 - is this correct?), which is low number for F tests.

We did not perform a Fisher’s test because any phenotype call in a mutant placenta was assessed by directly comparing it to a placenta of a single similarly staged littermate control. With the embryonic phenotyping example all mutants were compared with all control embryos and thus an F test was appropriate.

It is correct that a minimum of 3 mutant and control comparisons were made.

It should be mentioned somewhere in the manuscript that this experiment is underpowered. N numbers for this experiment should also be mentioned in the Fig4 legend.

I don’t think that we need to say that it is underpowered because we clearly articulate how many placentas were examined throughout the manuscript. The first example was edited but the remainder were already in the text.

Line 261-265 (beginning of placental phenotype description in results) now reads:
“The key morphological events described above were assessed in KO placentas and directly compared with littermate controls from all 22 lines at E9.5 (Fig. 4A). The “E10.5 onset” lines were **similarly** examined at E10.5 (Fig. 4B). **Representative images of KO and littermate control placentas of each line, including the number examined,** is provided in more detail (Extended Data Fig. 2-23). Lines 544-46 In Figure 4 legend:
“Each phenotype was scored as present (green) if observed in all placentas analyzed ($n^{3}3$) while the patterned overlay indicates the phenotype was observed in at least half but not all KO placentas.”

Line 748-50 in the Methods:
“A KO line was considered to exhibit a particular phenotype (Fig. 4, see figure legend for assessment criteria) if the abnormality was observed in all placentas analyzed (minimum of n=3) unless otherwise stated.”

While mentioned in the Response to Reviewers, I did not see in the manuscript where it was mentioned that control placentas (littermate controls) were assumed to be 'normal'. This is a sizable assumption which needs mentioning.

We extensively documented placental development in Figure 3 and Extended Data Figure 1, and used the same markers when comparing our controls to KO. While we don’t state that each control is normal, we are in possession of a large bank of control placental tissue and did not identify any large discrepancies with any of our control placentas.

My other comments have been addressed.

<u>Third decision letter</u>

MS ID#: dev.205276R2

MS TITLE: Onset of embryonic and placental defects coincide in 19 of 22 novel mid-gestation lethal murine knockout lines

© 2026. Published by The Company of Biologists under the terms of the Creative Commons Attribution License (https://creativecommons.org/licenses/by/4.0/).

AUTHORS: Kimberly D. Tremblay, Taylor M. Guertin, Jesse Mager, Katrien De Clercq, Emily L. Pease, Chip Sisson, Olivia Macrorie, Hannah E. Garth, Rossella M. Gargiulo and Joris Vriens

Dear Kim, I am happy to tell you that your manuscript has been accepted for publication in Development, pending our standard publication integrity checks.

© 2026. Published by The Company of Biologists under the terms of the Creative Commons Attribution License (https://creativecommons.org/licenses/by/4.0/).
